# Supplementary material for: Azoramide ameliorates cadmium-induced cytotoxicity by inhibiting endoplasmic reticulum stress and suppressing oxidative stress
Source: PeerJ. 2024 Jan 31;12:e16844. doi: 10.7717/peerj.16844 (PMC10838077; doi:10.7717/peerj.16844)
Supplement: Supplemental Information 1 — Files in .PZFX format can be accessed using GraphPad Prism software (version 9.0; San Diego, CA, USA) which can be downloaded from https://www.graphpad.com/. [file peerj-12-16844-s001.zip › Supplemental Files/Western blot raw data.pptx]

## Slide 1
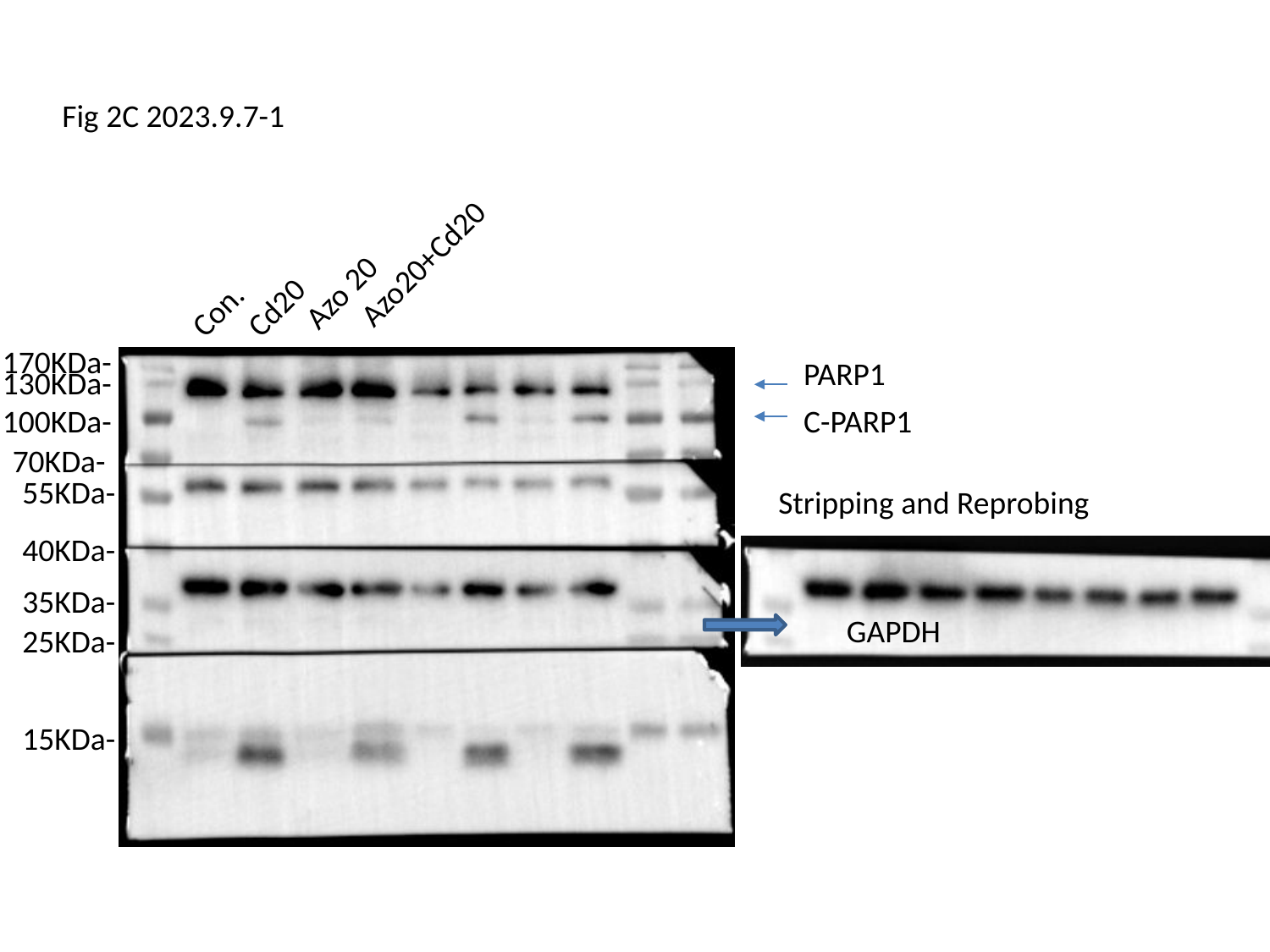

Fig 2C 2023.9.7-1
Azo20+Cd20
Cd20
Azo 20
Con..
170KDa-
130KDa-
100KDa-
70KDa-
55KDa-
40KDa-
35KDa-
25KDa-
15KDa-
PARP1
C-PARP1
Stripping and Reprobing
GAPDH

## Slide 2
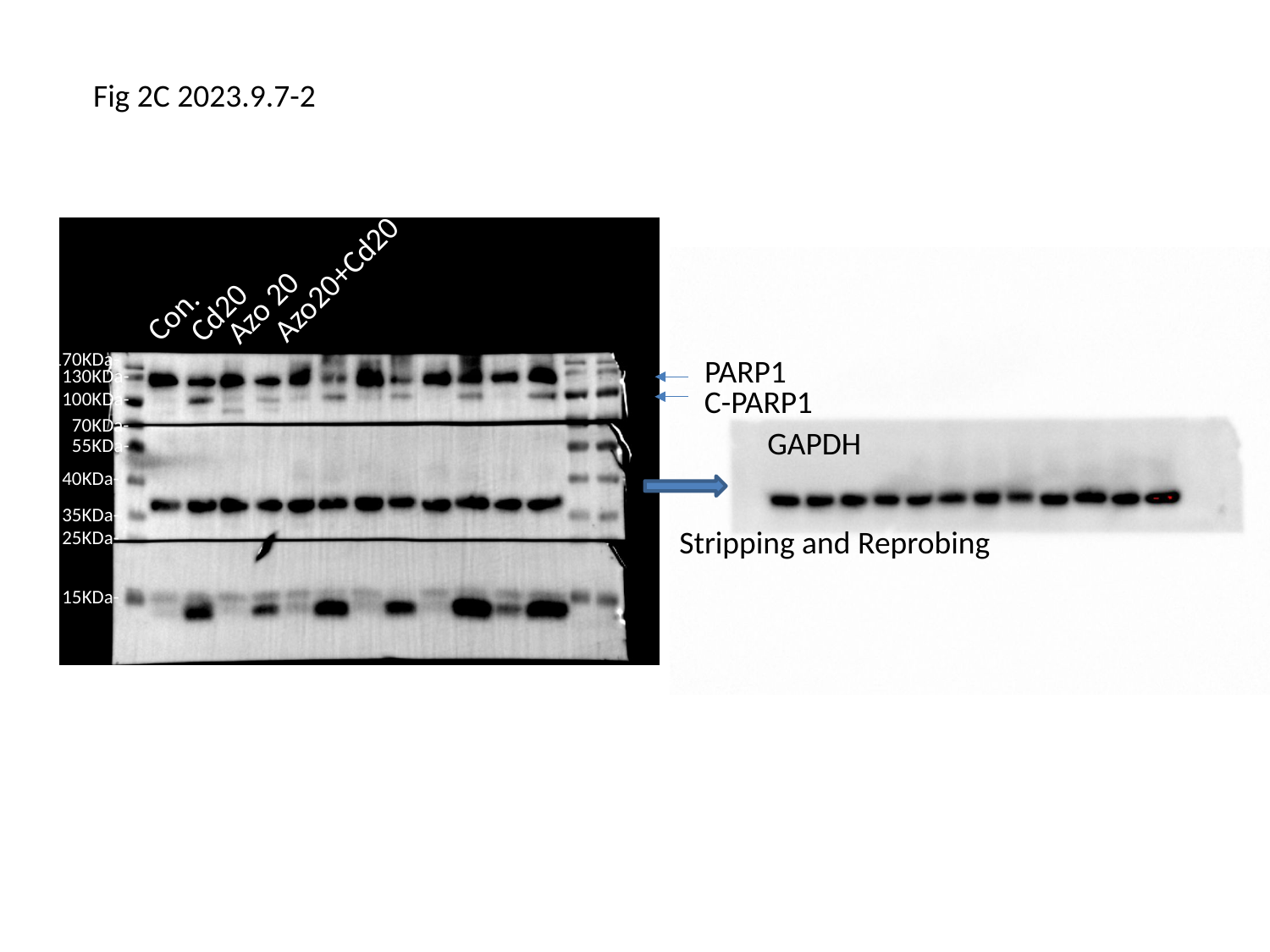

Fig 2C 2023.9.7-2
Azo20+Cd20
Cd20
Azo 20
Con.
170KDa-
PARP1
130KDa-
C-PARP1
100KDa-
70KDa-
GAPDH
55KDa-
40KDa-
35KDa-
Stripping and Reprobing
25KDa-
15KDa-

## Slide 3
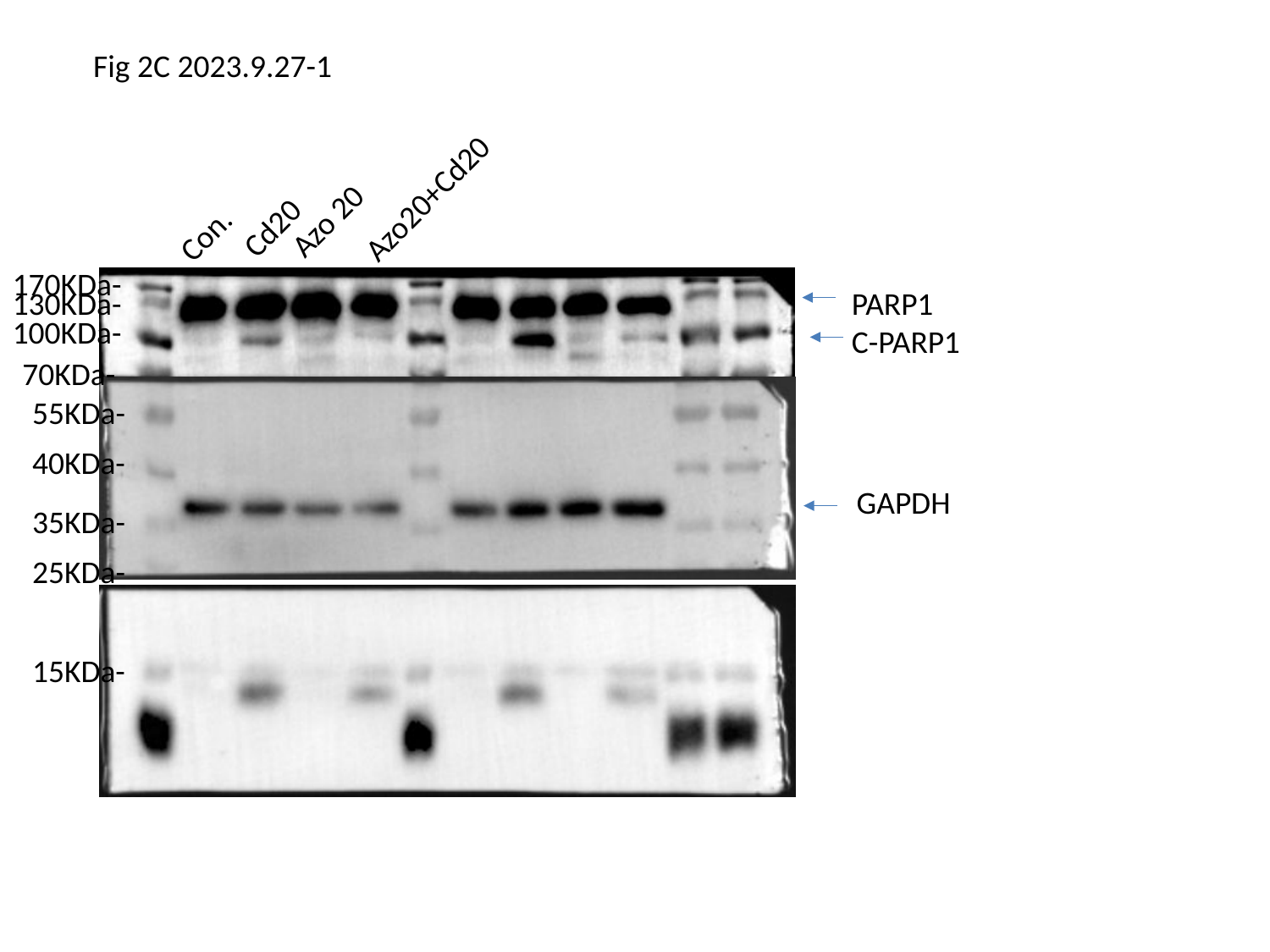

Fig 2C 2023.9.27-1
Azo20+Cd20
Cd20
Azo 20
Con.
170KDa-
130KDa-
PARP1
100KDa-
C-PARP1
70KDa-
55KDa-
40KDa-
GAPDH
35KDa-
25KDa-
15KDa-

## Slide 4
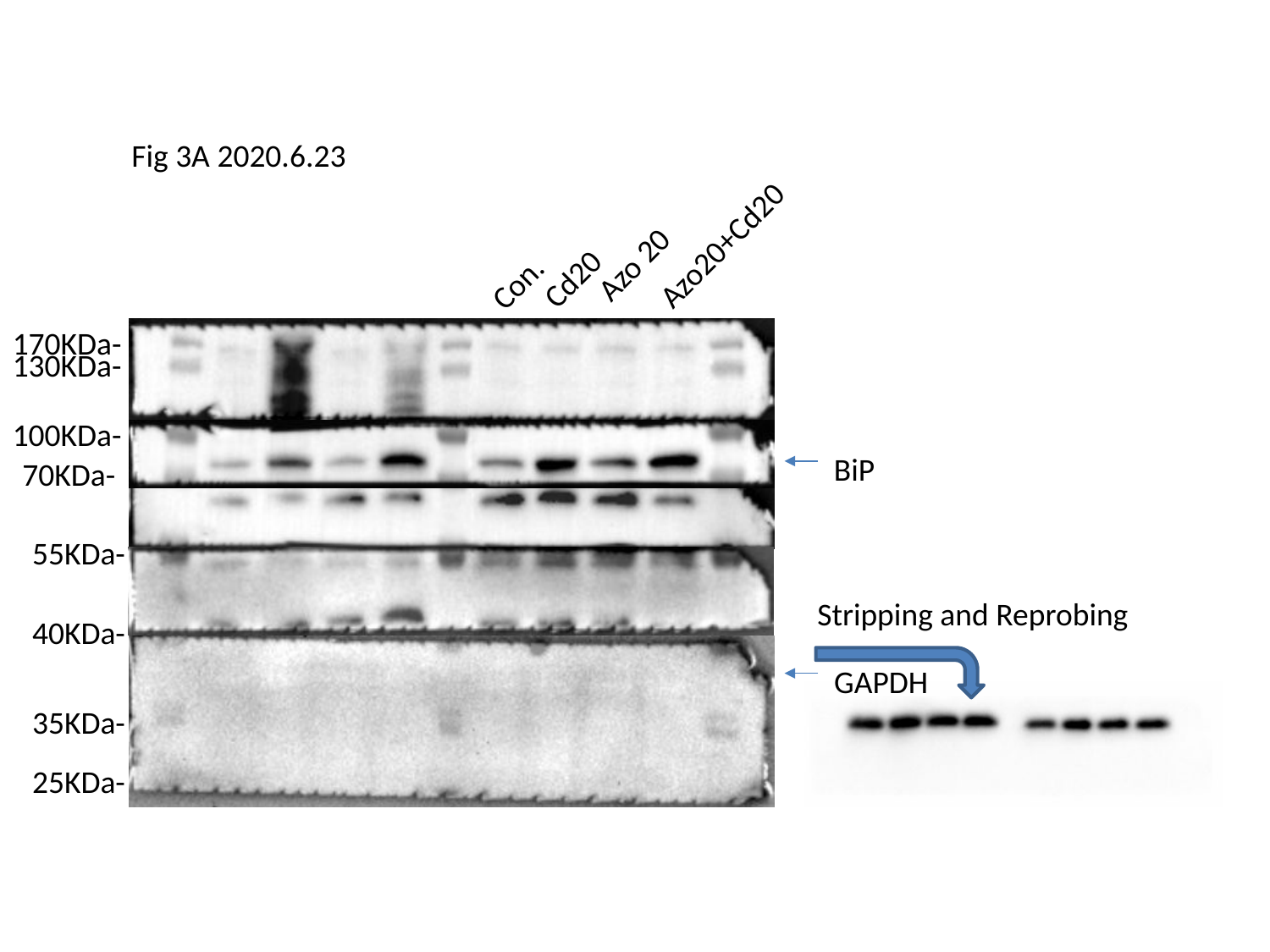

Fig 3A 2020.6.23
Azo20+Cd20
Cd20
Azo 20
Con.
170KDa-
130KDa-
100KDa-
70KDa-
55KDa-
40KDa-
35KDa-
25KDa-
Stripping and Reprobing
BiP
GAPDH

## Slide 5
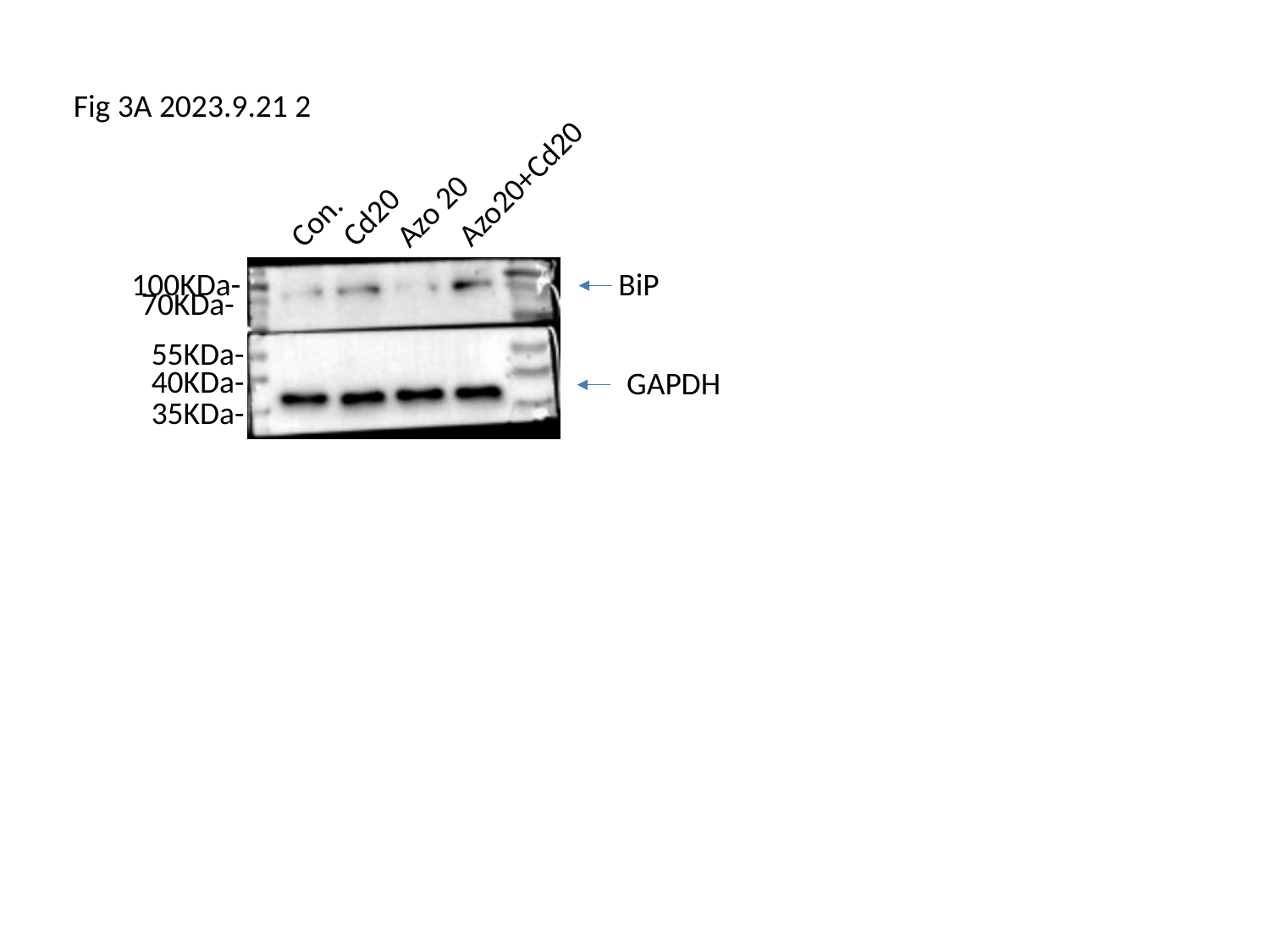

Fig 3A 2023.9.21 2
Azo20+Cd20
Cd20
Azo 20
Con.
100KDa-
BiP
70KDa-
55KDa-
40KDa-
GAPDH
35KDa-

## Slide 6
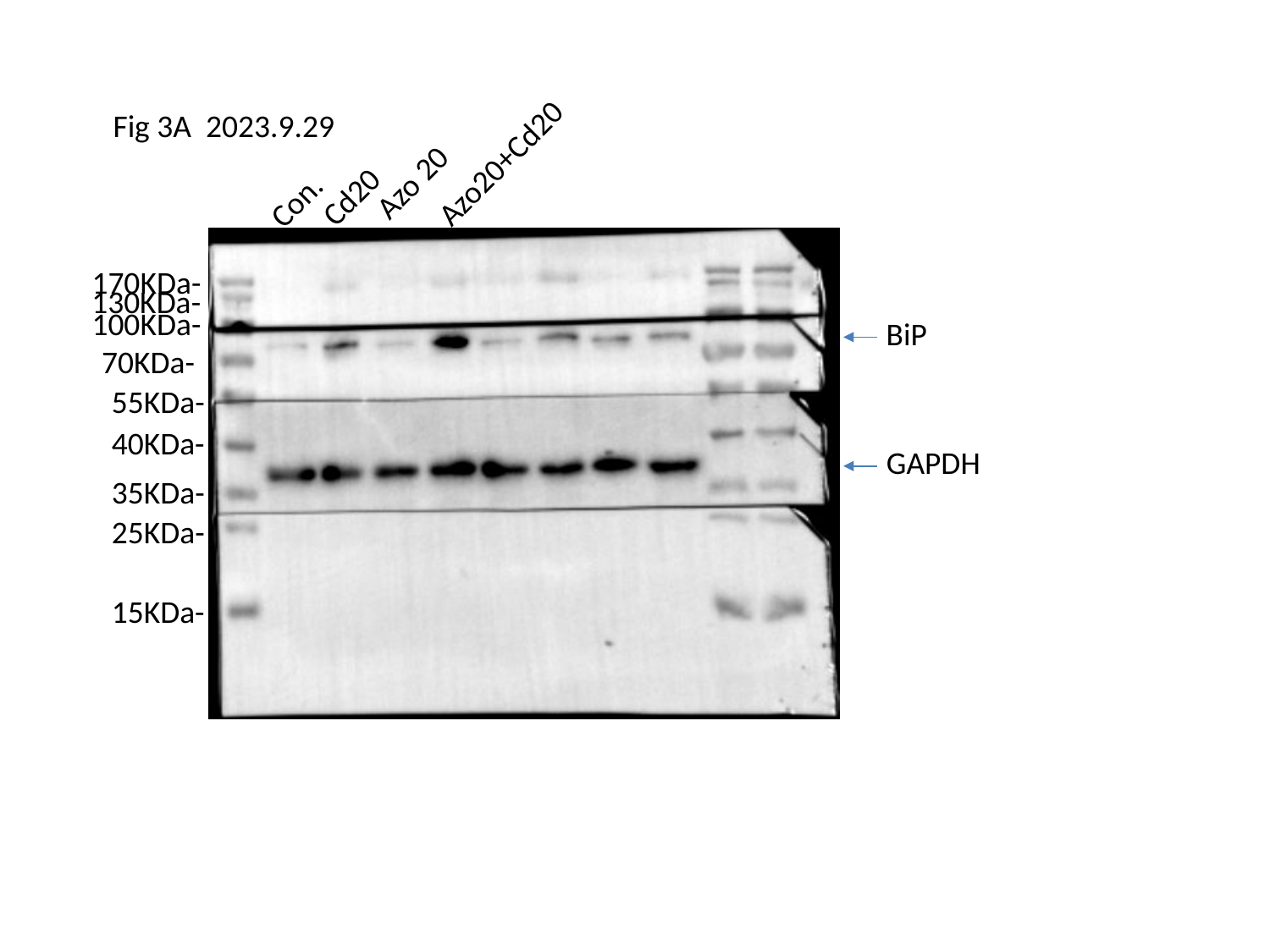

Fig 3A 2023.9.29
Azo20+Cd20
Cd20
Azo 20
Con.
170KDa-
130KDa-
100KDa-
BiP
70KDa-
55KDa-
40KDa-
GAPDH
35KDa-
25KDa-
15KDa-

## Slide 7
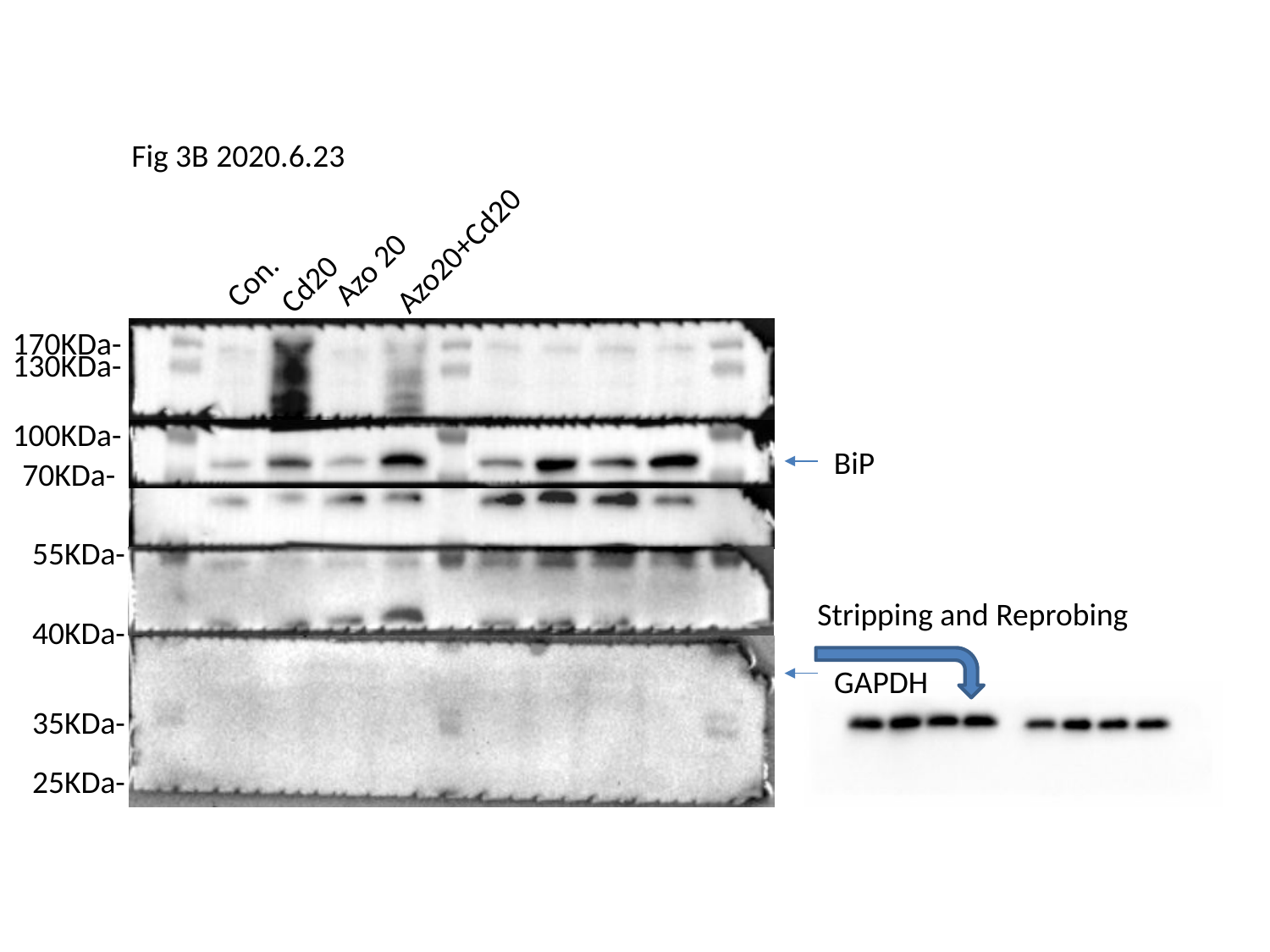

Fig 3B 2020.6.23
Azo20+Cd20
Cd20
Azo 20
Con.
170KDa-
130KDa-
100KDa-
70KDa-
55KDa-
40KDa-
35KDa-
25KDa-
Stripping and Reprobing
BiP
GAPDH

## Slide 8
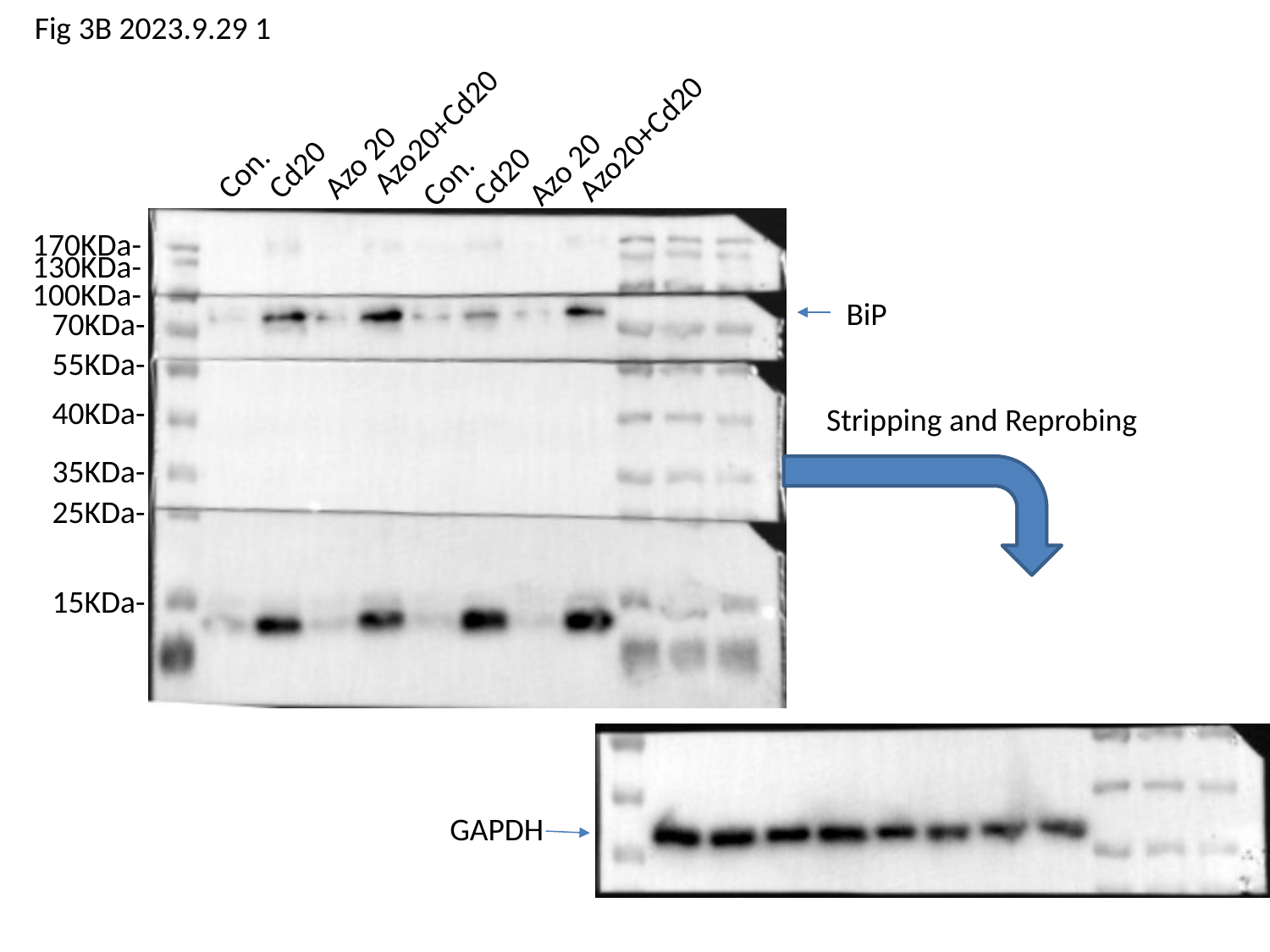

Fig 3B 2023.9.29 1
Azo20+Cd20
Azo20+Cd20
Cd20
Cd20
Azo 20
Azo 20
Con.
Con.
170KDa-
130KDa-
100KDa-
BiP
70KDa-
55KDa-
40KDa-
Stripping and Reprobing
35KDa-
25KDa-
15KDa-
GAPDH

## Slide 9
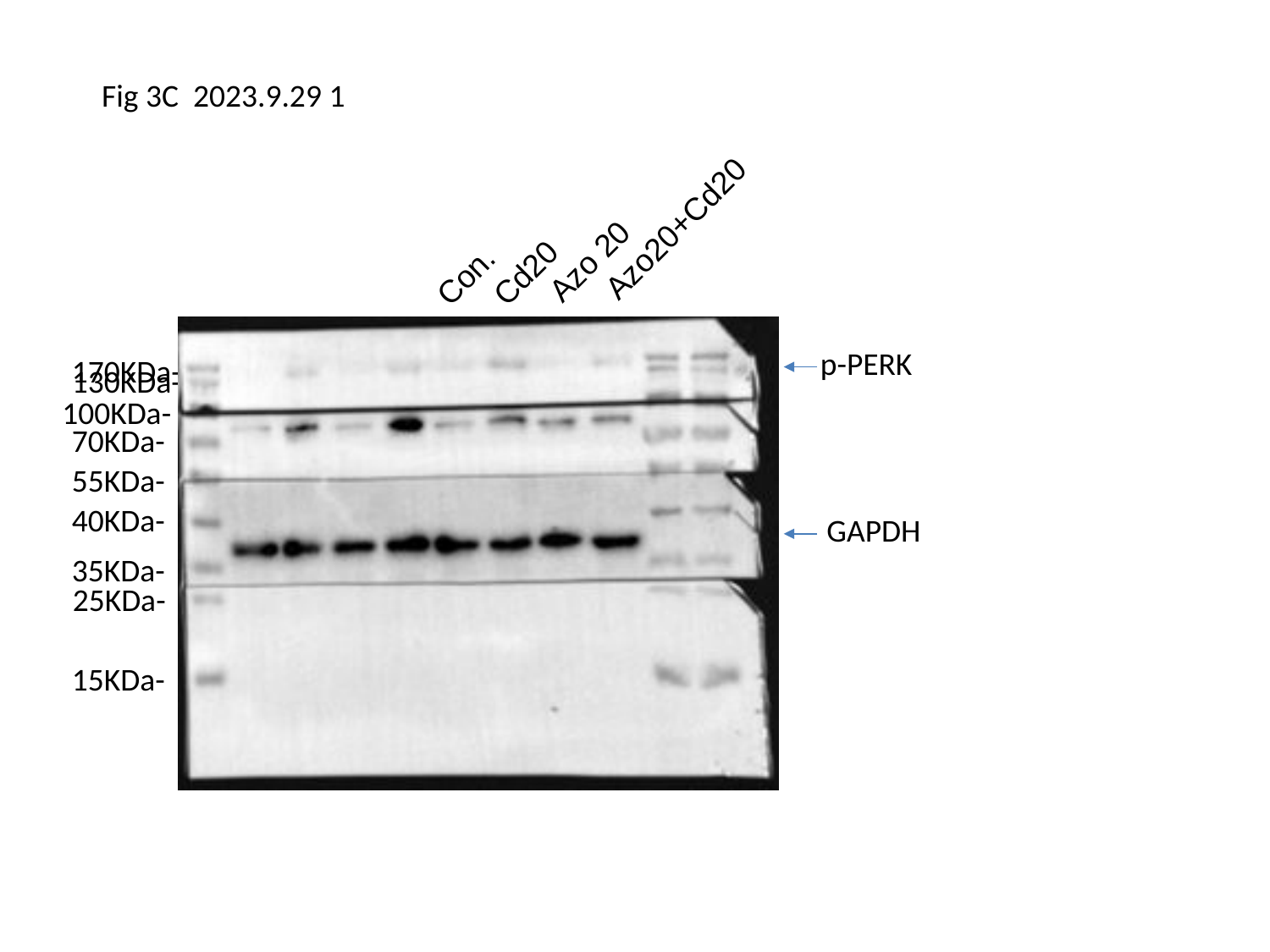

Fig 3C 2023.9.29 1
Azo20+Cd20
Cd20
Azo 20
Con.
p-PERK
170KDa-
130KDa-
100KDa-
70KDa-
55KDa-
40KDa-
GAPDH
35KDa-
25KDa-
15KDa-

## Slide 10
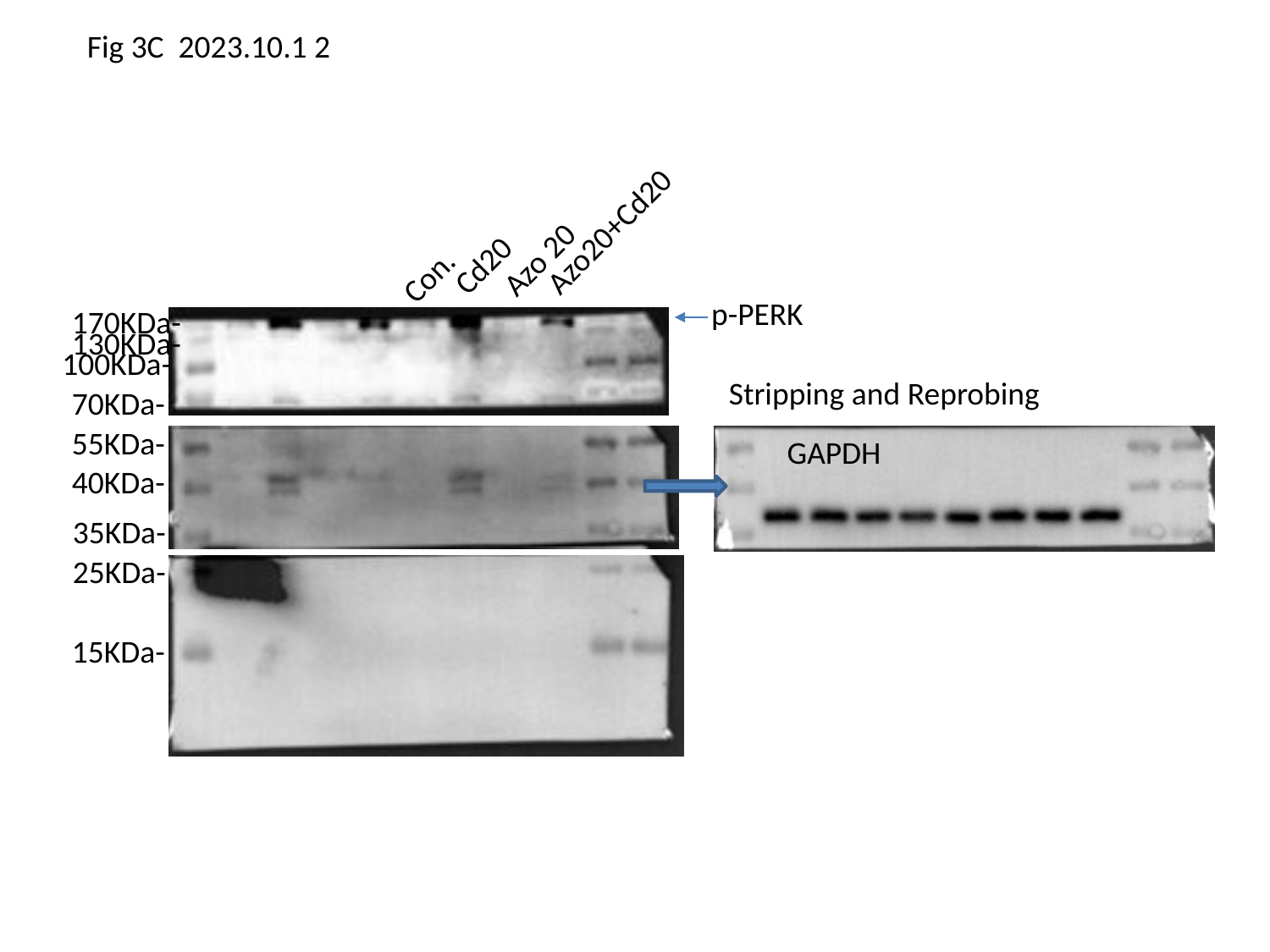

Fig 3C 2023.10.1 2
Azo20+Cd20
Cd20
Azo 20
Con.
p-PERK
170KDa-
130KDa-
100KDa-
Stripping and Reprobing
70KDa-
55KDa-
GAPDH
40KDa-
35KDa-
25KDa-
15KDa-

## Slide 11
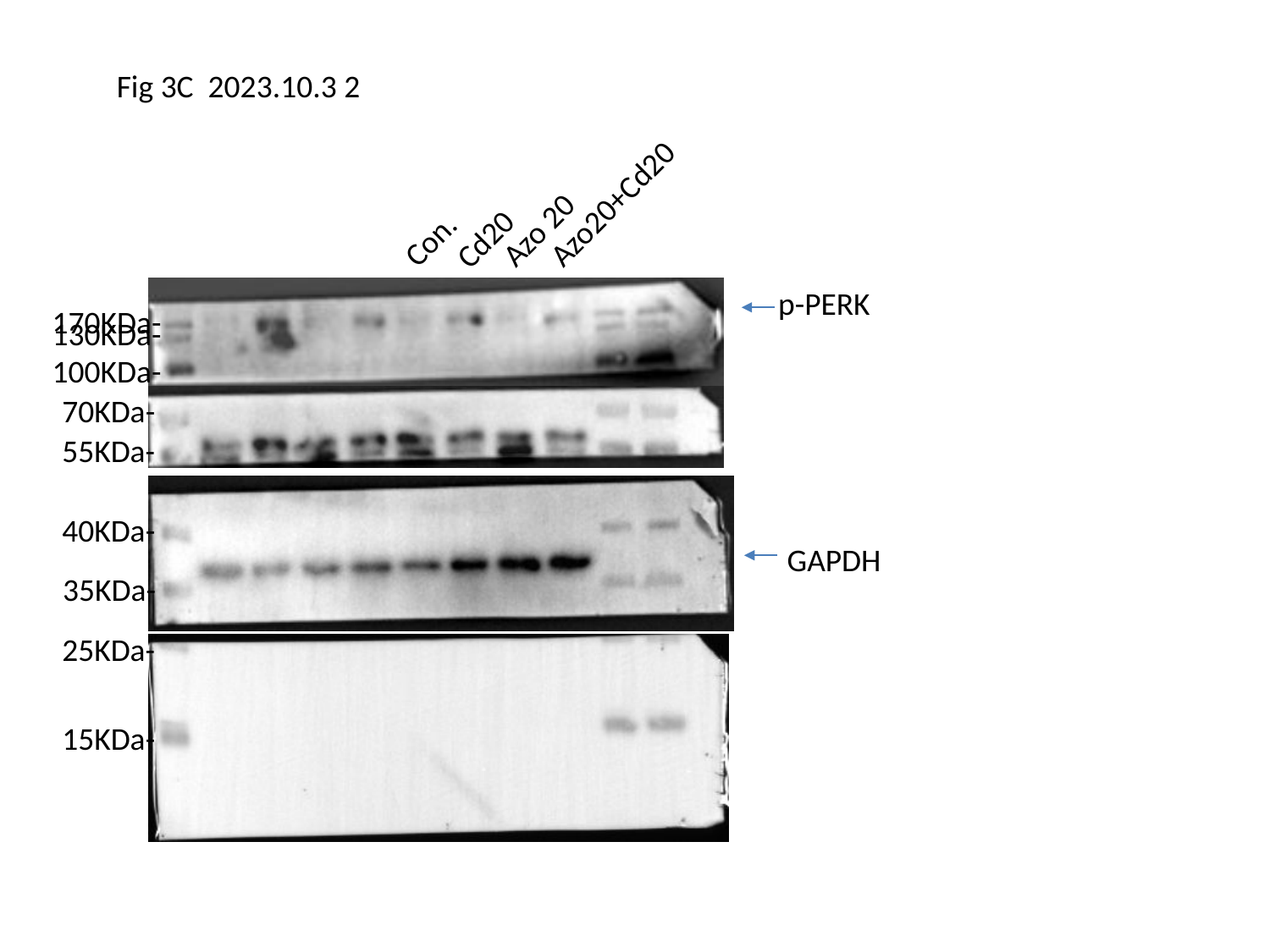

Fig 3C 2023.10.3 2
Azo20+Cd20
Cd20
Azo 20
Con.
p-PERK
170KDa-
130KDa-
100KDa-
70KDa-
55KDa-
40KDa-
GAPDH
35KDa-
25KDa-
15KDa-

## Slide 12
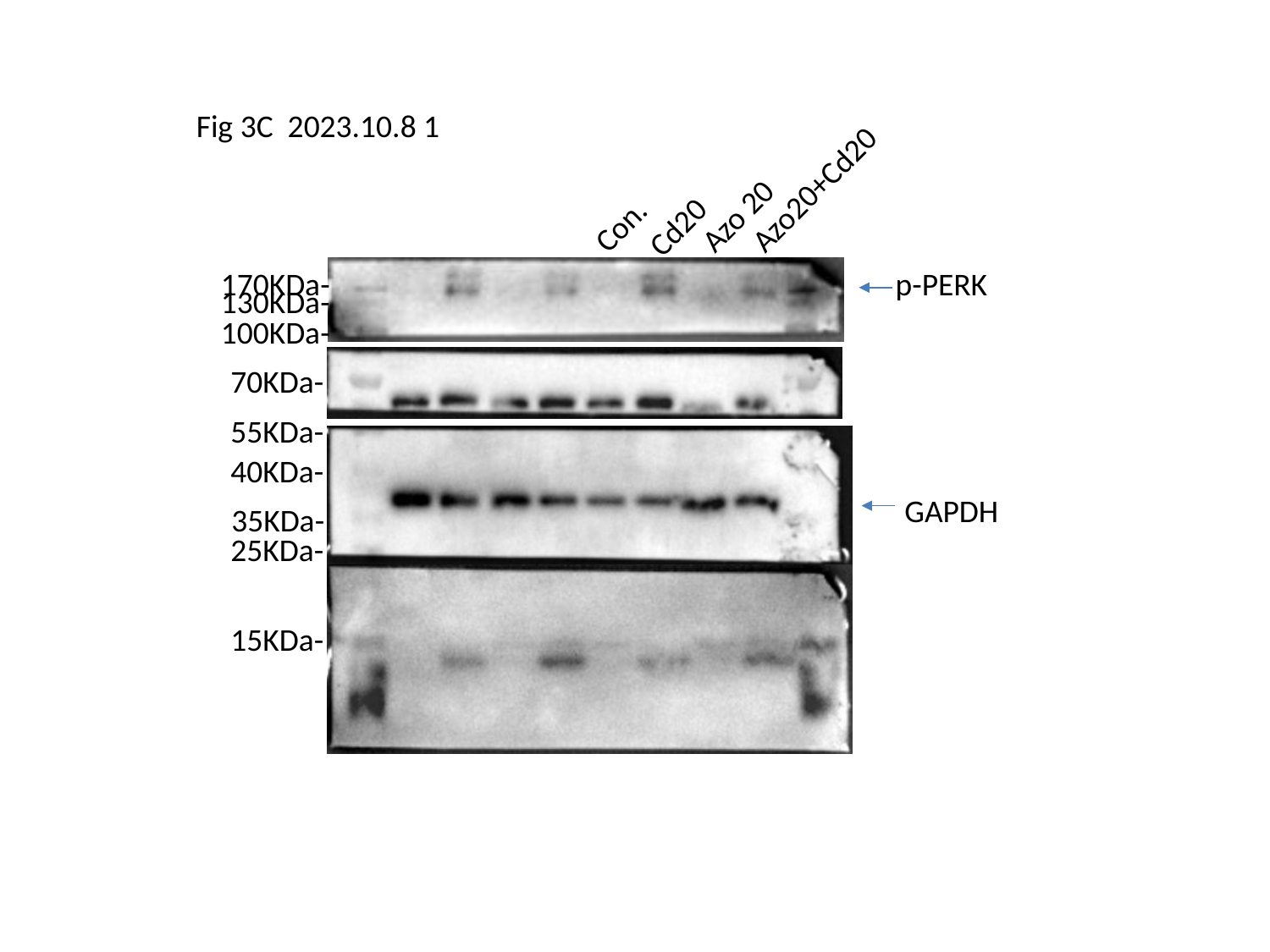

Fig 3C 2023.10.8 1
Azo20+Cd20
Cd20
Azo 20
Con.
170KDa-
p-PERK
130KDa-
100KDa-
70KDa-
55KDa-
40KDa-
GAPDH
35KDa-
25KDa-
15KDa-

## Slide 13
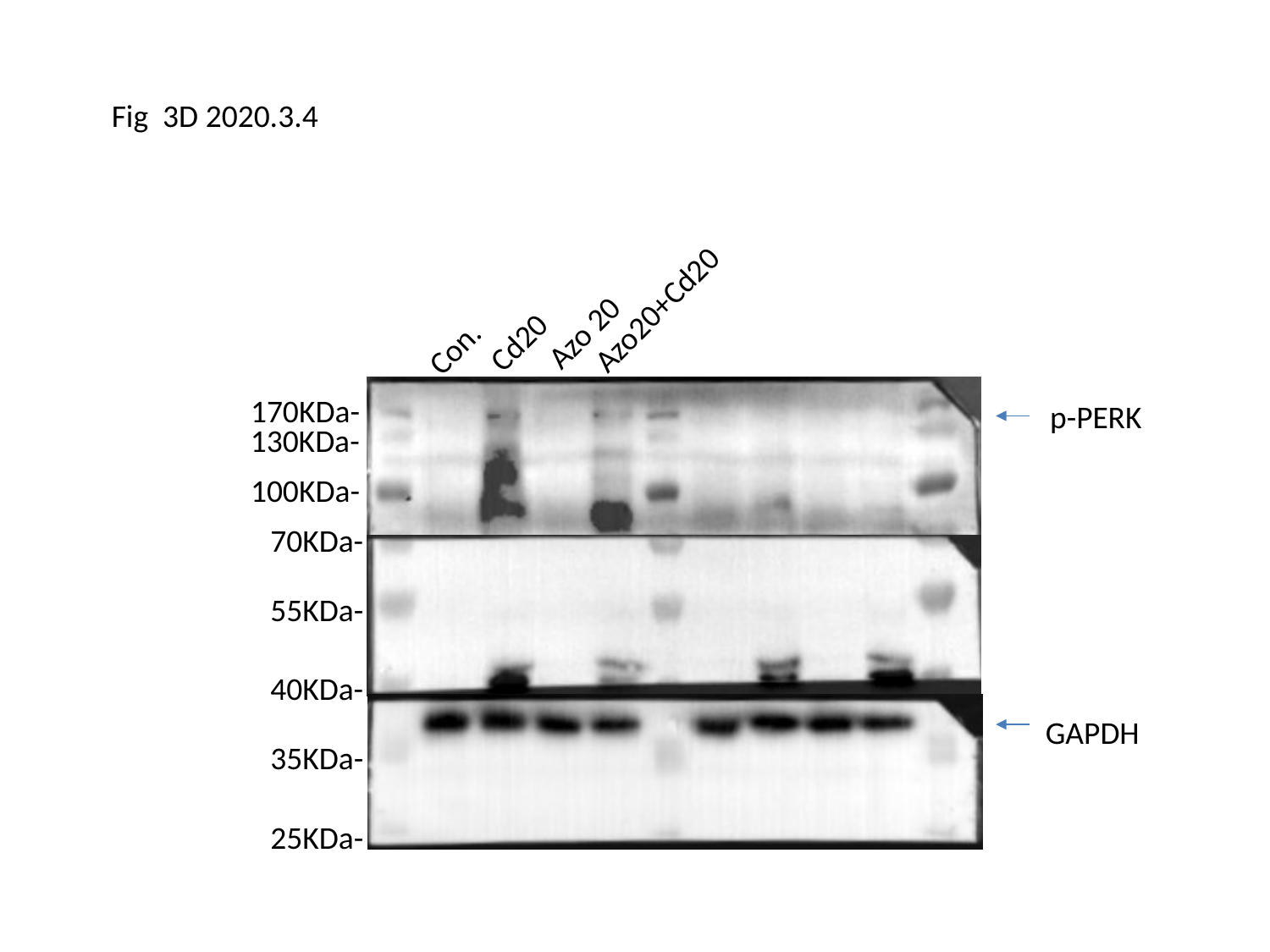

Fig 3D 2020.3.4
Azo20+Cd20
Azo 20
Con.
Cd20
170KDa-
130KDa-
100KDa-
70KDa-
55KDa-
40KDa-
35KDa-
25KDa-
p-PERK
GAPDH

## Slide 14
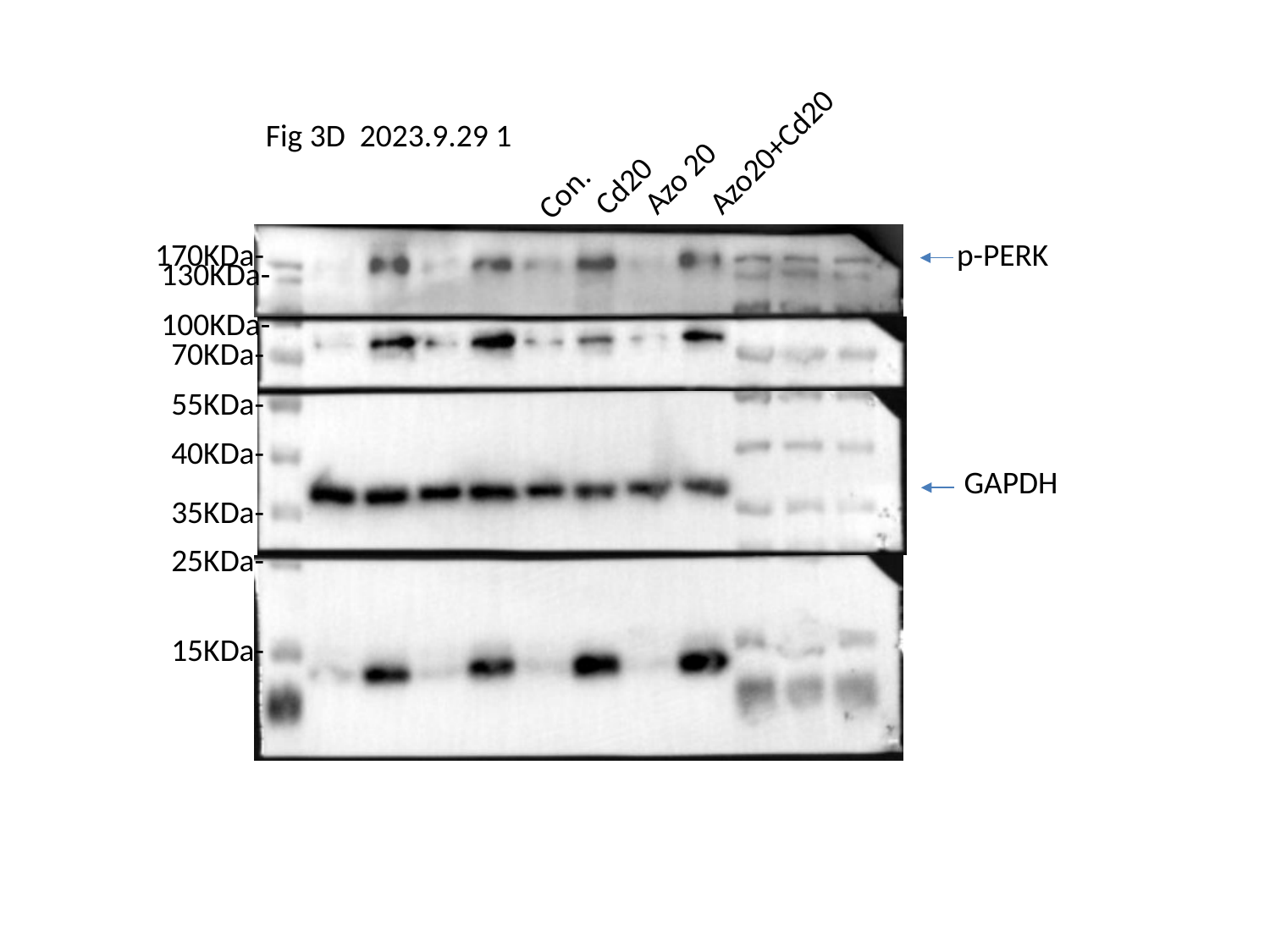

Fig 3D 2023.9.29 1
Azo20+Cd20
Cd20
Azo 20
Con.
170KDa-
p-PERK
130KDa-
100KDa-
70KDa-
55KDa-
40KDa-
GAPDH
35KDa-
25KDa-
15KDa-

## Slide 15
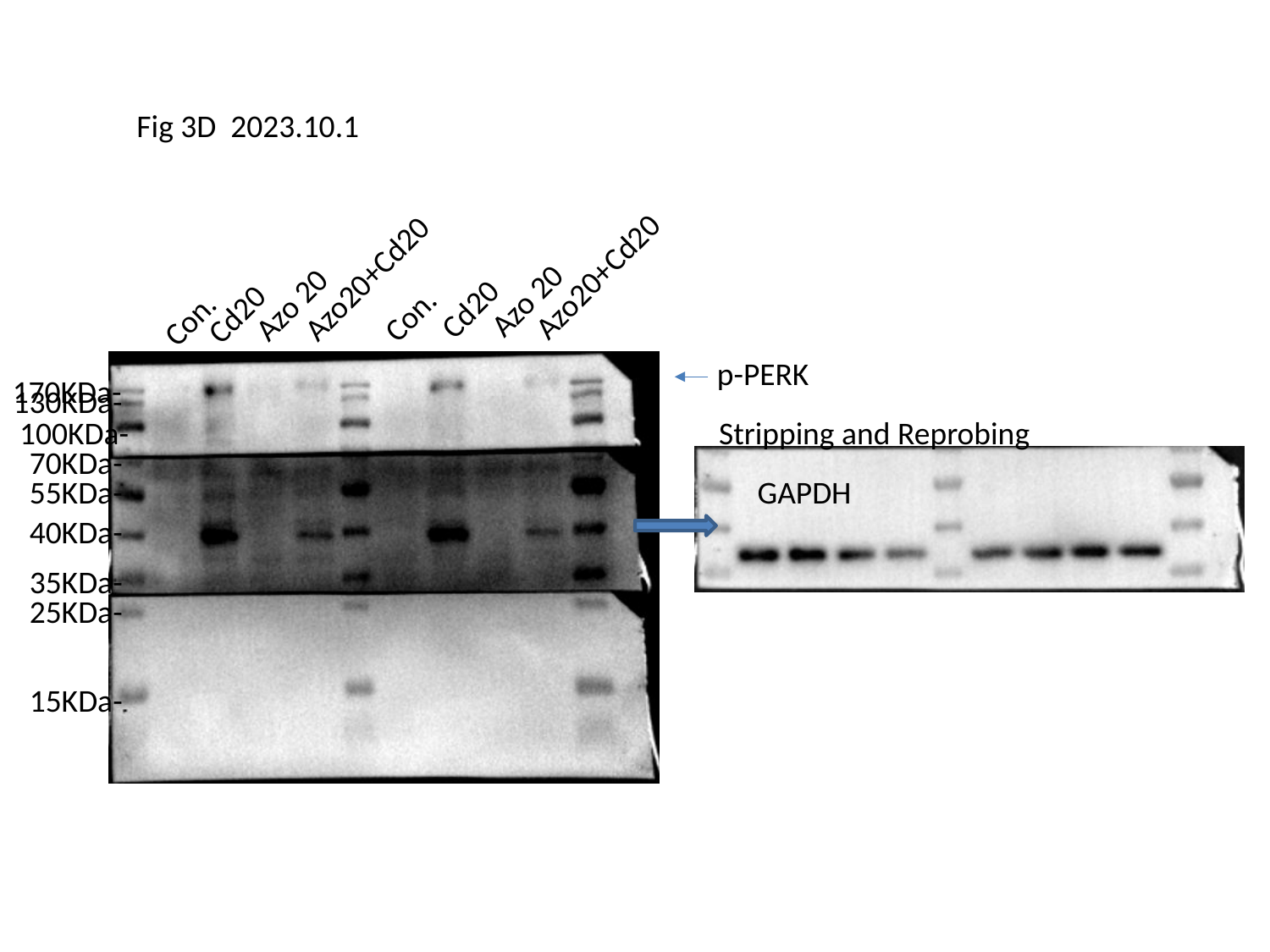

Fig 3D 2023.10.1
Azo20+Cd20
Azo20+Cd20
Cd20
Cd20
Azo 20
Azo 20
Con.
Con.
p-PERK
170KDa-
130KDa-
100KDa-
Stripping and Reprobing
70KDa-
55KDa-
GAPDH
40KDa-
35KDa-
25KDa-
15KDa-

## Slide 16
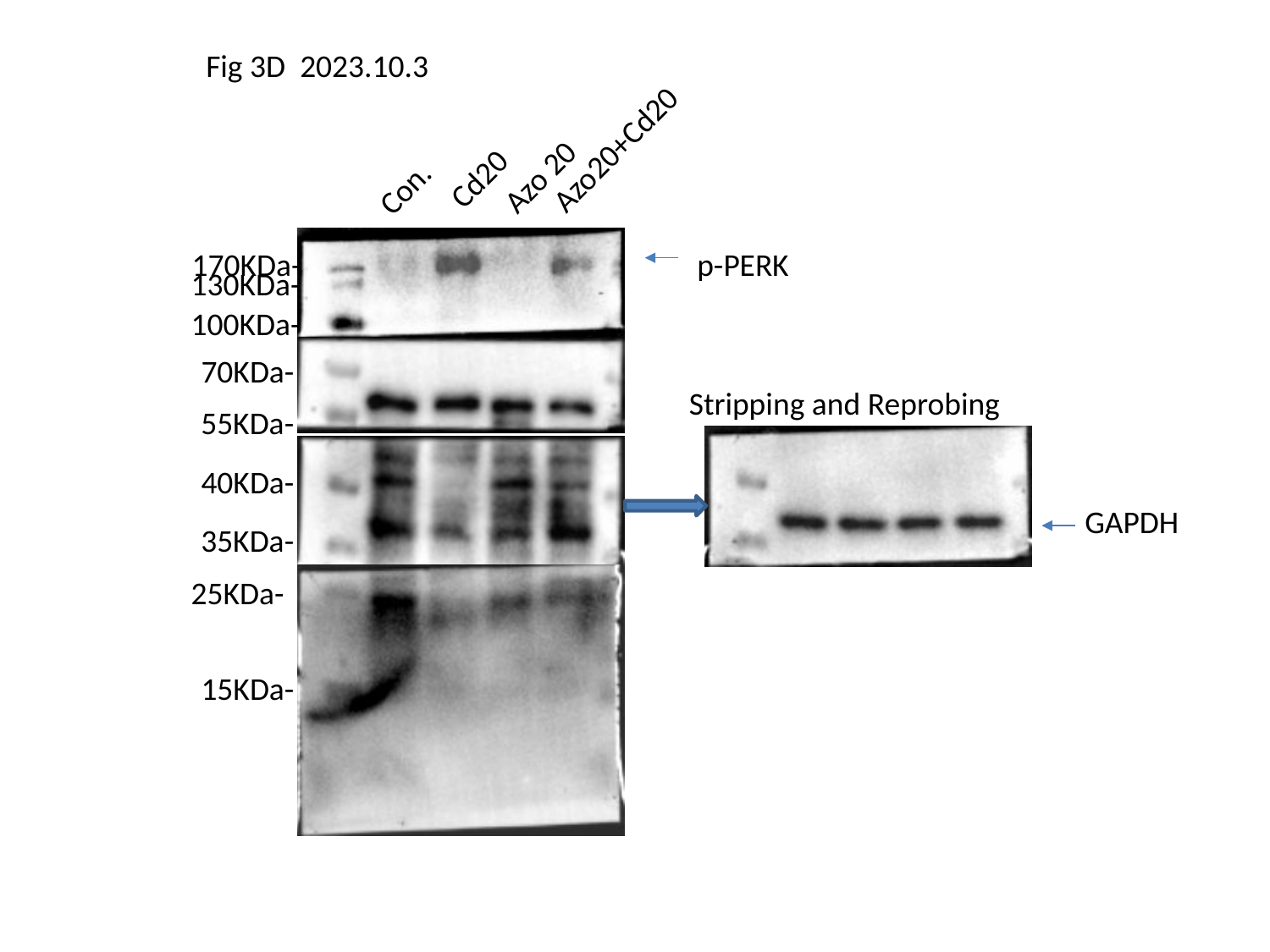

Fig 3D 2023.10.3
Azo20+Cd20
Cd20
Azo 20
Con.
170KDa-
p-PERK
130KDa-
100KDa-
70KDa-
Stripping and Reprobing
55KDa-
40KDa-
GAPDH
35KDa-
25KDa-
15KDa-

## Slide 17
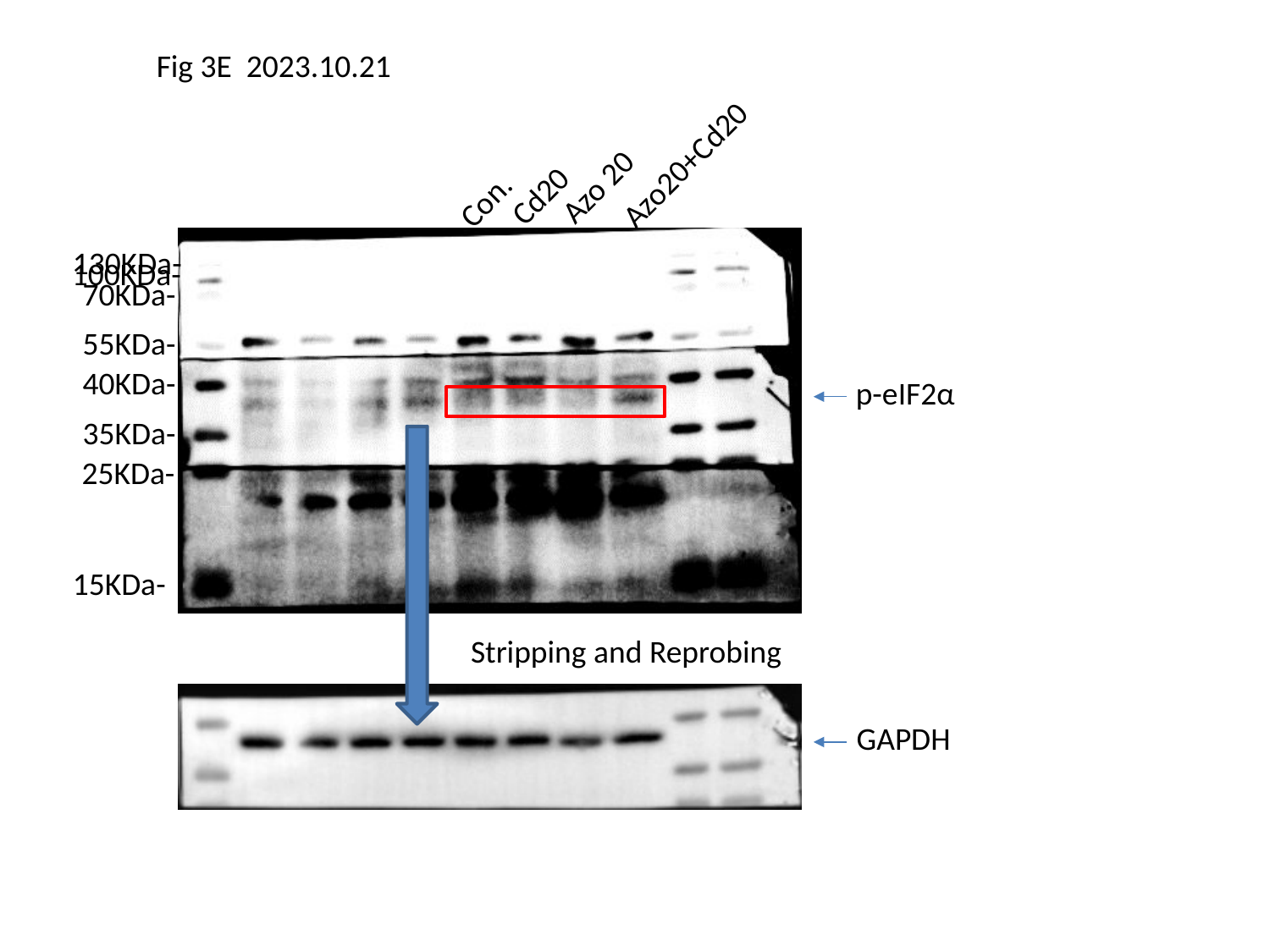

Fig 3E 2023.10.21
Azo20+Cd20
Cd20
Azo 20
Con.
130KDa-
100KDa-
70KDa-
55KDa-
40KDa-
p-eIF2α
35KDa-
25KDa-
15KDa-
Stripping and Reprobing
GAPDH

## Slide 18
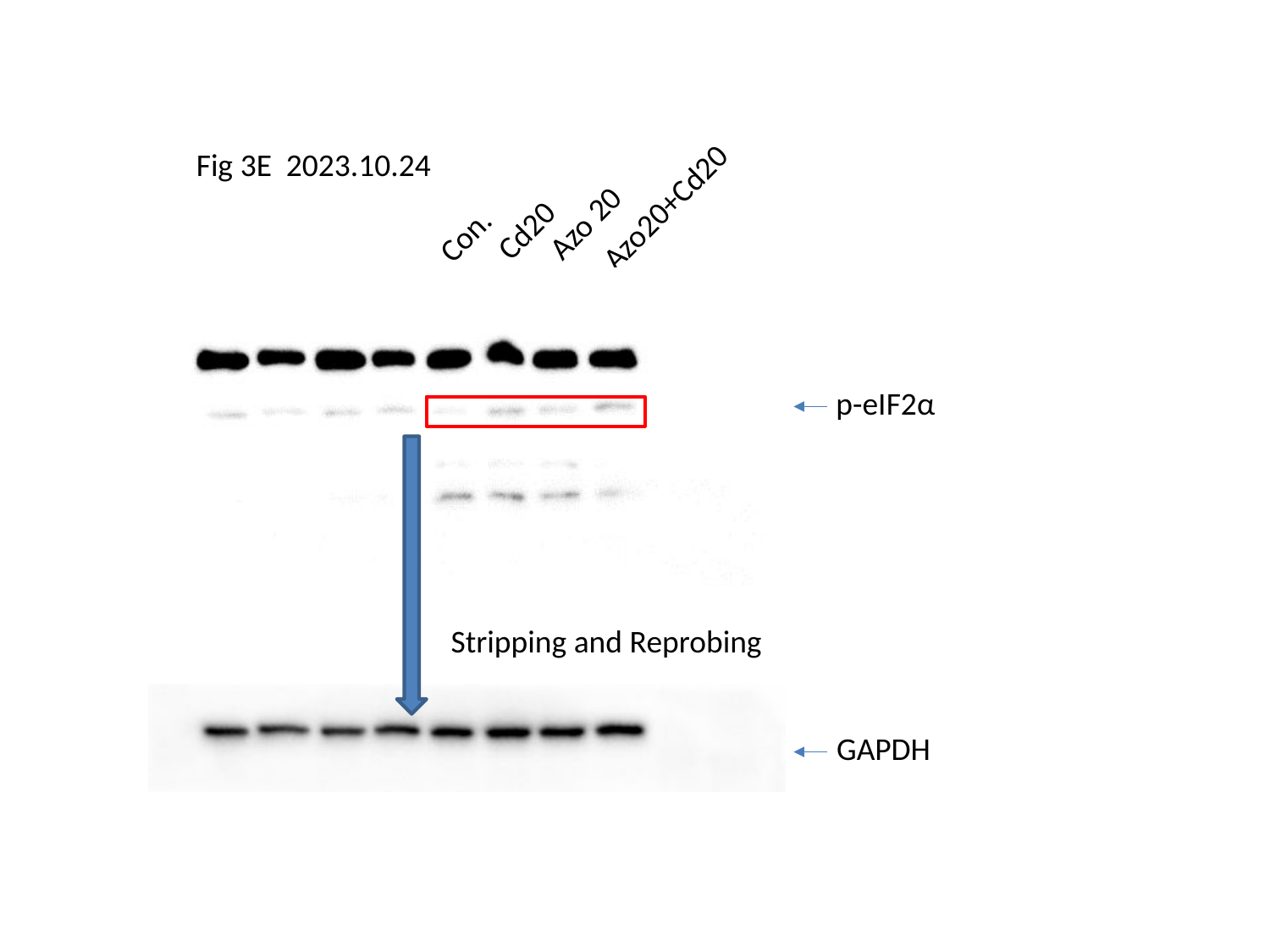

Fig 3E 2023.10.24
Azo20+Cd20
Cd20
Azo 20
Con.
p-eIF2α
Stripping and Reprobing
GAPDH

## Slide 19
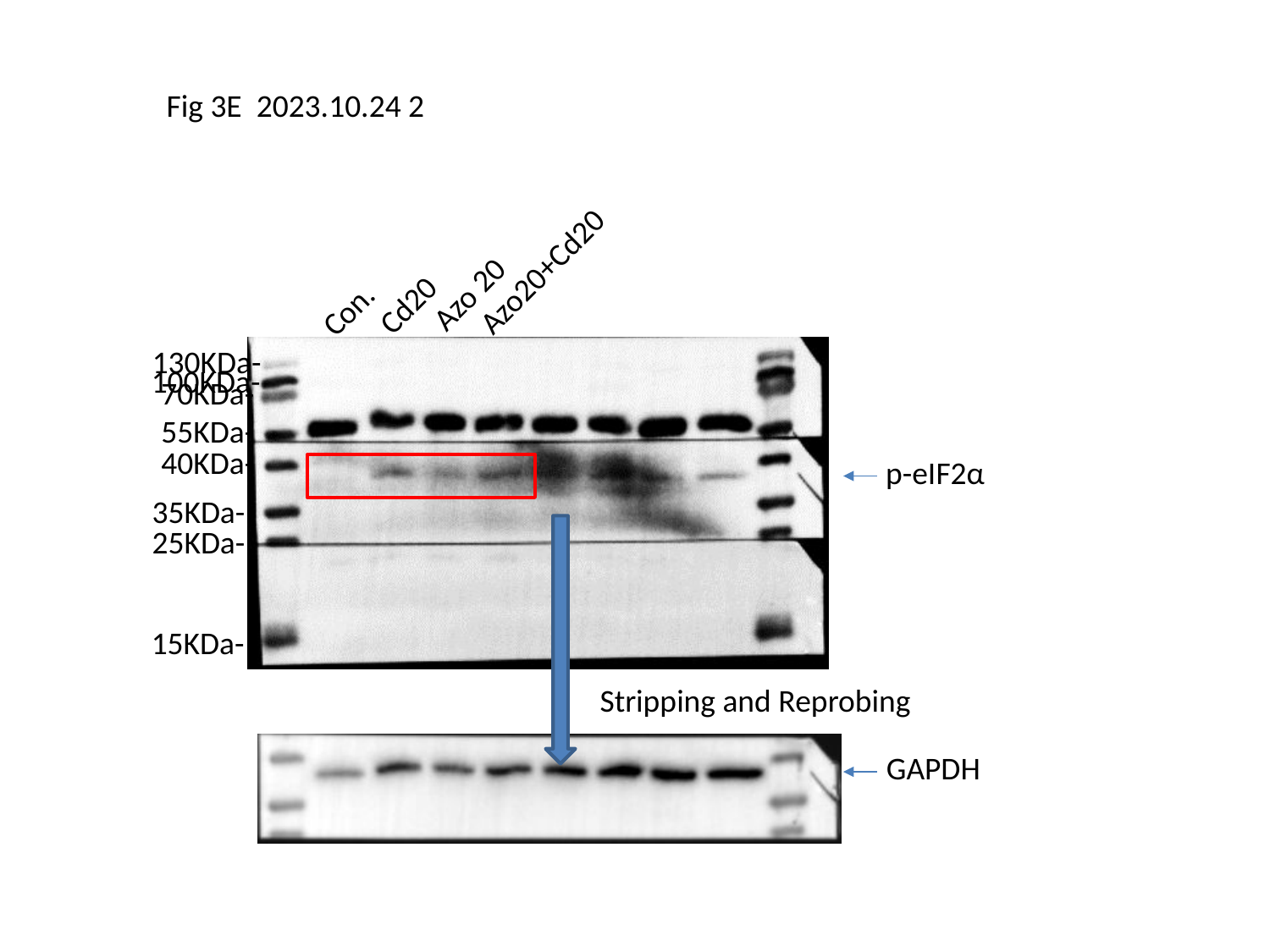

Fig 3E 2023.10.24 2
Azo20+Cd20
Cd20
Azo 20
Con.
130KDa-
100KDa-
70KDa-
55KDa-
40KDa-
p-eIF2α
35KDa-
25KDa-
15KDa-
Stripping and Reprobing
GAPDH

## Slide 20
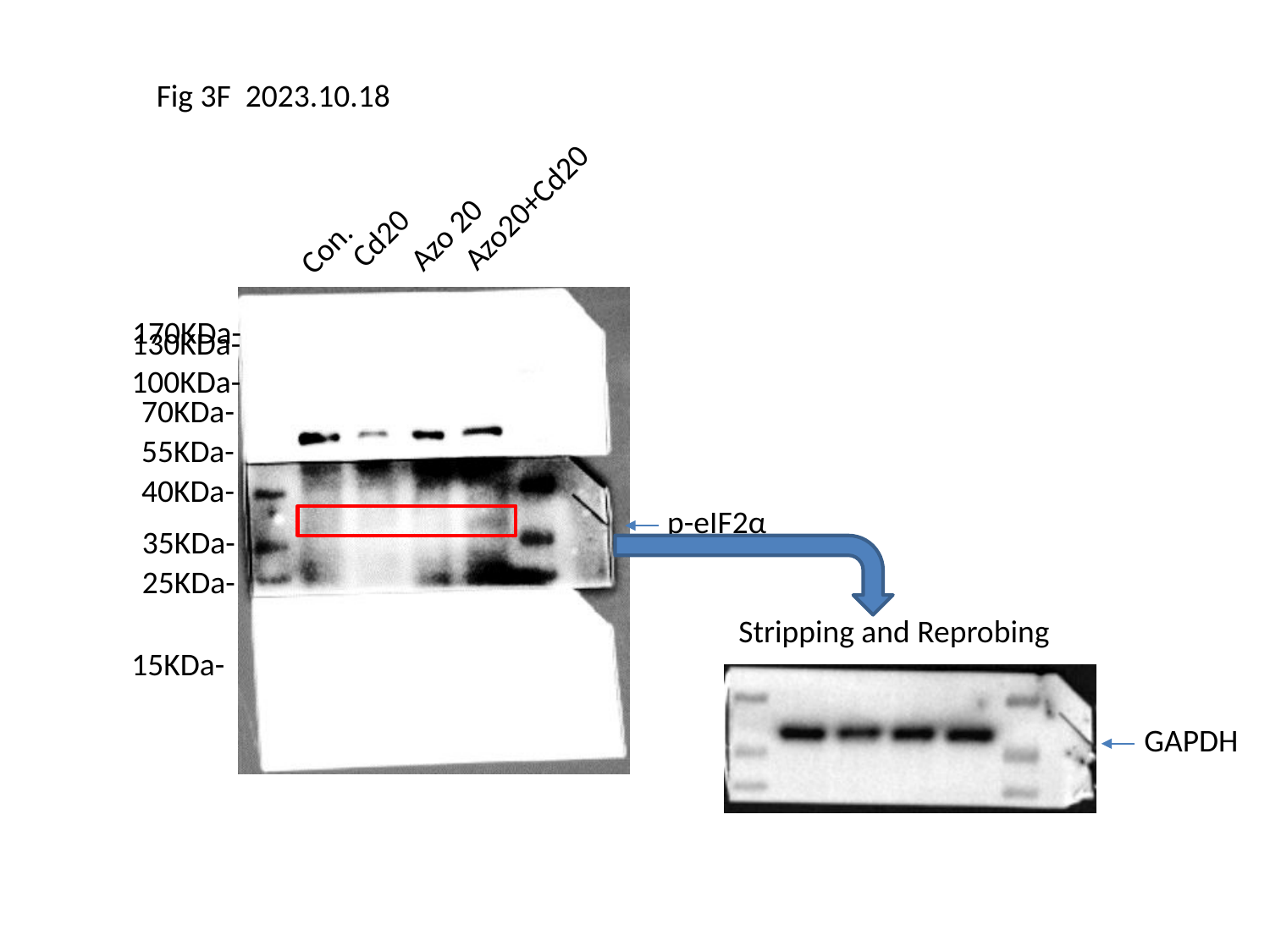

Fig 3F 2023.10.18
Azo20+Cd20
Cd20
Azo 20
Con.
170KDa-
130KDa-
100KDa-
70KDa-
55KDa-
40KDa-
p-eIF2α
35KDa-
25KDa-
Stripping and Reprobing
15KDa-
GAPDH

## Slide 21
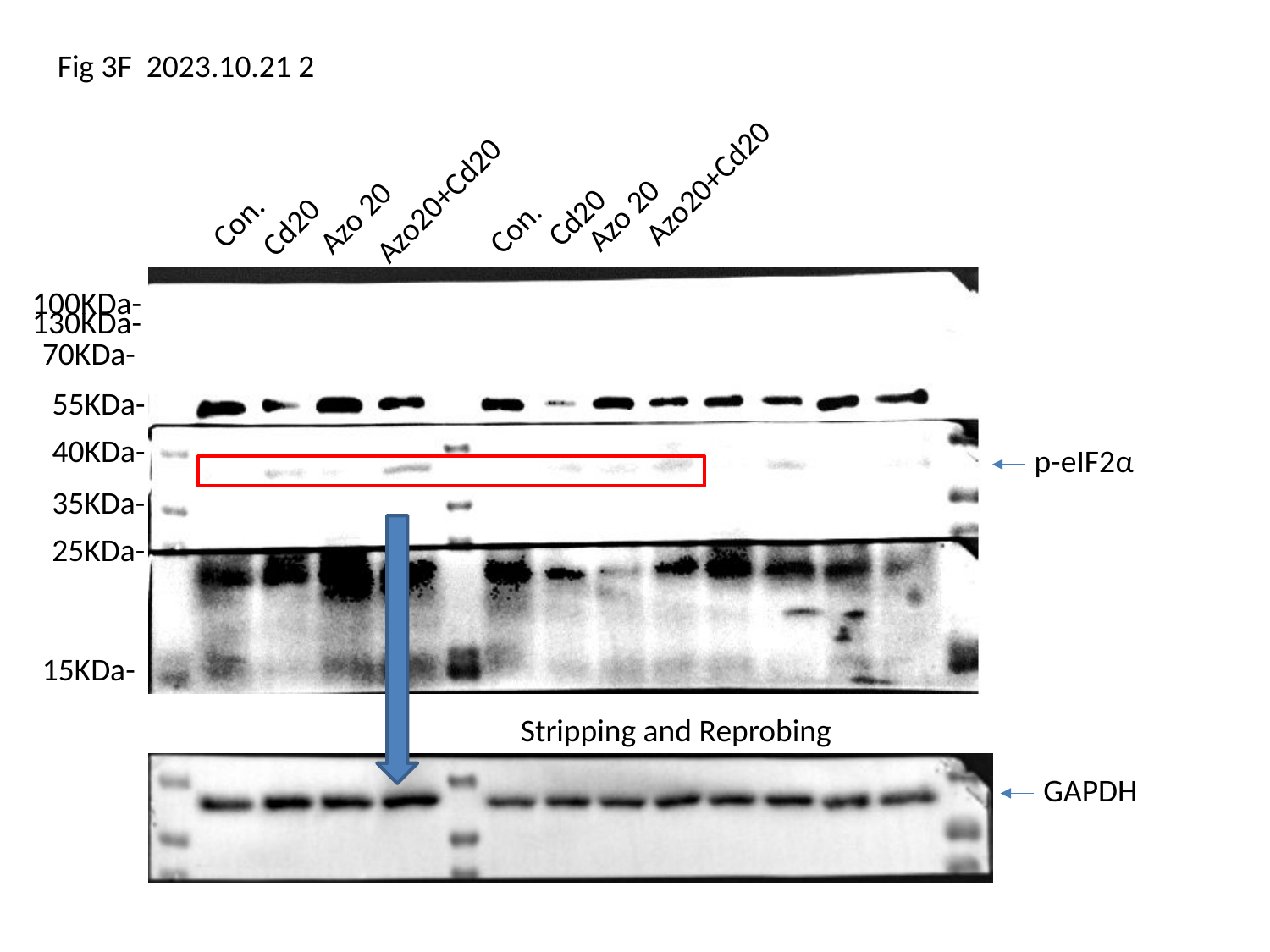

Fig 3F 2023.10.21 2
Azo20+Cd20
Cd20
Azo20+Cd20
Cd20
Azo 20
Azo 20
Con.
Con.
100KDa-
130KDa-
70KDa-
55KDa-
40KDa-
p-eIF2α
35KDa-
25KDa-
15KDa-
Stripping and Reprobing
GAPDH

## Slide 22
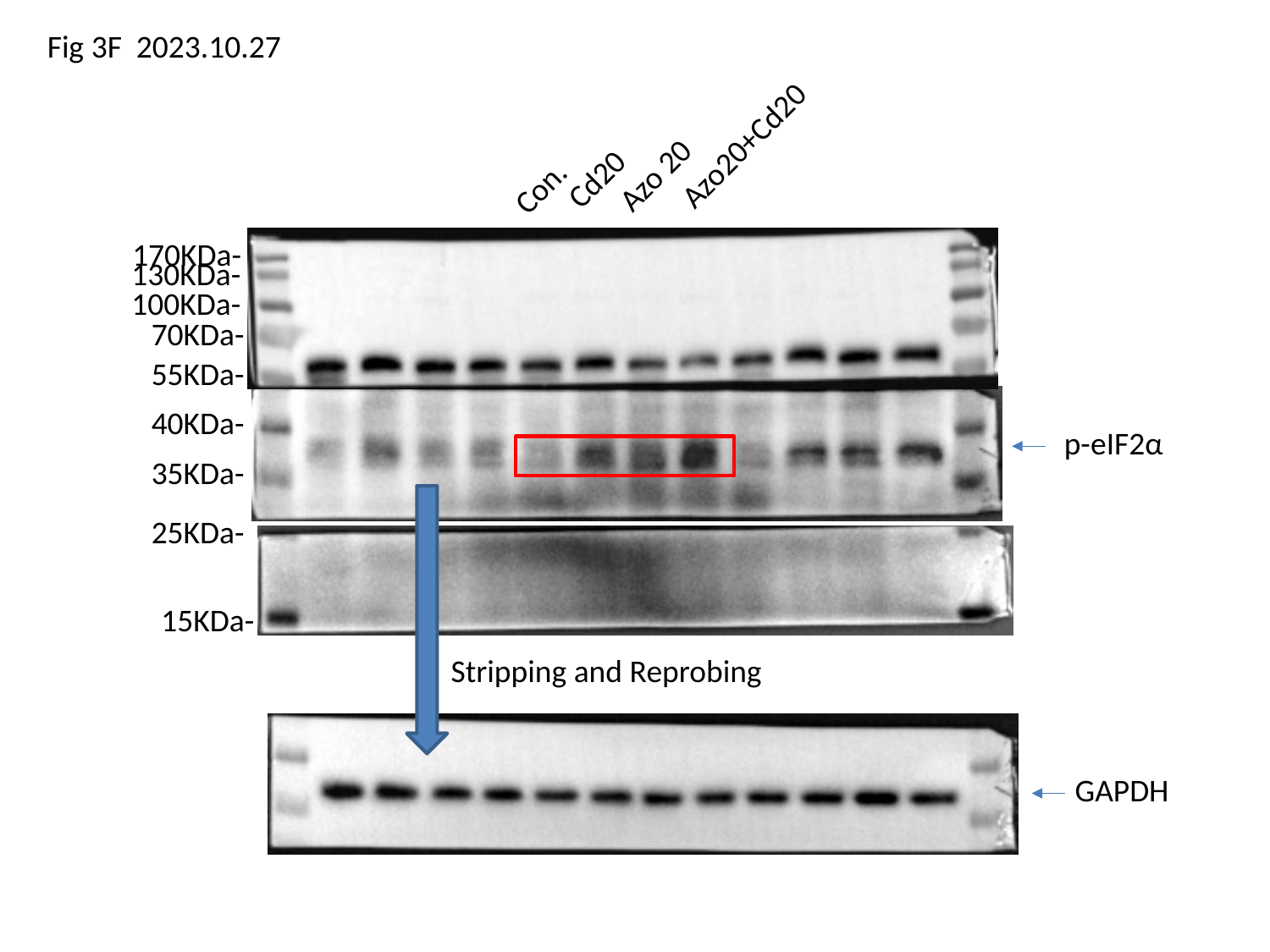

Fig 3F 2023.10.27
Azo20+Cd20
Cd20
Azo 20
Con.
170KDa-
130KDa-
100KDa-
70KDa-
55KDa-
40KDa-
p-eIF2α
35KDa-
25KDa-
15KDa-
Stripping and Reprobing
GAPDH

## Slide 23
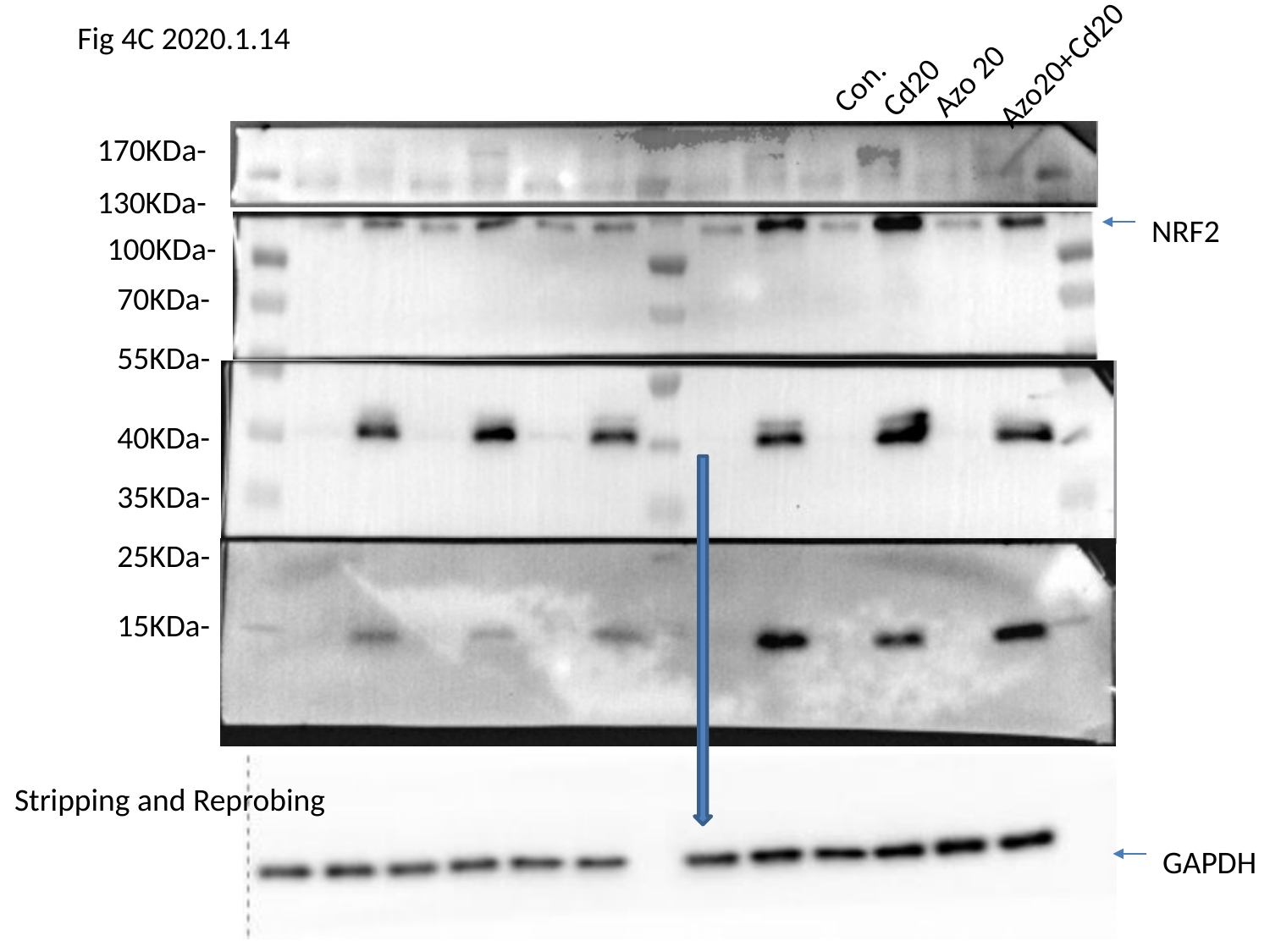

Fig 4C 2020.1.14
Azo20+Cd20
Azo 20
Con.
Cd20
170KDa-
130KDa-
100KDa-
70KDa-
55KDa-
40KDa-
35KDa-
25KDa-
15KDa-
NRF2
Stripping and Reprobing
GAPDH

## Slide 24
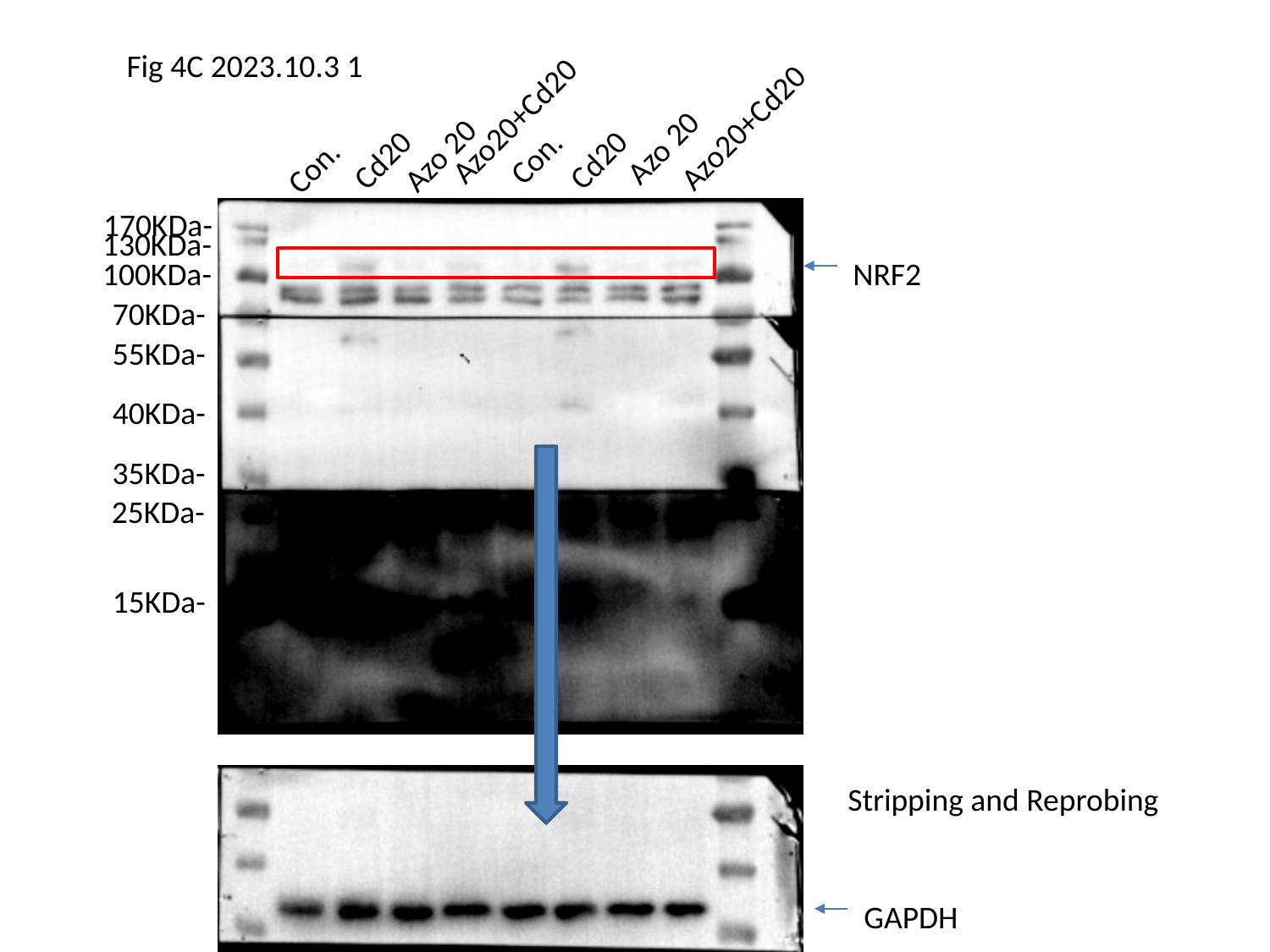

Fig 4C 2023.10.3 1
Azo20+Cd20
Azo20+Cd20
Cd20
Cd20
Azo 20
Con.
Azo 20
Con.
170KDa-
130KDa-
100KDa-
NRF2
70KDa-
55KDa-
40KDa-
35KDa-
25KDa-
15KDa-
Stripping and Reprobing
GAPDH

## Slide 25
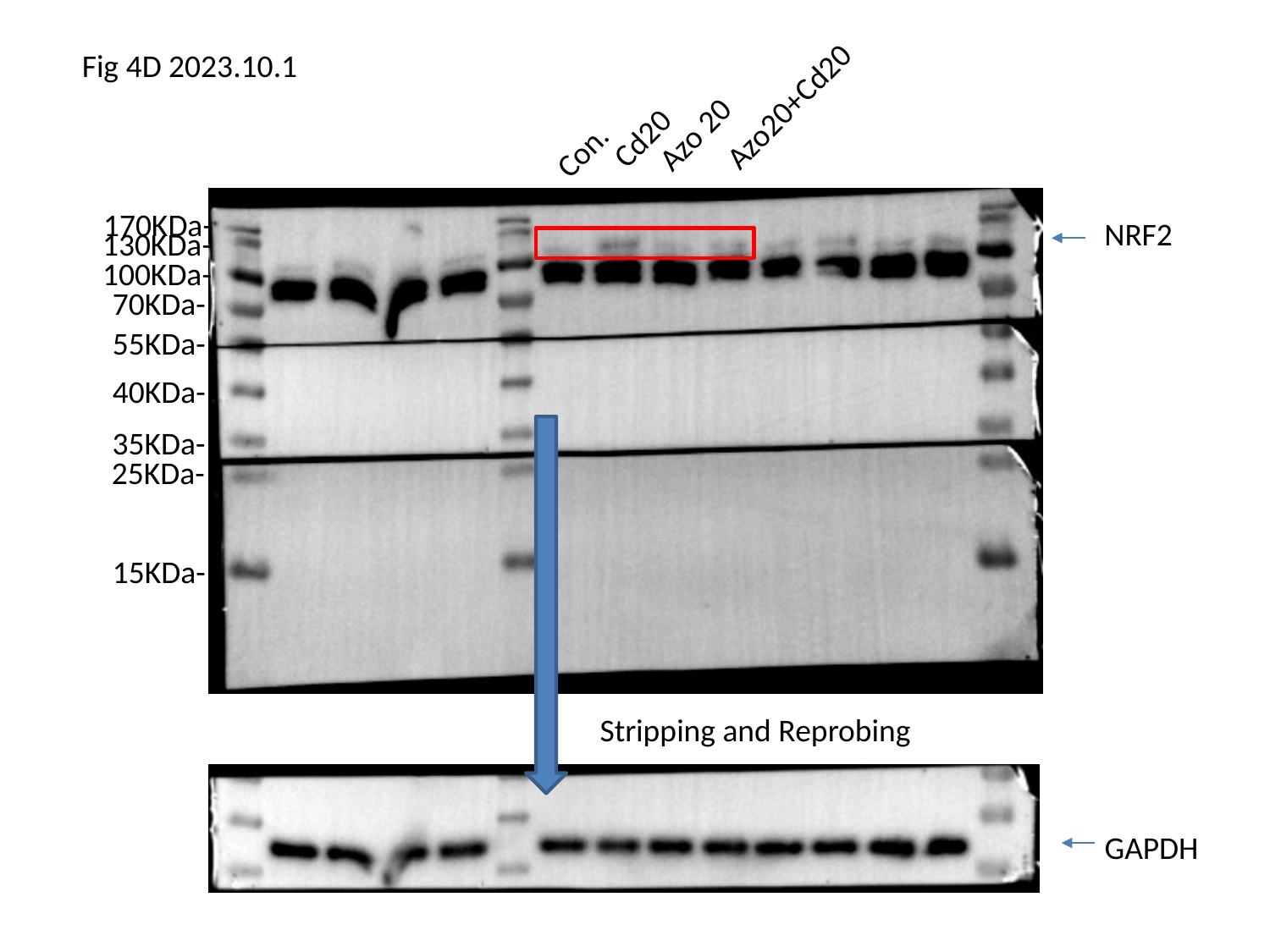

Fig 4D 2023.10.1
Azo20+Cd20
Cd20
Azo 20
Con.
170KDa-
NRF2
130KDa-
100KDa-
70KDa-
55KDa-
40KDa-
35KDa-
25KDa-
15KDa-
Stripping and Reprobing
GAPDH

## Slide 26
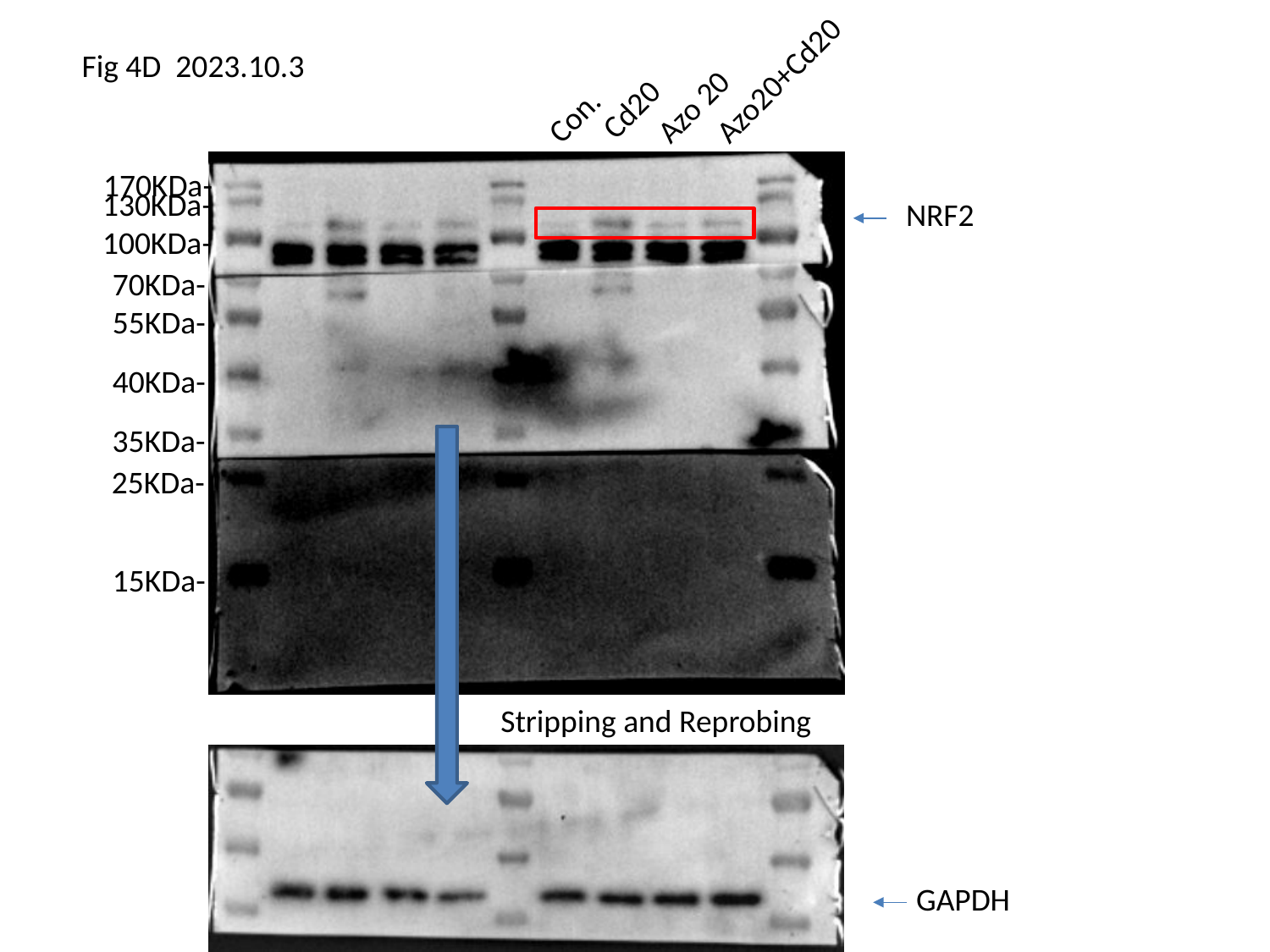

Fig 4D 2023.10.3
Azo20+Cd20
Cd20
Azo 20
Con.
170KDa-
130KDa-
NRF2
100KDa-
70KDa-
55KDa-
40KDa-
35KDa-
25KDa-
15KDa-
Stripping and Reprobing
GAPDH

## Slide 27
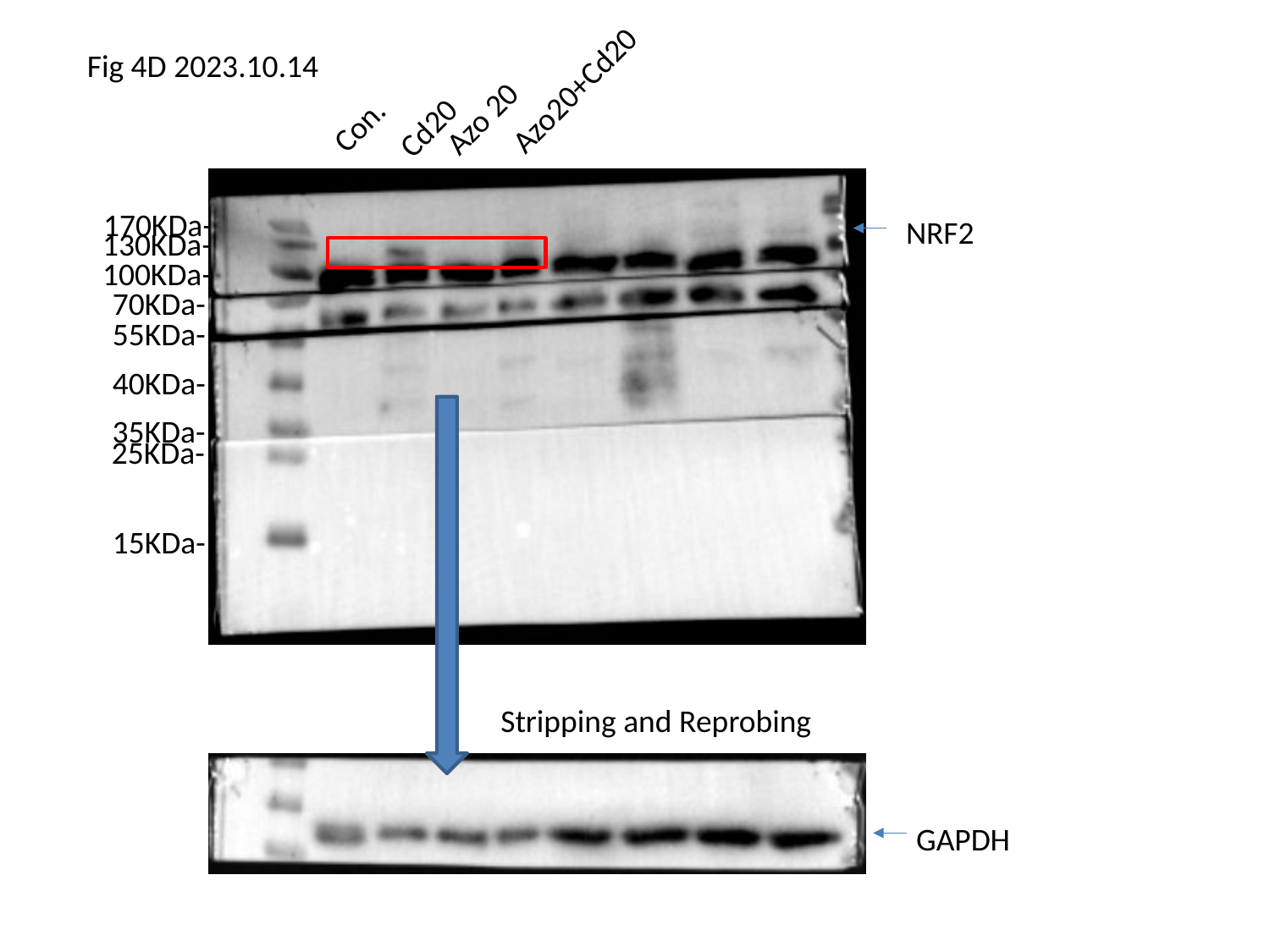

Fig 4D 2023.10.14
Azo20+Cd20
Cd20
Azo 20
Con.
170KDa-
NRF2
130KDa-
100KDa-
70KDa-
55KDa-
40KDa-
35KDa-
25KDa-
15KDa-
Stripping and Reprobing
GAPDH

## Slide 28
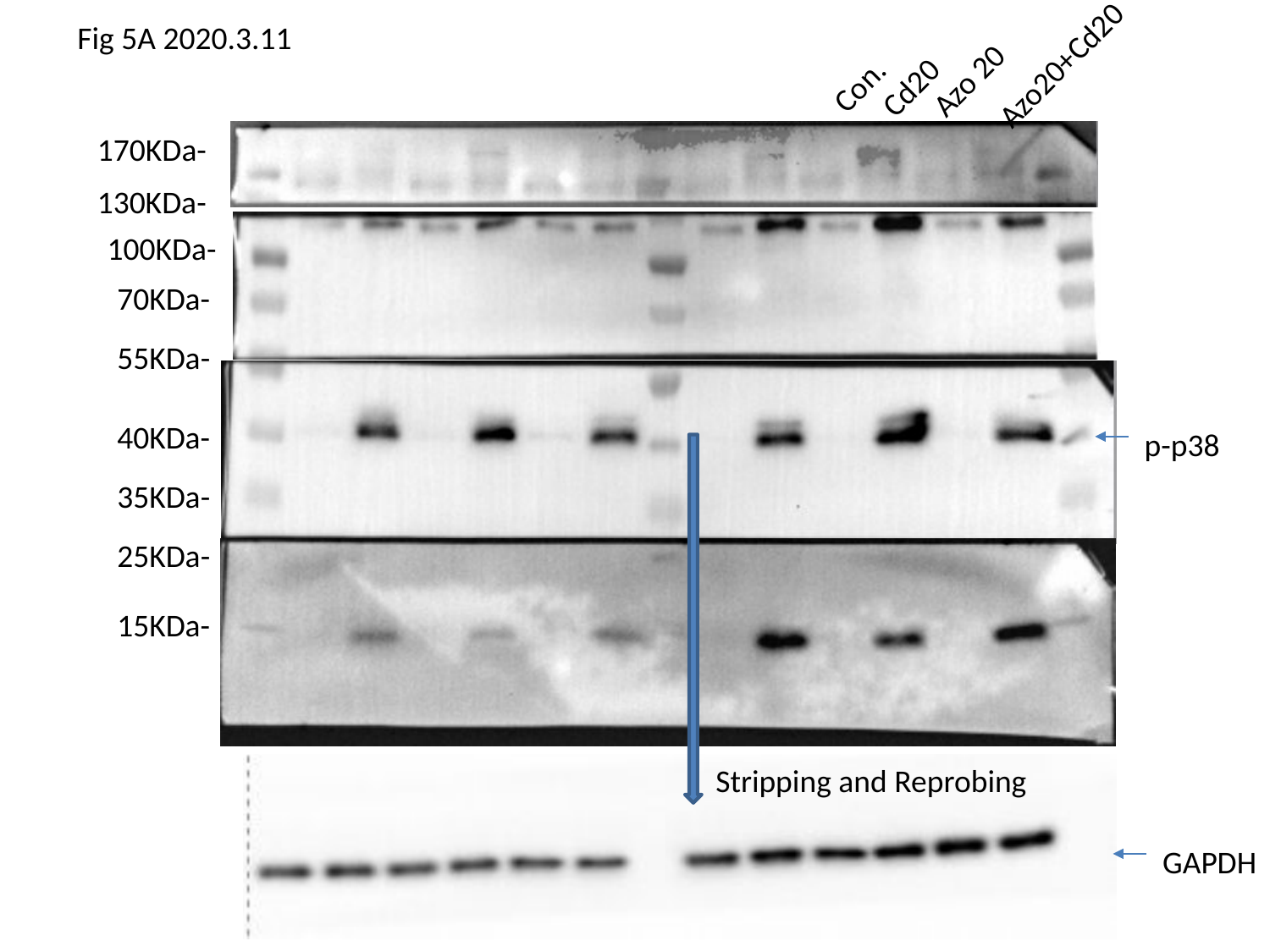

Fig 5A 2020.3.11
Azo20+Cd20
Azo 20
Con.
Cd20
170KDa-
130KDa-
100KDa-
70KDa-
55KDa-
40KDa-
35KDa-
25KDa-
15KDa-
p-p38
Stripping and Reprobing
GAPDH

## Slide 29
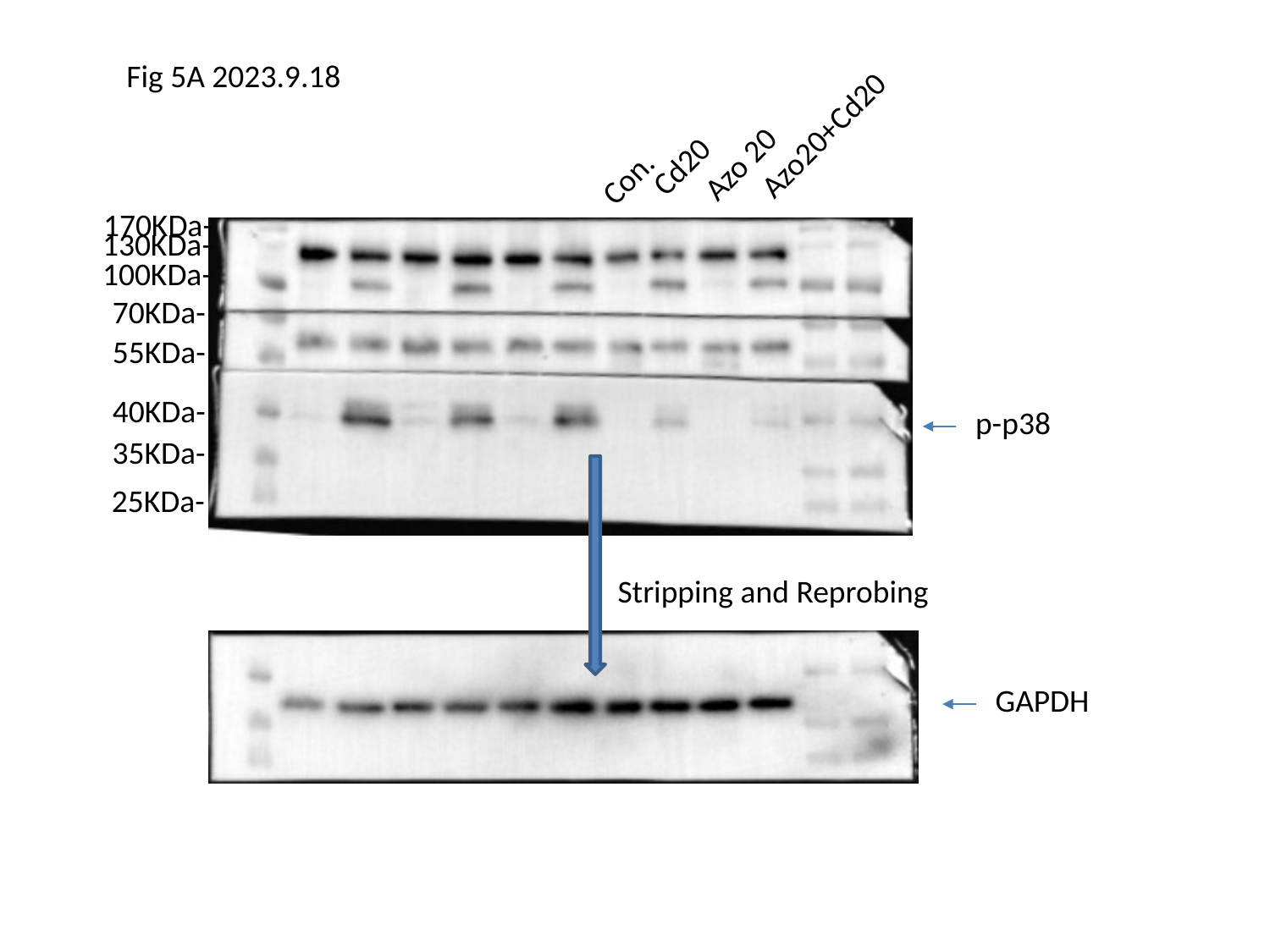

Fig 5A 2023.9.18
Azo20+Cd20
Cd20
Azo 20
Con.
170KDa-
130KDa-
100KDa-
70KDa-
55KDa-
40KDa-
p-p38
35KDa-
25KDa-
Stripping and Reprobing
GAPDH

## Slide 30
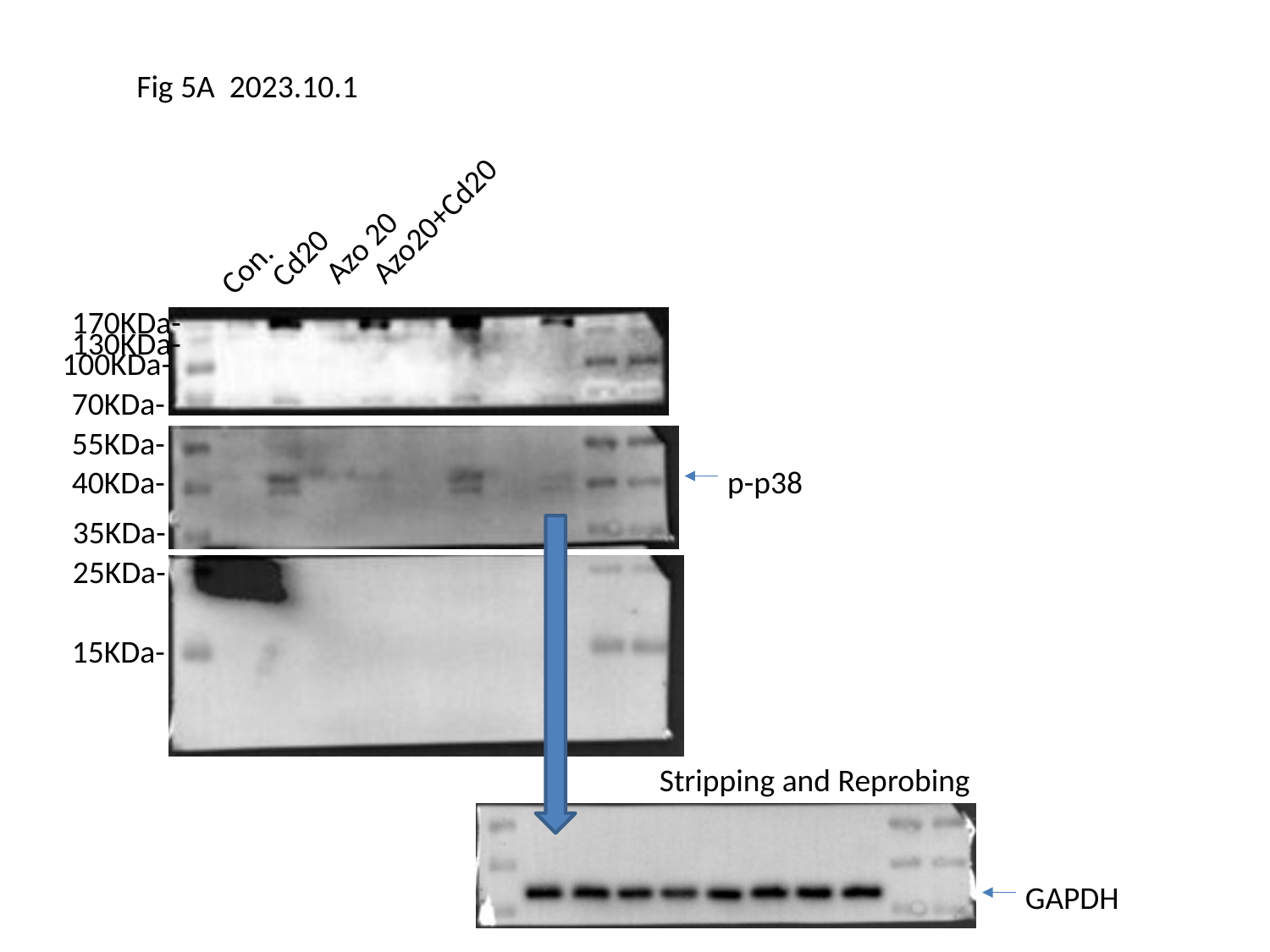

Fig 5A 2023.10.1
Azo20+Cd20
Cd20
Azo 20
Con.
170KDa-
130KDa-
100KDa-
70KDa-
55KDa-
40KDa-
p-p38
35KDa-
25KDa-
15KDa-
Stripping and Reprobing
GAPDH

## Slide 31
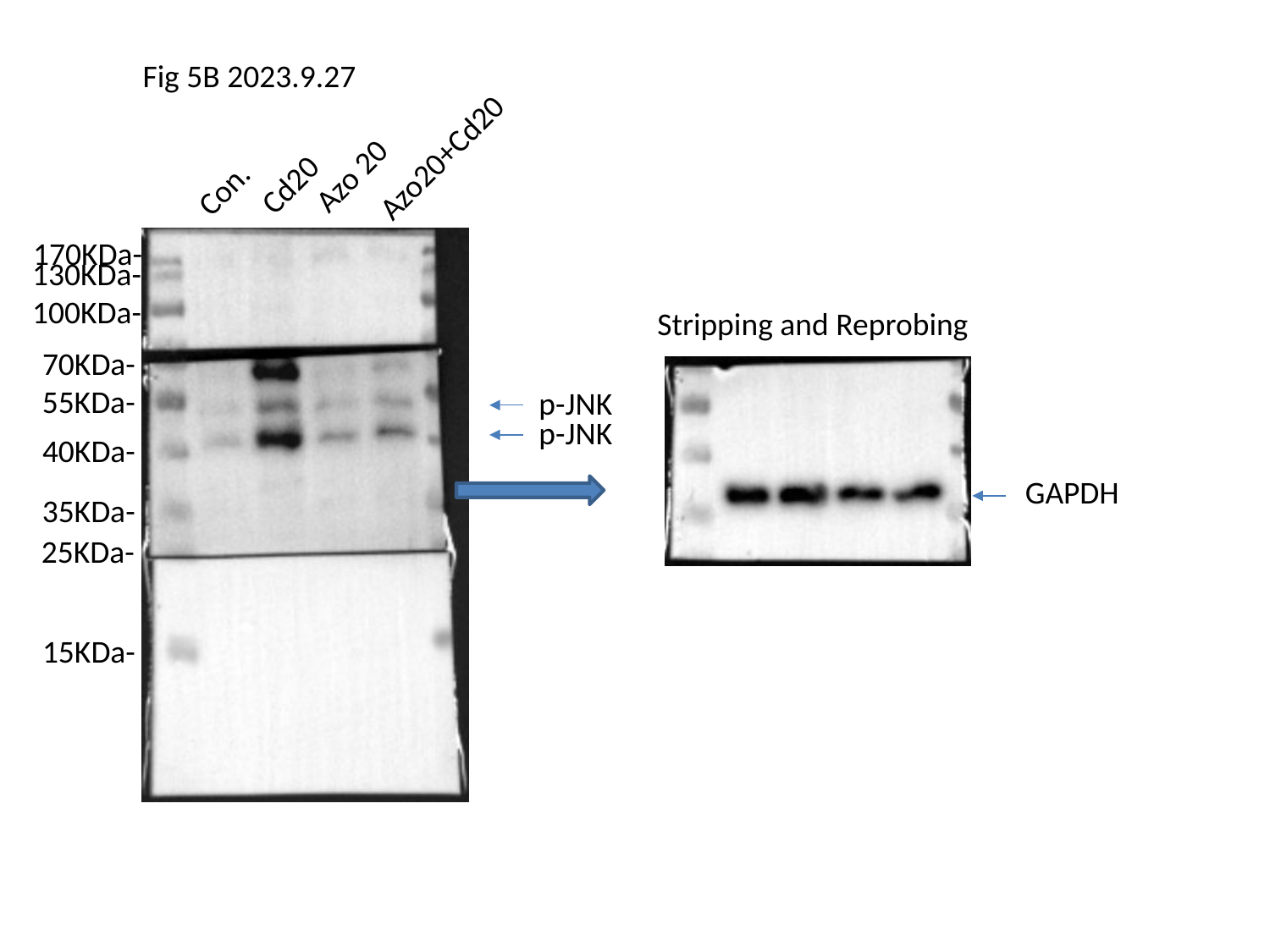

Fig 5B 2023.9.27
Azo20+Cd20
Cd20
Azo 20
Con.
170KDa-
130KDa-
100KDa-
Stripping and Reprobing
70KDa-
55KDa-
p-JNK
p-JNK
40KDa-
GAPDH
35KDa-
25KDa-
15KDa-

## Slide 32
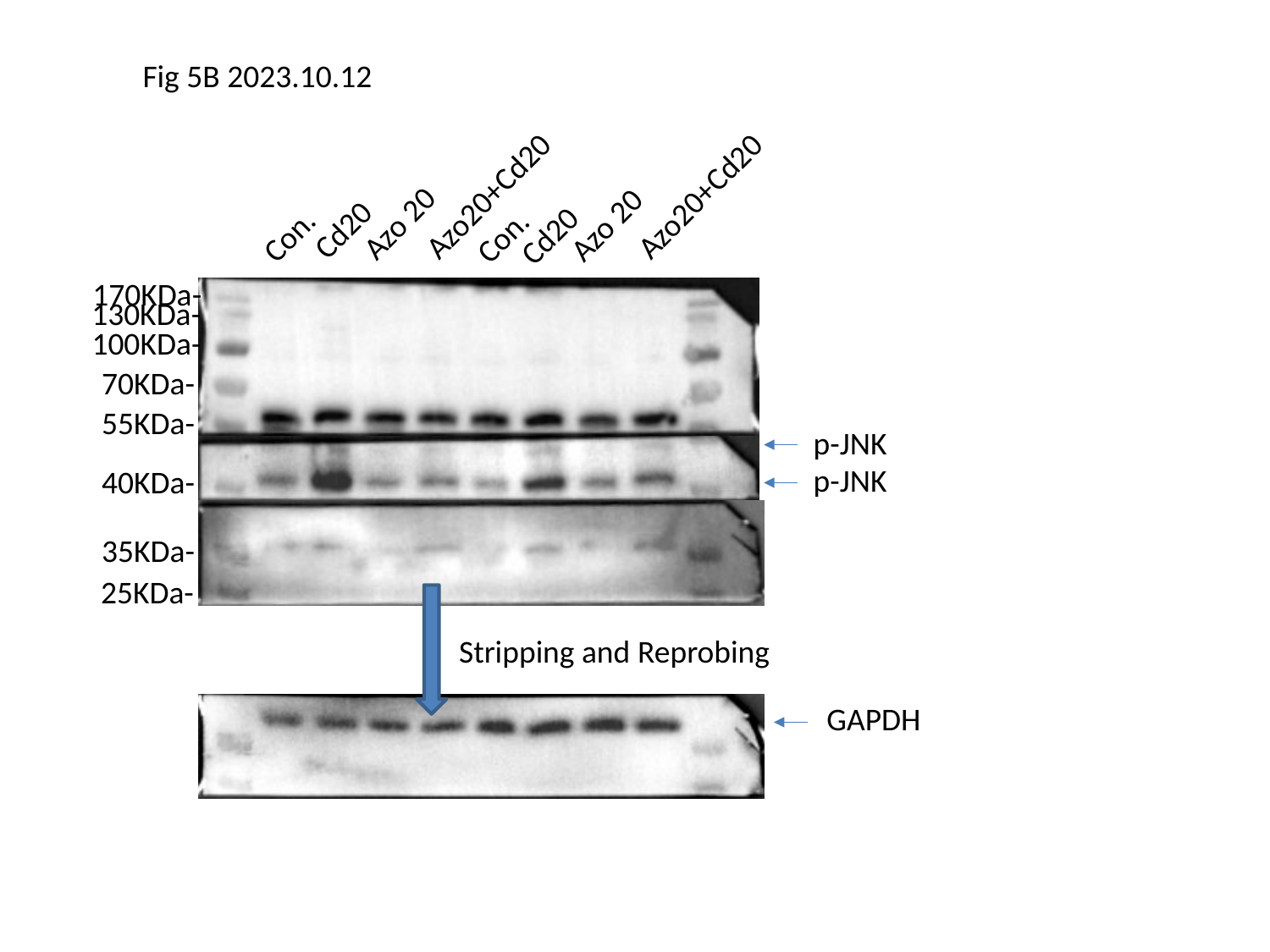

Fig 5B 2023.10.12
Azo20+Cd20
Azo20+Cd20
Cd20
Cd20
Azo 20
Azo 20
Con.
Con.
170KDa-
130KDa-
100KDa-
70KDa-
55KDa-
p-JNK
p-JNK
40KDa-
35KDa-
25KDa-
Stripping and Reprobing
GAPDH

## Slide 33
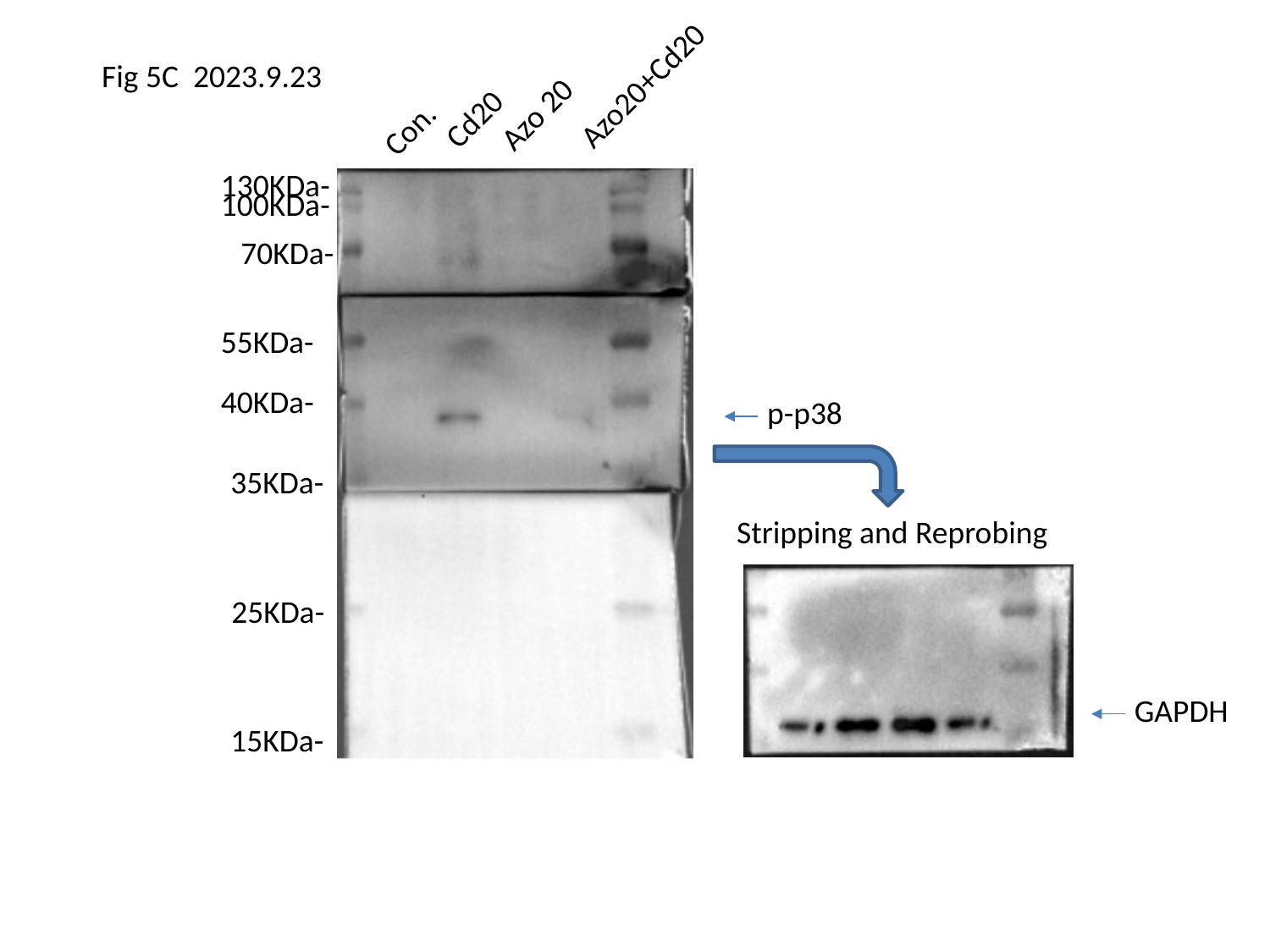

Fig 5C 2023.9.23
Azo20+Cd20
Cd20
Azo 20
Con.
130KDa-
100KDa-
70KDa-
55KDa-
40KDa-
p-p38
35KDa-
Stripping and Reprobing
25KDa-
GAPDH
15KDa-

## Slide 34
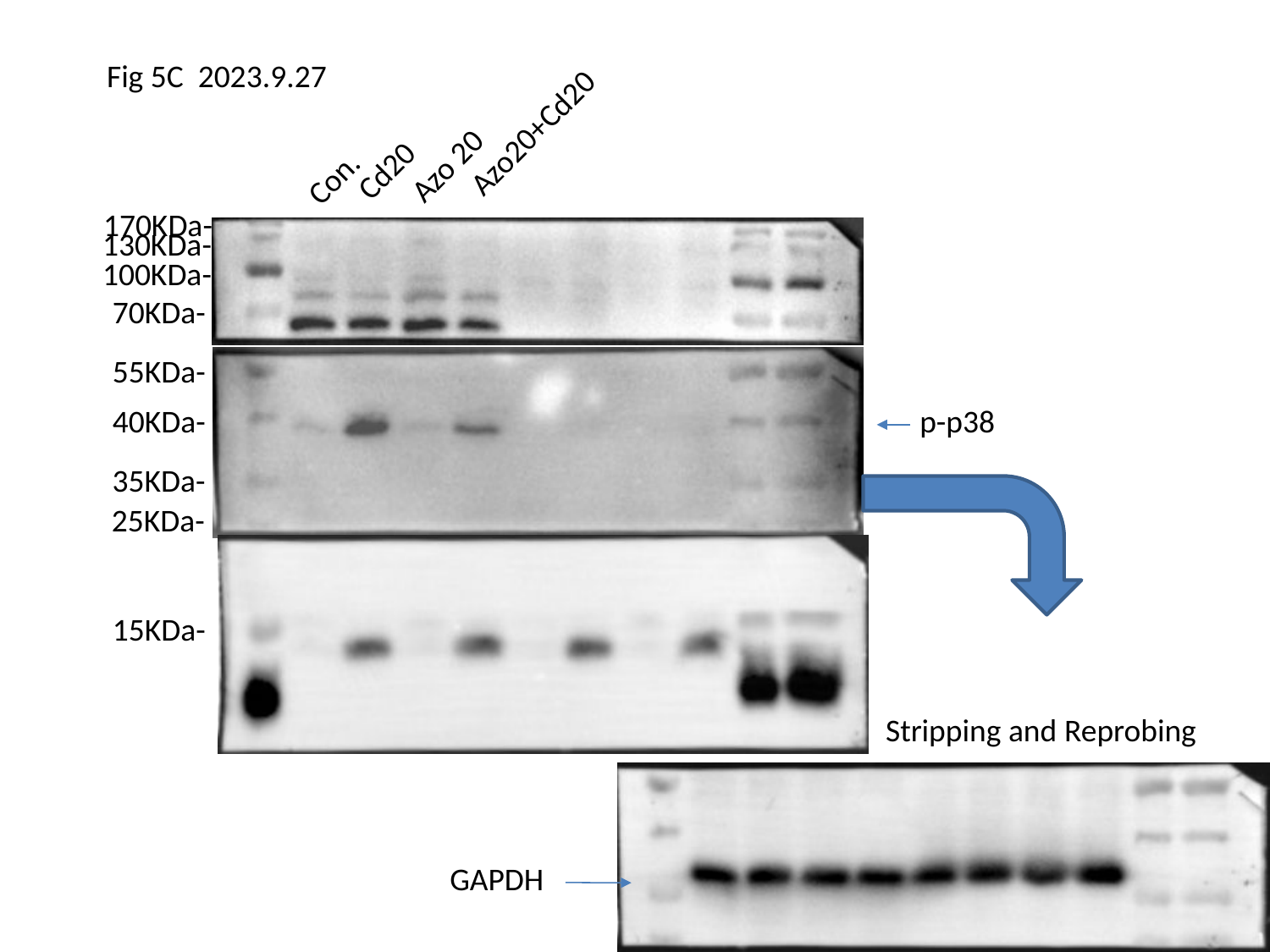

Fig 5C 2023.9.27
Azo20+Cd20
Cd20
Azo 20
Con.
170KDa-
130KDa-
100KDa-
70KDa-
55KDa-
40KDa-
p-p38
35KDa-
25KDa-
15KDa-
Stripping and Reprobing
GAPDH

## Slide 35
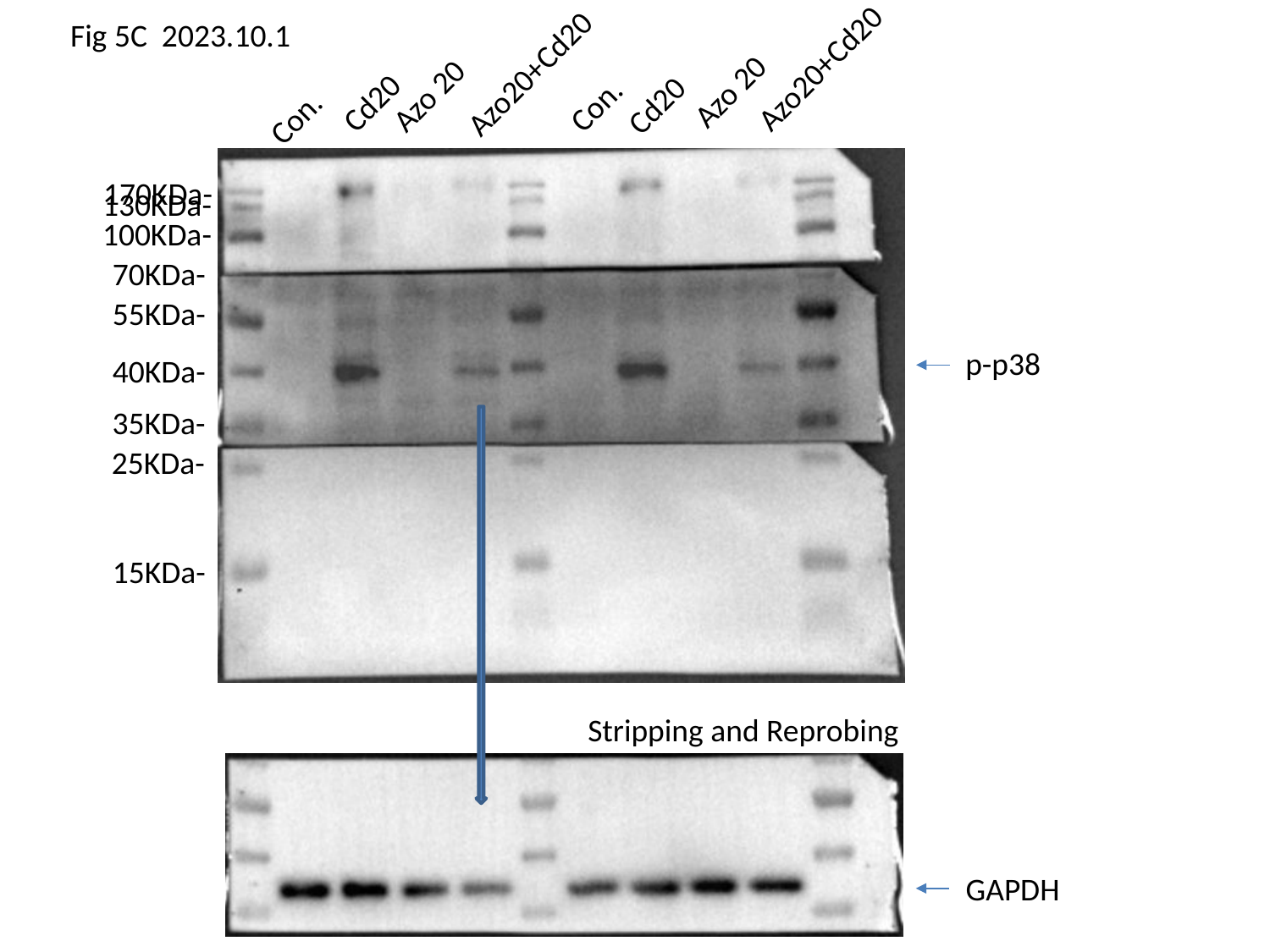

Fig 5C 2023.10.1
Azo20+Cd20
Azo20+Cd20
Cd20
Cd20
Azo 20
Azo 20
Con.
Con.
170KDa-
130KDa-
100KDa-
70KDa-
55KDa-
p-p38
40KDa-
35KDa-
25KDa-
15KDa-
Stripping and Reprobing
GAPDH

## Slide 36
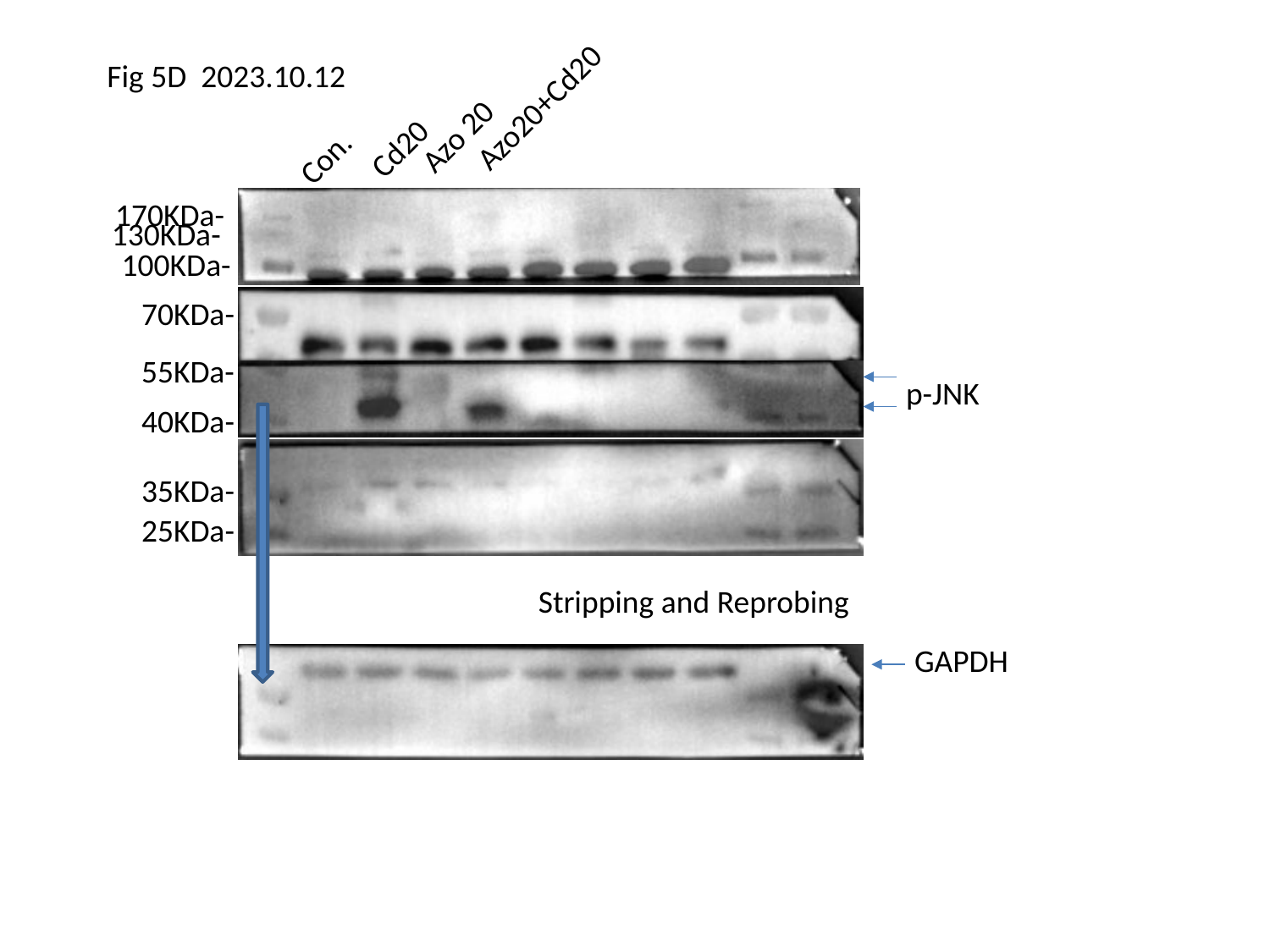

Fig 5D 2023.10.12
Azo20+Cd20
Cd20
Azo 20
Con.
170KDa-
130KDa-
100KDa-
70KDa-
55KDa-
p-JNK
40KDa-
35KDa-
25KDa-
Stripping and Reprobing
GAPDH

## Slide 37
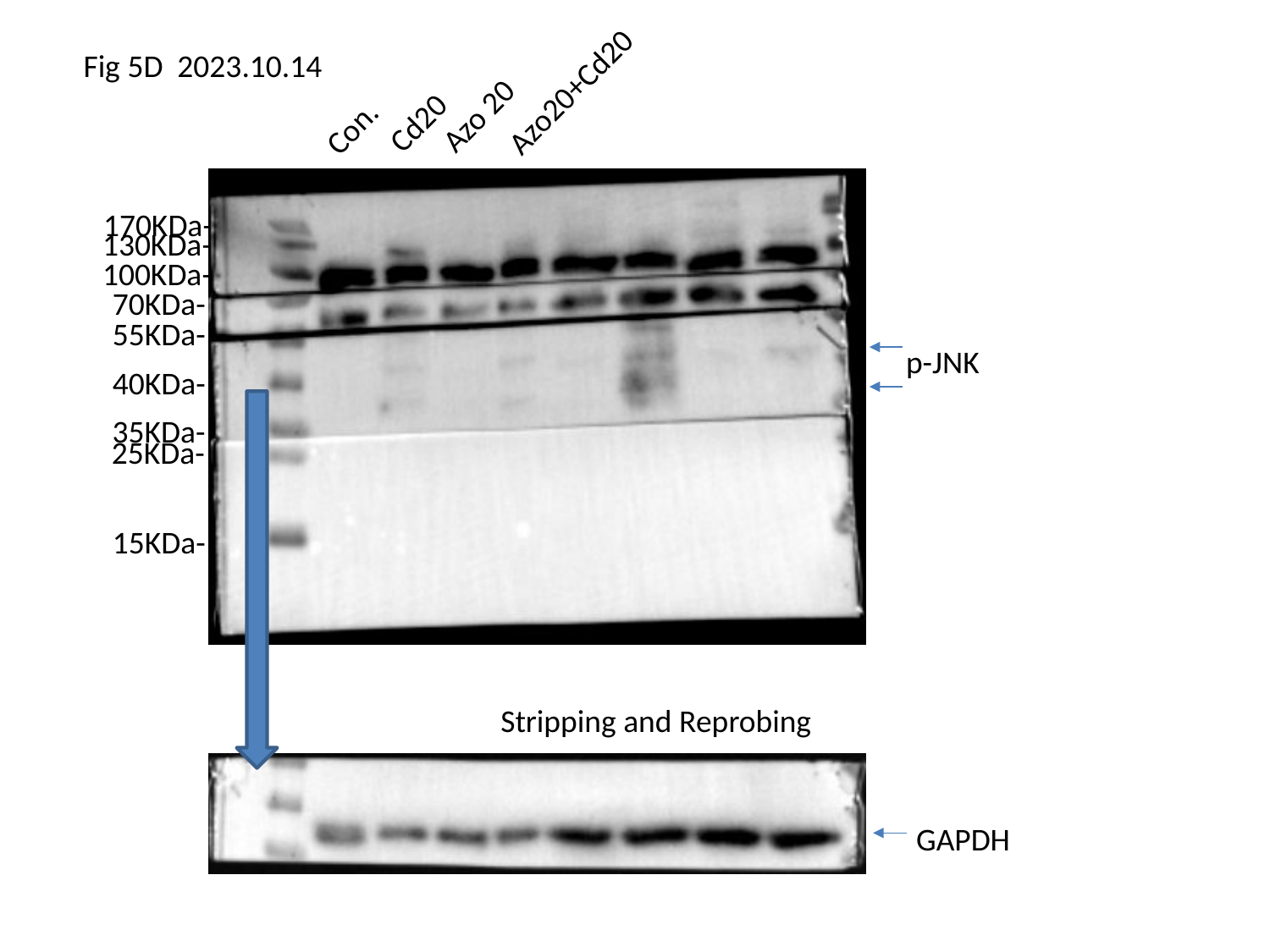

Fig 5D 2023.10.14
Azo20+Cd20
Cd20
Azo 20
Con.
170KDa-
130KDa-
100KDa-
70KDa-
55KDa-
p-JNK
40KDa-
35KDa-
25KDa-
15KDa-
Stripping and Reprobing
GAPDH

## Slide 38
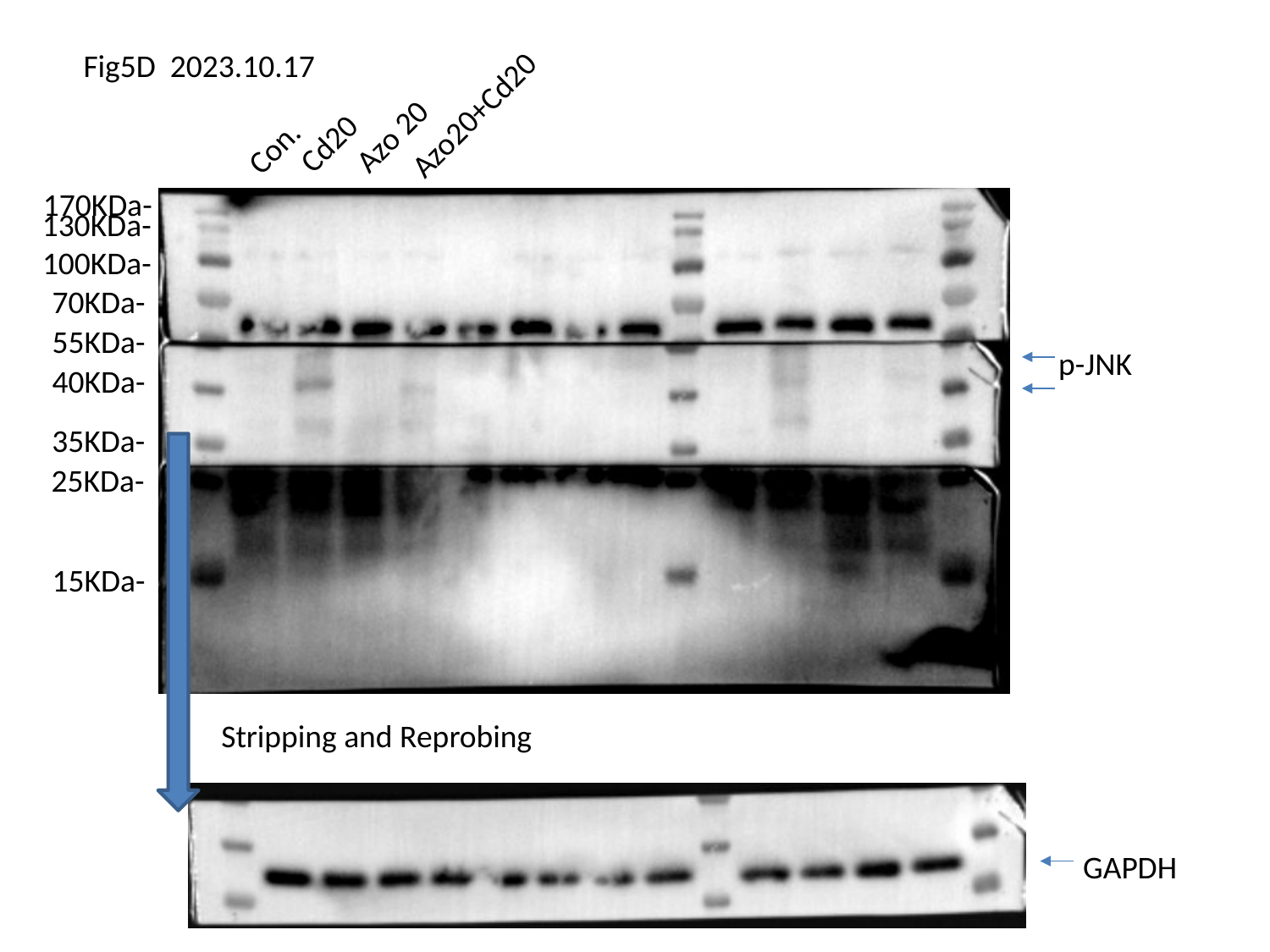

Fig5D 2023.10.17
Azo20+Cd20
Cd20
Azo 20
Con.
170KDa-
130KDa-
100KDa-
70KDa-
55KDa-
p-JNK
40KDa-
35KDa-
25KDa-
15KDa-
Stripping and Reprobing
GAPDH

## Slide 39
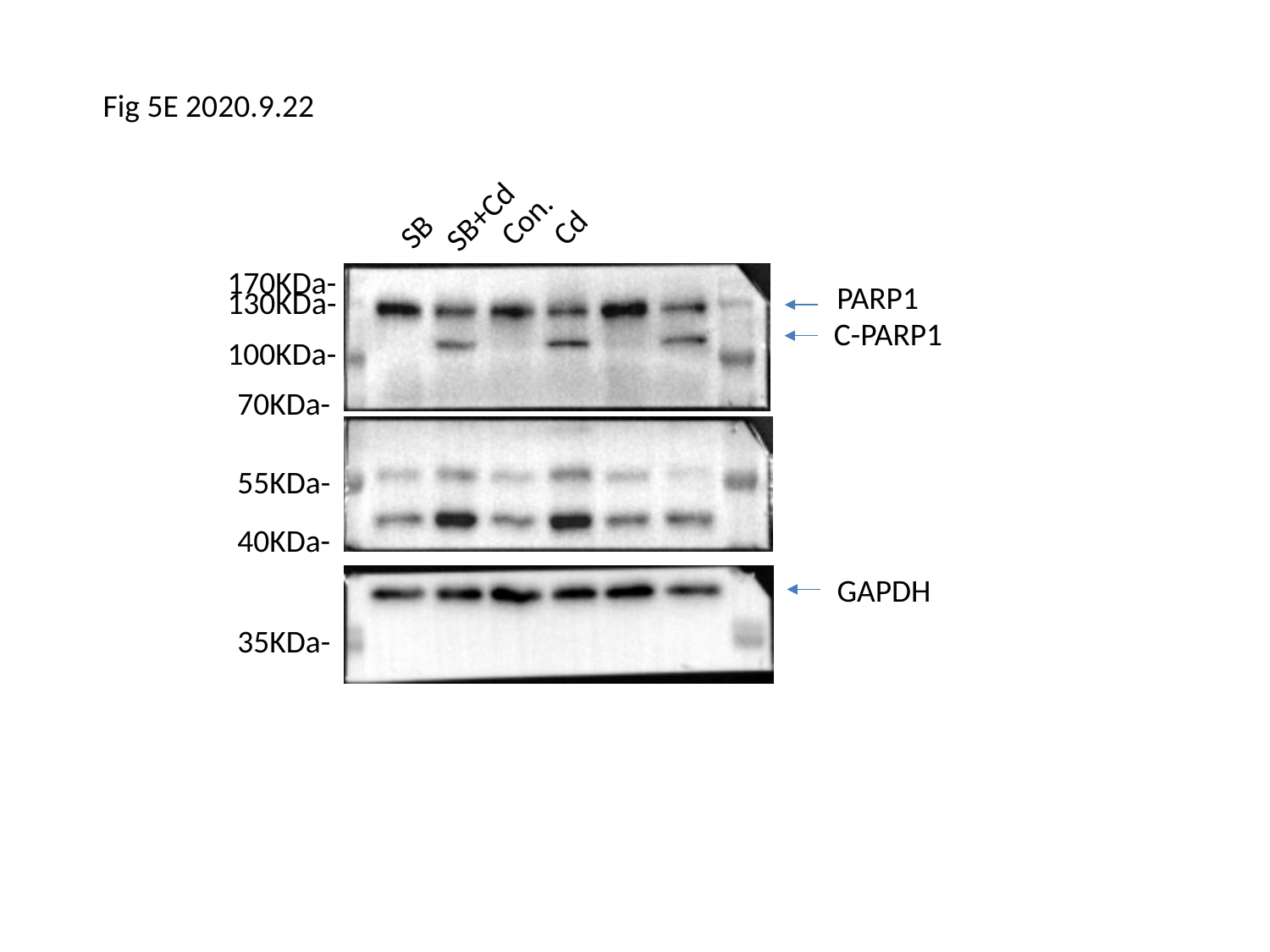

Fig 5E 2020.9.22
SB+Cd
Con.
Cd
SB
170KDa-
130KDa-
100KDa-
70KDa-
55KDa-
40KDa-
35KDa-
PARP1
C-PARP1
GAPDH

## Slide 40
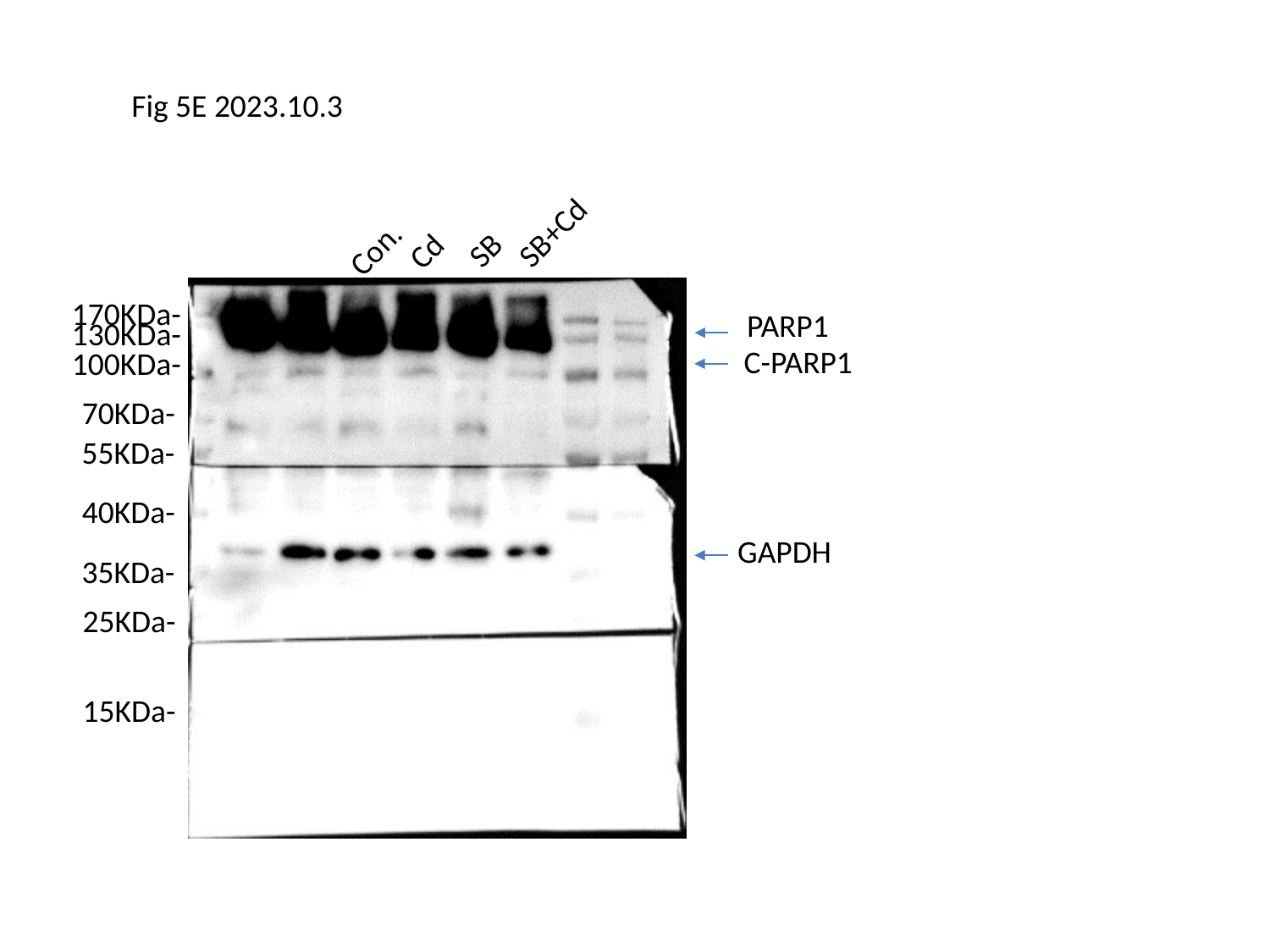

Fig 5E 2023.10.3
SB+Cd
Con.
Cd
SB
170KDa-
130KDa-
100KDa-
70KDa-
55KDa-
40KDa-
35KDa-
PARP1
C-PARP1
GAPDH
25KDa-
15KDa-

## Slide 41
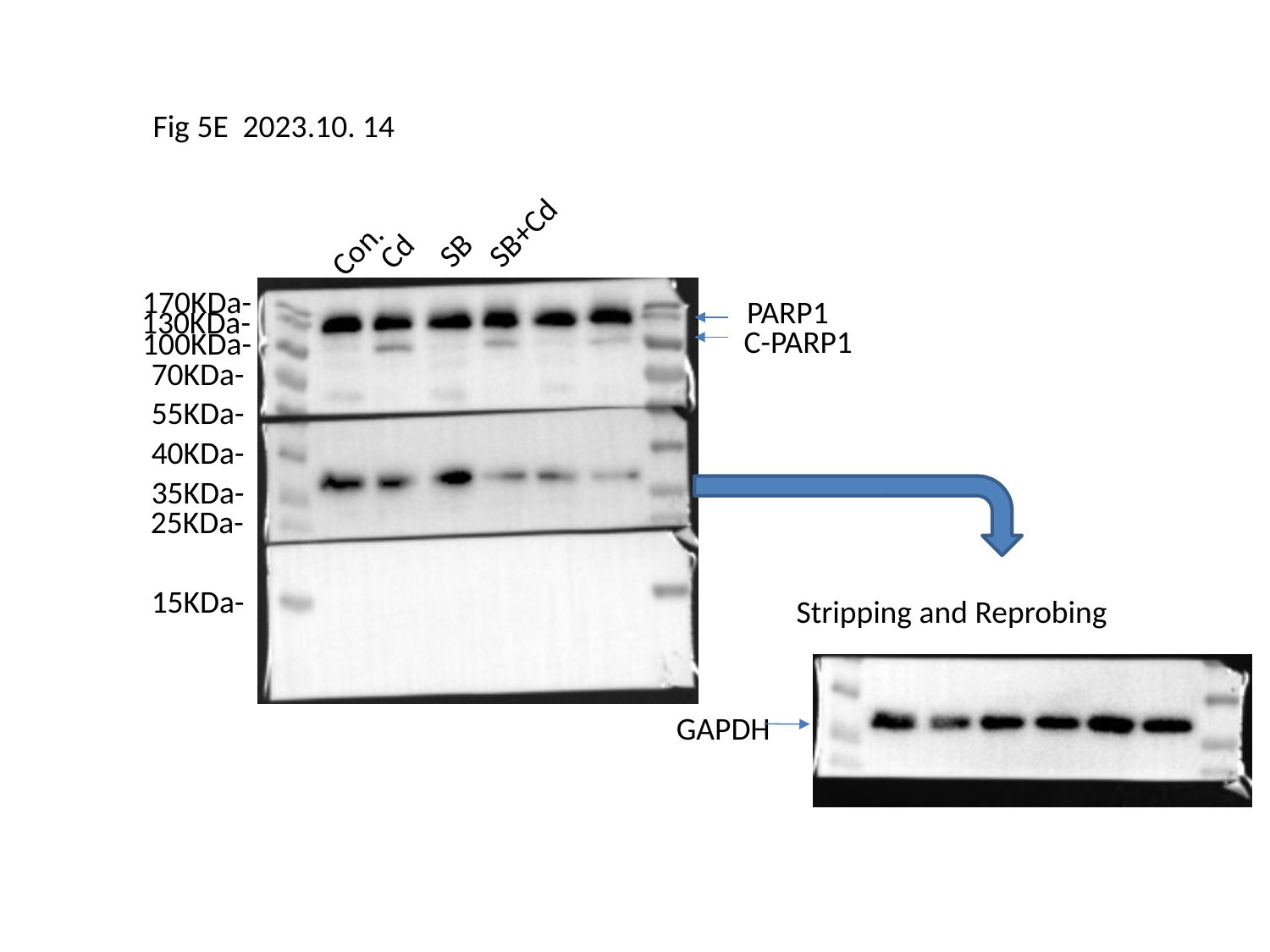

Fig 5E 2023.10. 14
SB+Cd
Con.
Cd
SB
170KDa-
PARP1
130KDa-
C-PARP1
100KDa-
70KDa-
55KDa-
40KDa-
35KDa-
25KDa-
15KDa-
Stripping and Reprobing
GAPDH

## Slide 42
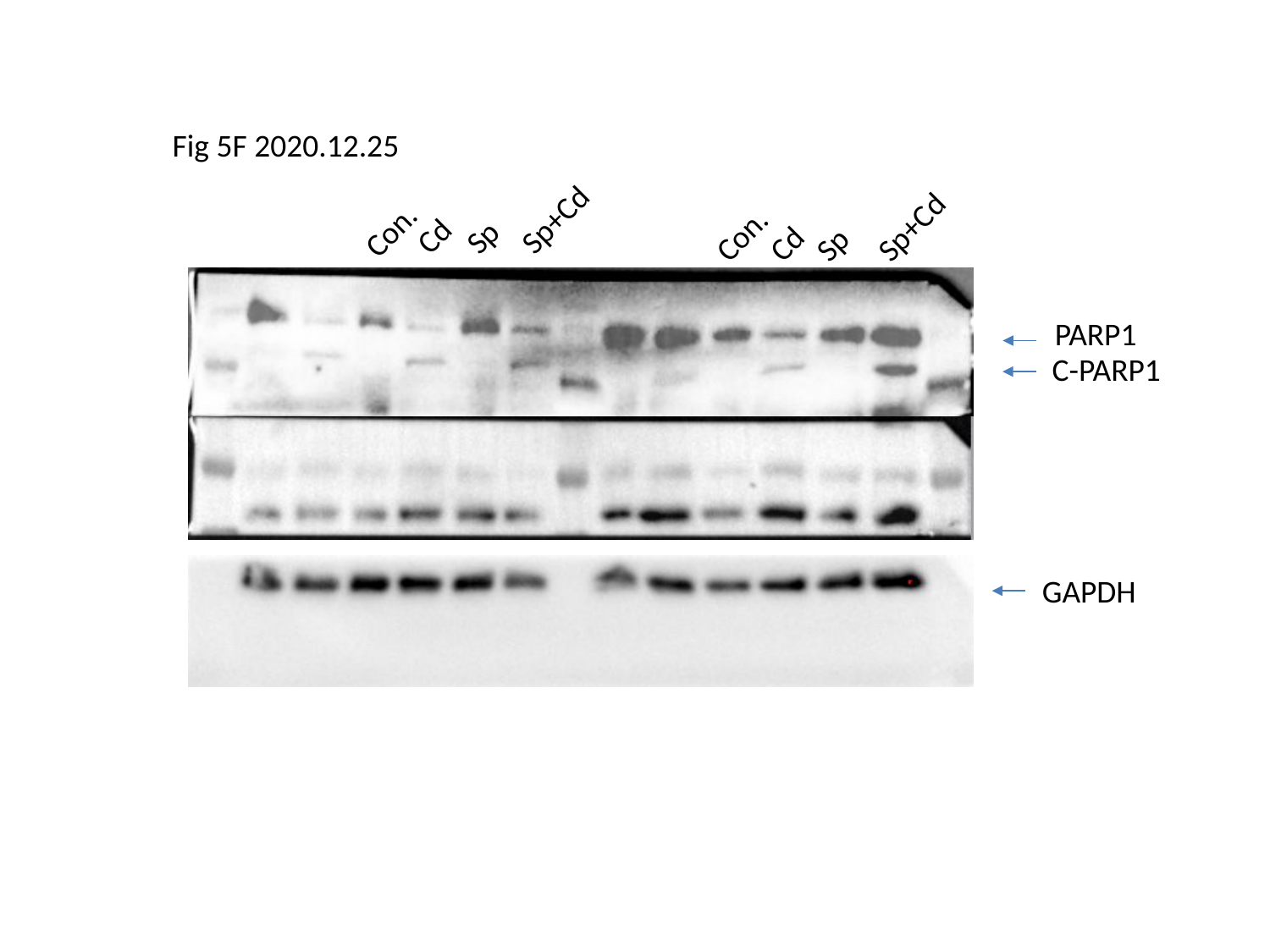

Fig 5F 2020.12.25
Sp+Cd
Sp+Cd
Cd
Cd
Sp
Sp
Con.
Con.
PARP1
C-PARP1
GAPDH

## Slide 43
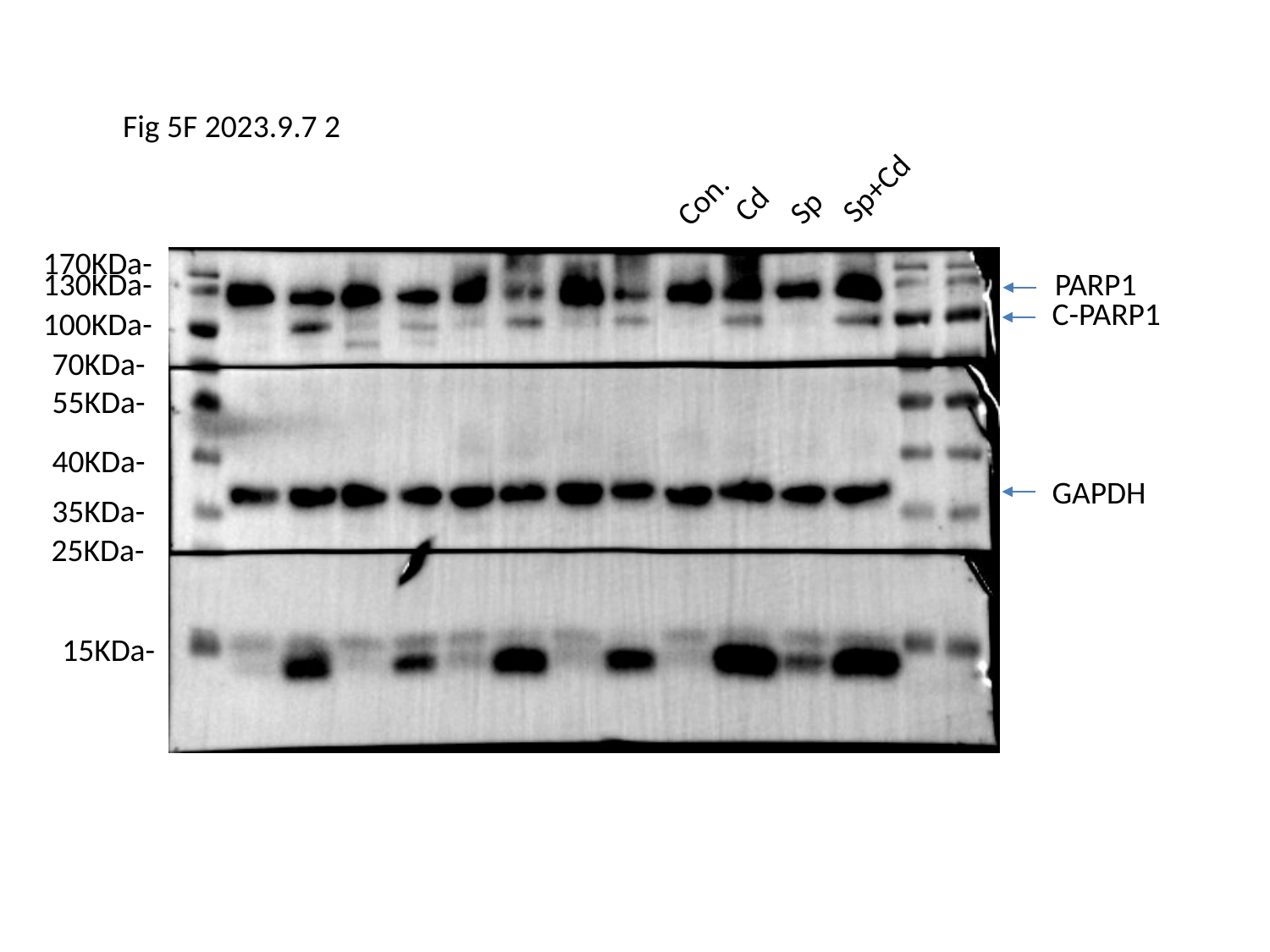

Fig 5F 2023.9.7 2
Sp+Cd
Cd
Sp
Con.
170KDa-
PARP1
130KDa-
C-PARP1
100KDa-
70KDa-
55KDa-
40KDa-
GAPDH
35KDa-
25KDa-
15KDa-

## Slide 44
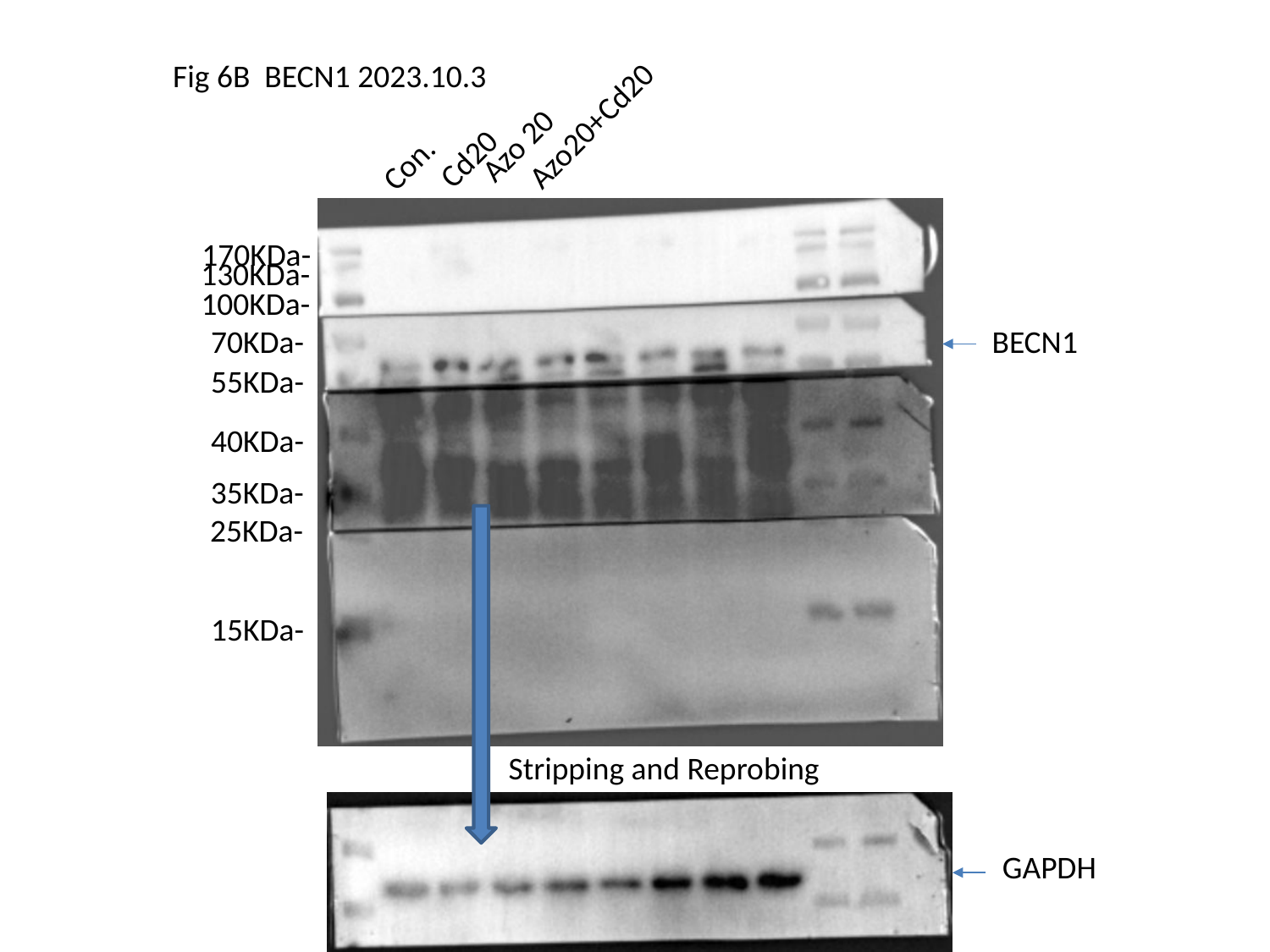

Fig 6B BECN1 2023.10.3
Azo20+Cd20
Cd20
Azo 20
Con.
170KDa-
130KDa-
100KDa-
70KDa-
BECN1
55KDa-
40KDa-
35KDa-
25KDa-
15KDa-
Stripping and Reprobing
GAPDH

## Slide 45
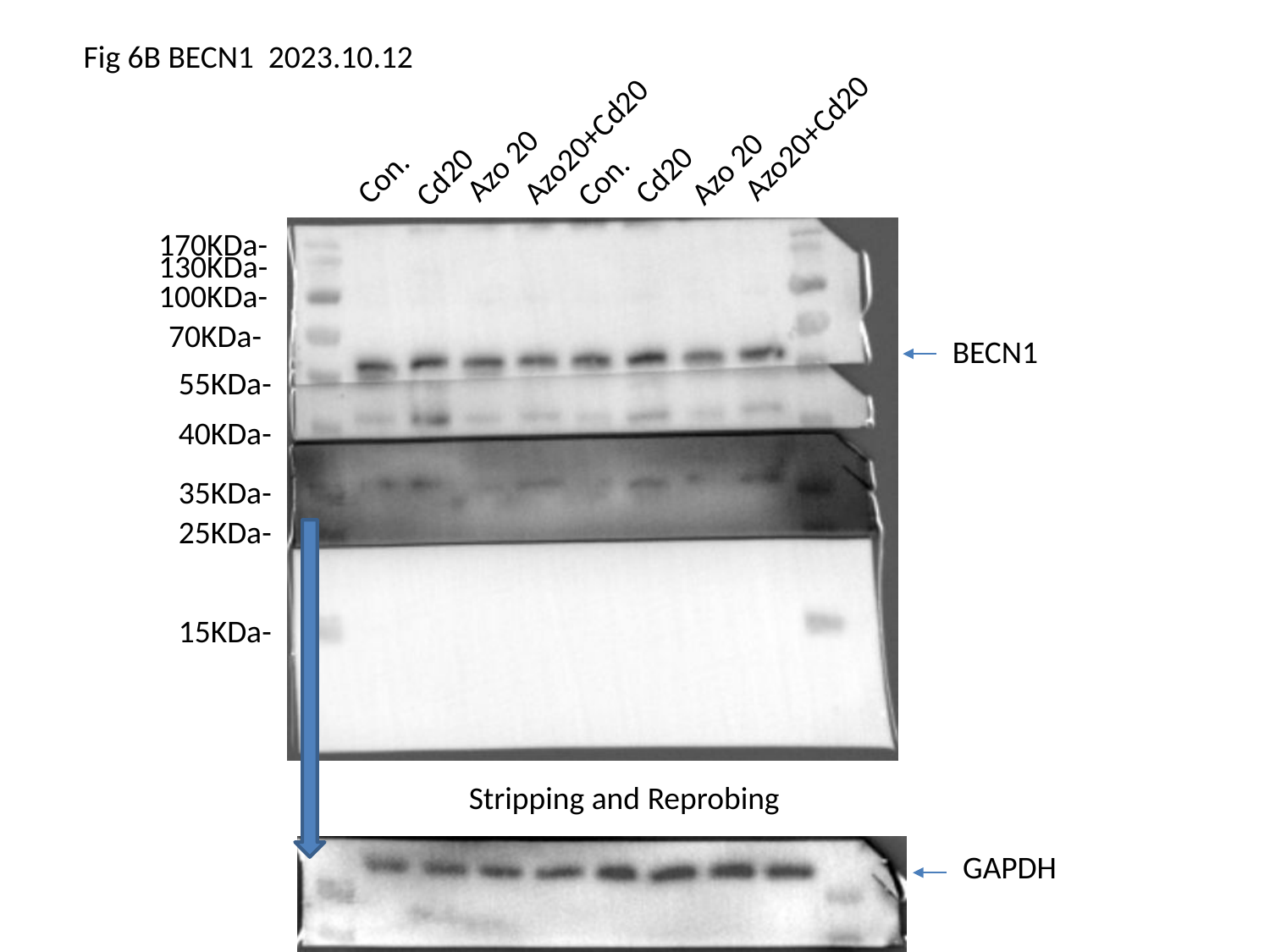

Fig 6B BECN1 2023.10.12
Azo20+Cd20
Azo20+Cd20
Cd20
Cd20
Azo 20
Azo 20
Con.
Con.
170KDa-
130KDa-
100KDa-
70KDa-
BECN1
55KDa-
40KDa-
35KDa-
25KDa-
15KDa-
Stripping and Reprobing
GAPDH

## Slide 46
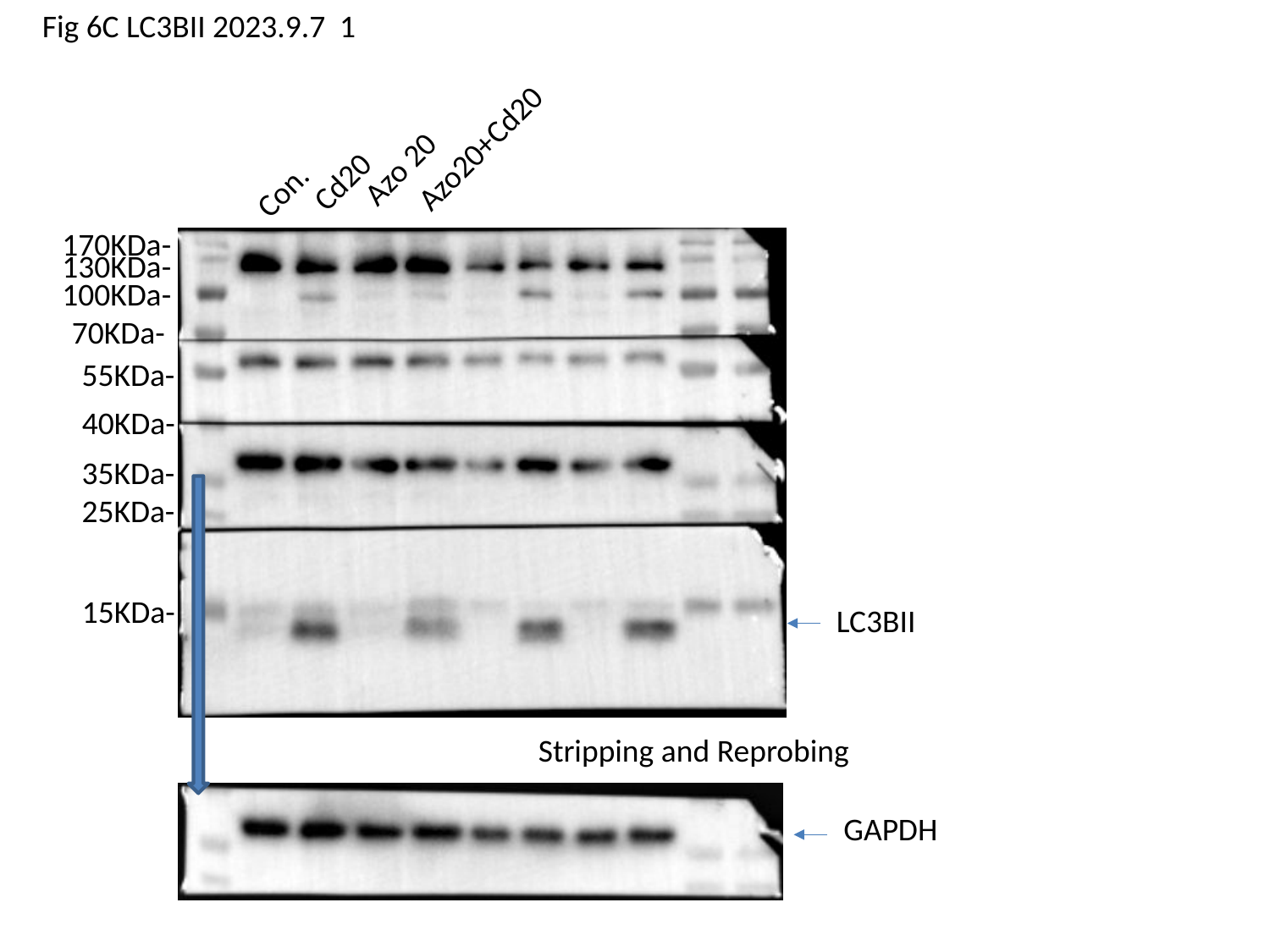

Fig 6C LC3BII 2023.9.7 1
Azo20+Cd20
Cd20
Azo 20
Con.
170KDa-
130KDa-
100KDa-
70KDa-
55KDa-
40KDa-
35KDa-
25KDa-
15KDa-
LC3BII
Stripping and Reprobing
GAPDH

## Slide 47
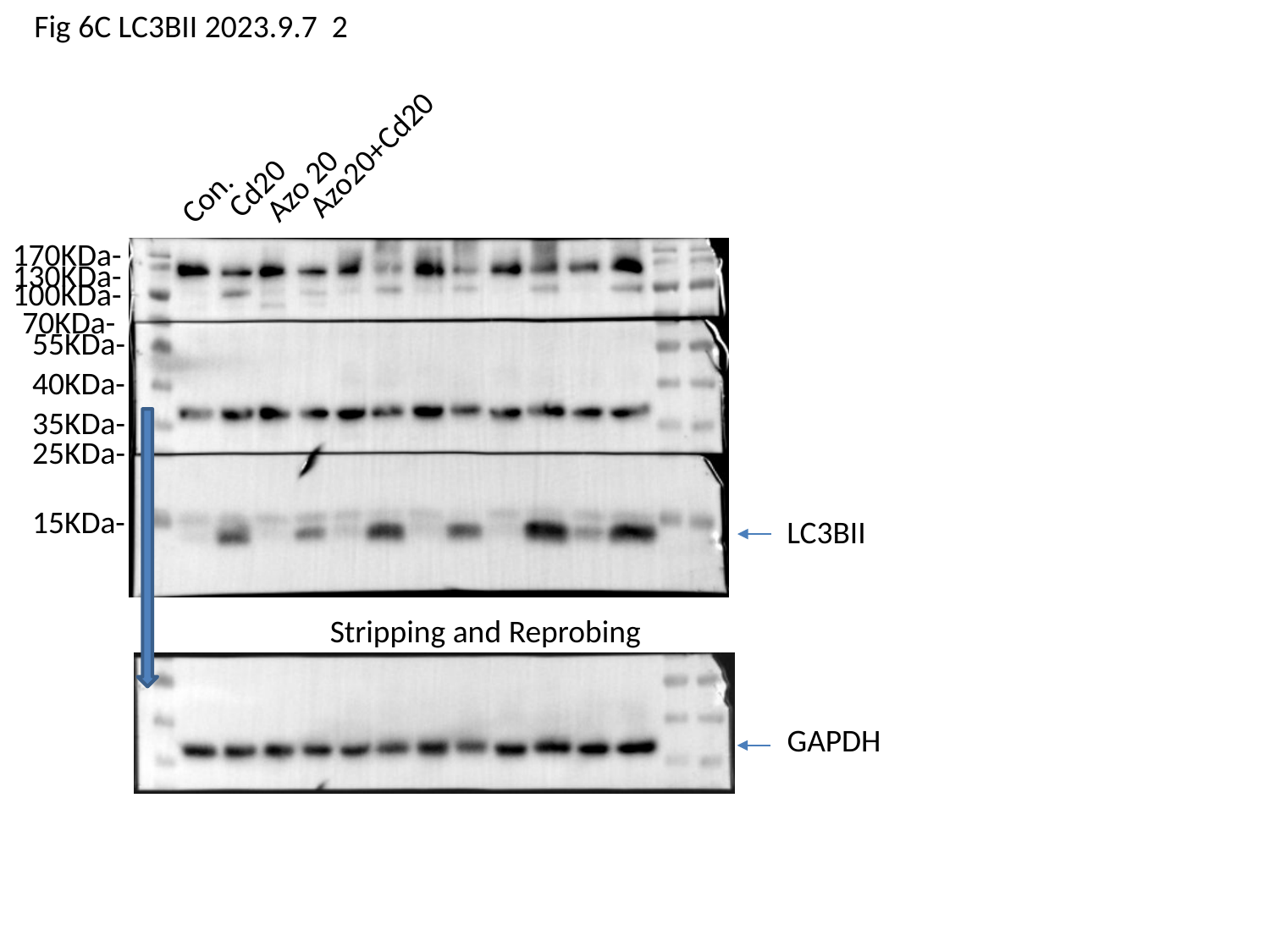

Fig 6C LC3BII 2023.9.7 2
Azo20+Cd20
Cd20
Azo 20
Con.
170KDa-
130KDa-
100KDa-
70KDa-
55KDa-
40KDa-
35KDa-
25KDa-
15KDa-
LC3BII
Stripping and Reprobing
GAPDH

## Slide 48
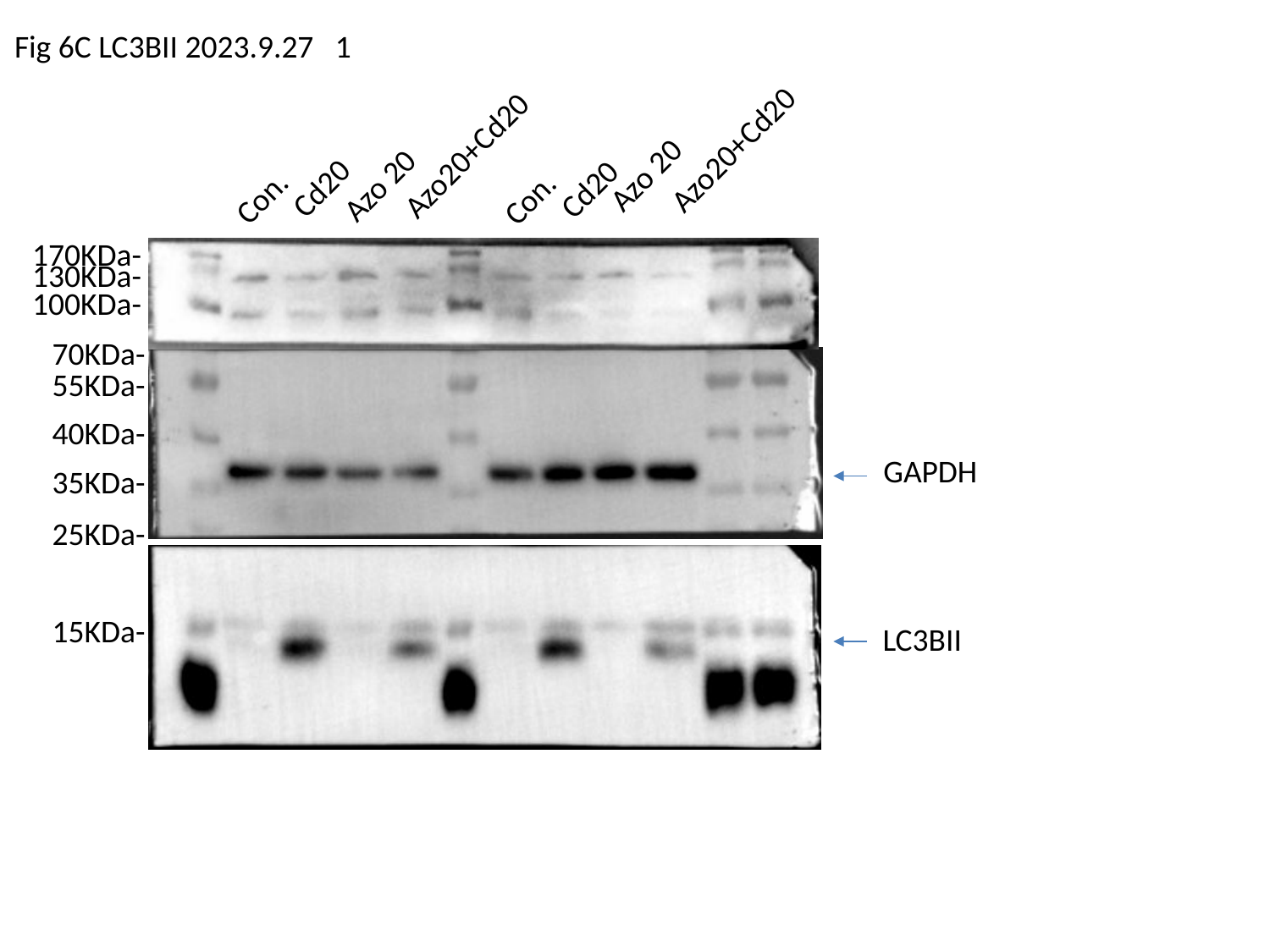

Fig 6C LC3BII 2023.9.27 1
Azo20+Cd20
Azo20+Cd20
Cd20
Cd20
Azo 20
Azo 20
Con.
Con.
170KDa-
130KDa-
100KDa-
70KDa-
55KDa-
40KDa-
GAPDH
35KDa-
25KDa-
15KDa-
LC3BII

## Slide 49
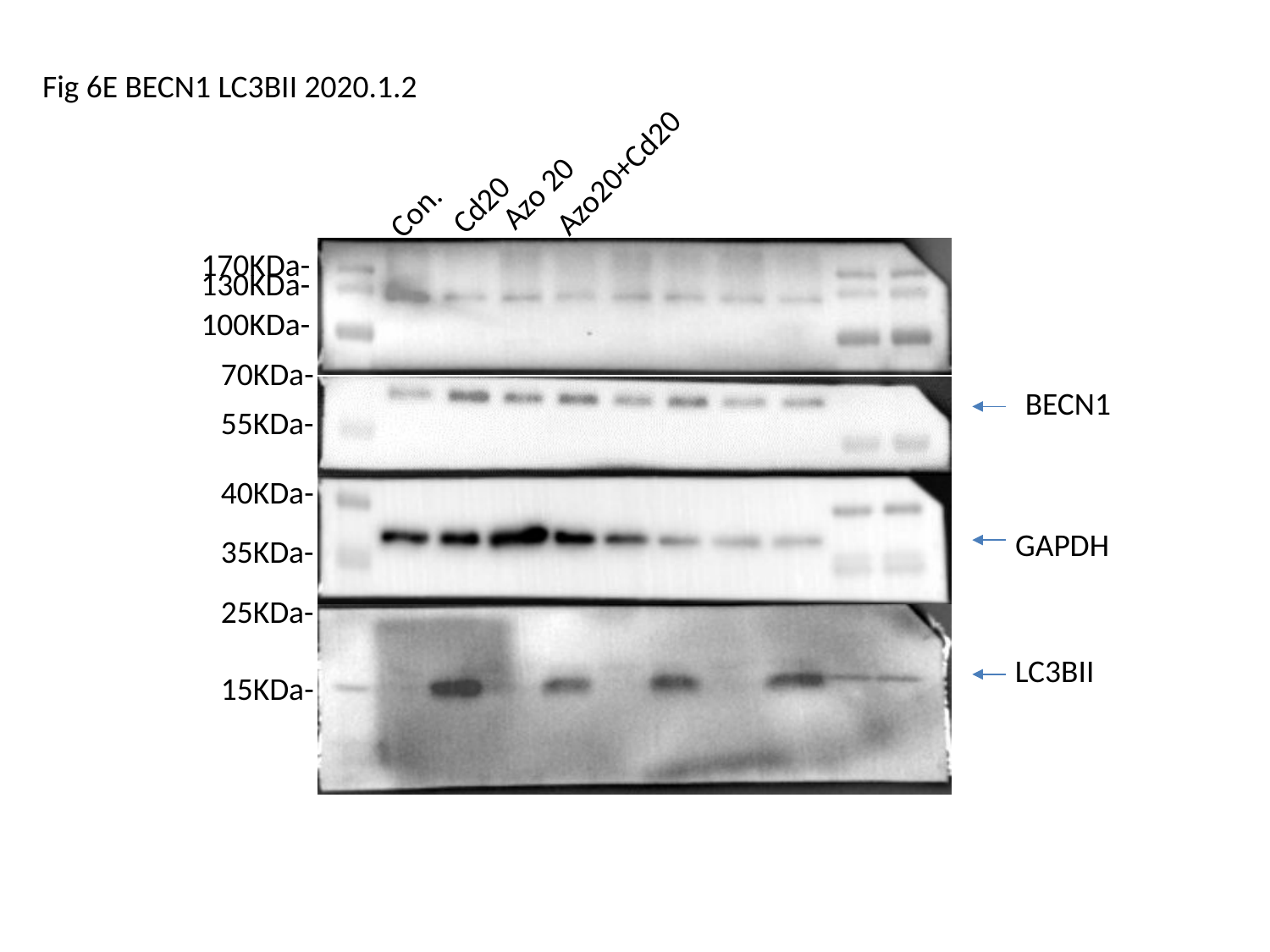

Fig 6E BECN1 LC3BII 2020.1.2
170KDa-
130KDa-
100KDa-
70KDa-
55KDa-
40KDa-
35KDa-
25KDa-
15KDa-
Azo20+Cd20
Azo 20
Con.
Cd20
BECN1
GAPDH
LC3BII

## Slide 50
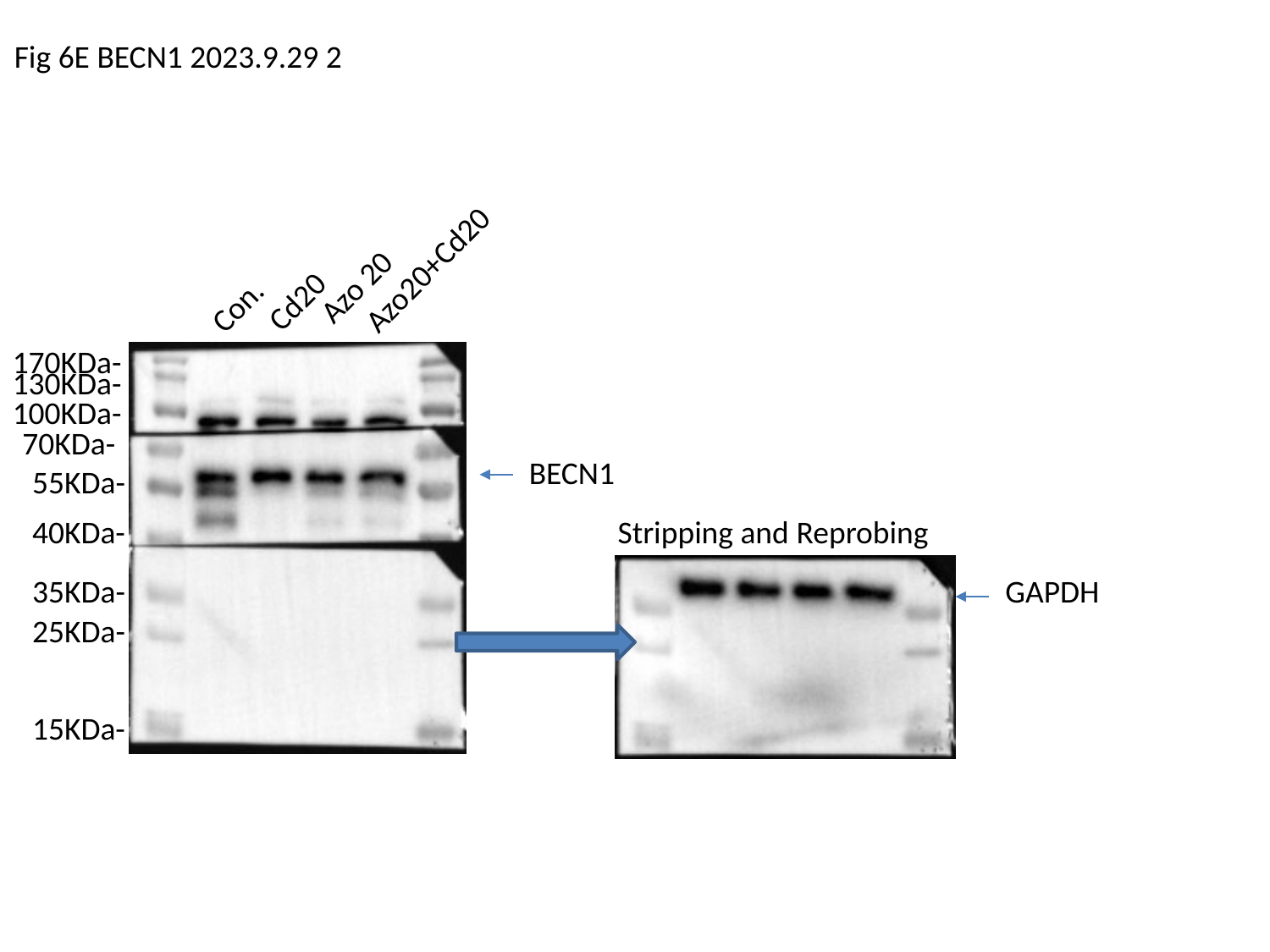

Fig 6E BECN1 2023.9.29 2
Azo20+Cd20
Cd20
Azo 20
Con.
170KDa-
130KDa-
100KDa-
70KDa-
BECN1
55KDa-
40KDa-
Stripping and Reprobing
35KDa-
GAPDH
25KDa-
15KDa-

## Slide 51
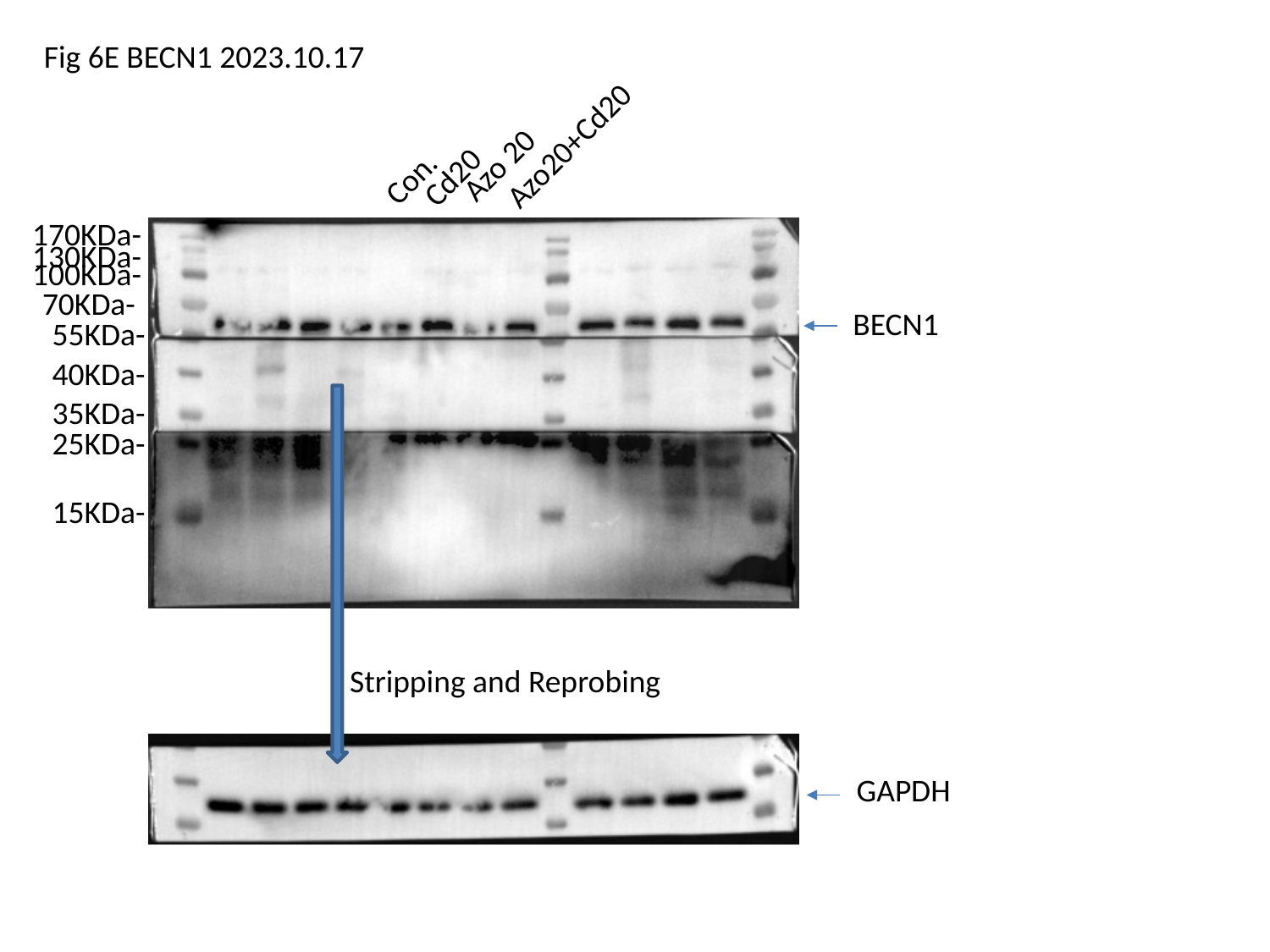

Fig 6E BECN1 2023.10.17
Azo20+Cd20
Cd20
Azo 20
Con.
170KDa-
130KDa-
100KDa-
70KDa-
BECN1
55KDa-
40KDa-
35KDa-
25KDa-
15KDa-
Stripping and Reprobing
GAPDH

## Slide 52
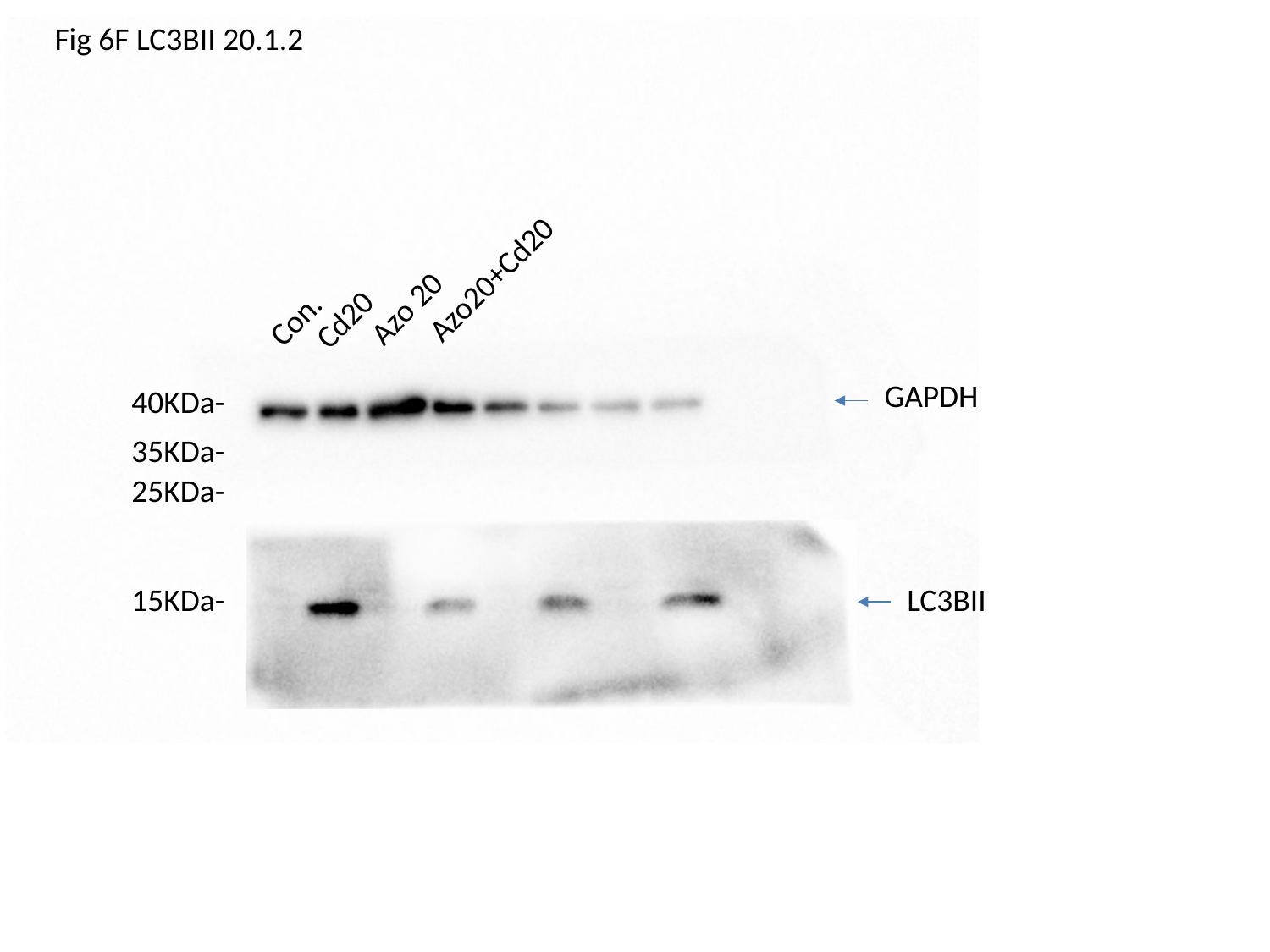

Fig 6F LC3BII 20.1.2
Azo20+Cd20
Cd20
Azo 20
Con.
GAPDH
40KDa-
35KDa-
25KDa-
15KDa-
LC3BII

## Slide 53
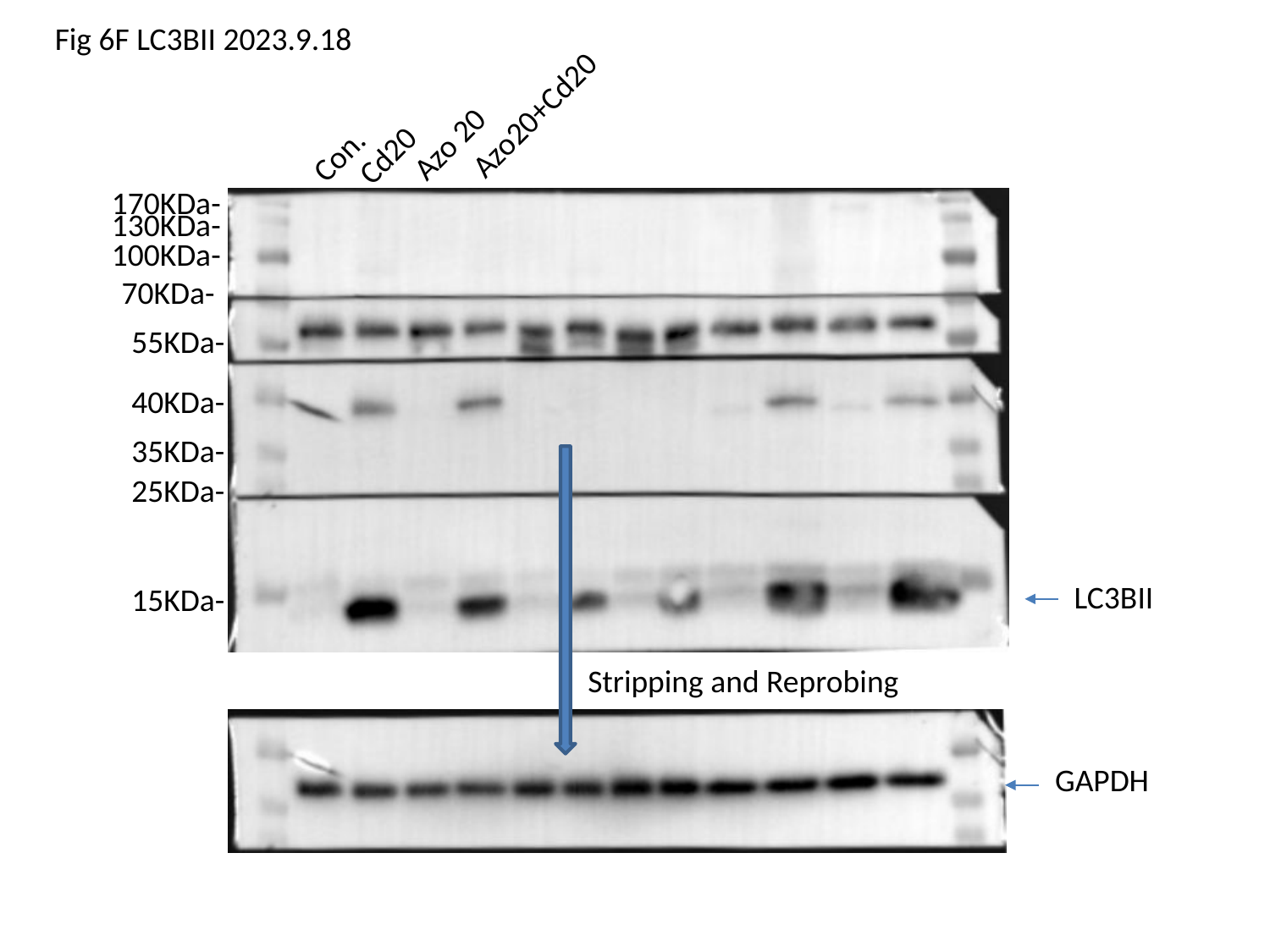

Fig 6F LC3BII 2023.9.18
Azo20+Cd20
Cd20
Azo 20
Con.
170KDa-
130KDa-
100KDa-
70KDa-
55KDa-
40KDa-
35KDa-
25KDa-
LC3BII
15KDa-
Stripping and Reprobing
GAPDH

## Slide 54
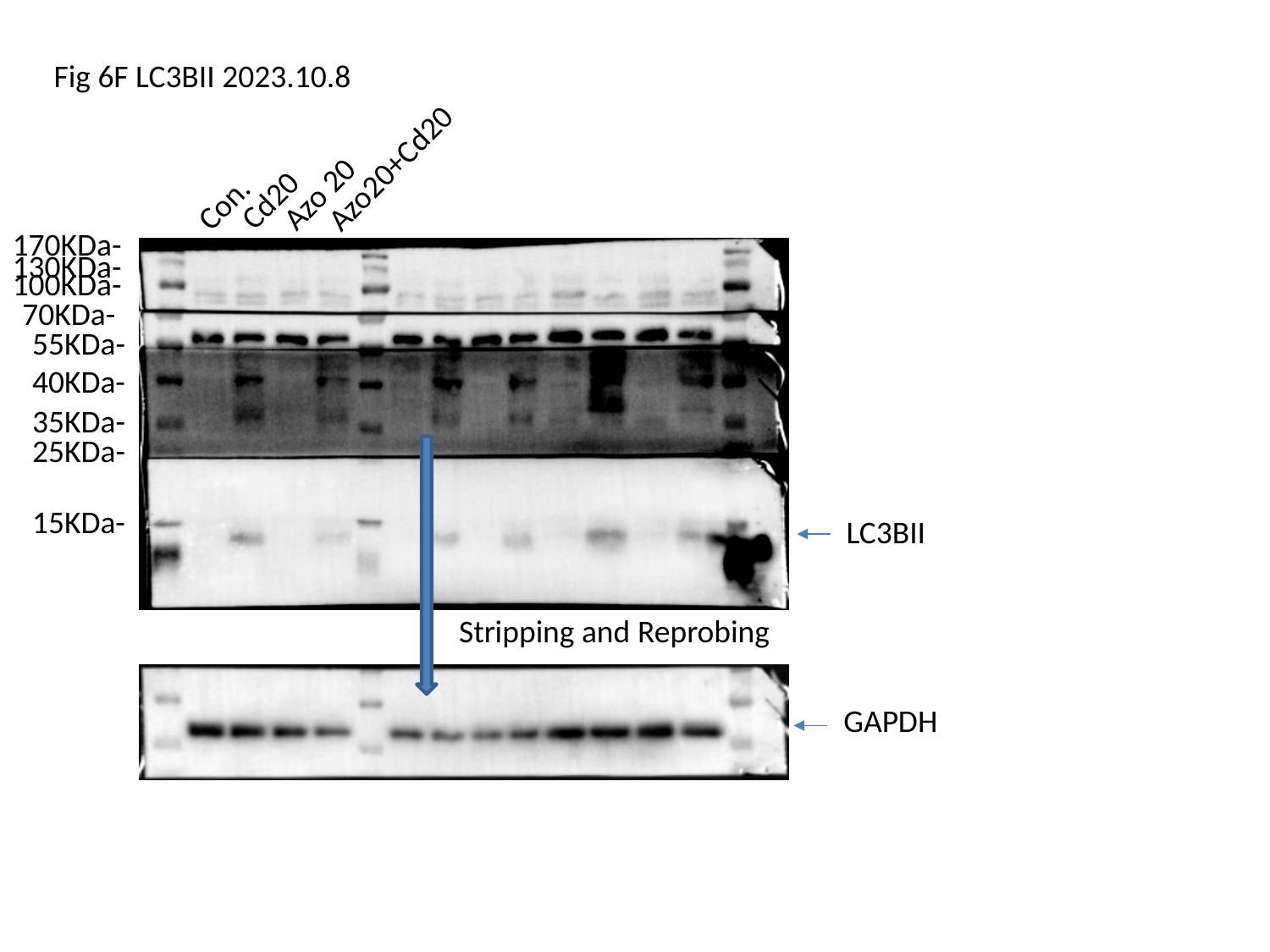

Fig 6F LC3BII 2023.10.8
Azo20+Cd20
Cd20
Azo 20
Con.
170KDa-
130KDa-
100KDa-
70KDa-
55KDa-
40KDa-
35KDa-
25KDa-
15KDa-
LC3BII
Stripping and Reprobing
GAPDH
