# Supplementary figures and images for: Azoramide ameliorates cadmium-induced cytotoxicity by inhibiting endoplasmic reticulum stress and suppressing oxidative stress
Source: PeerJ. 2024 Jan 31;12:e16844. doi: 10.7717/peerj.16844 (PMC10838077; doi:10.7717/peerj.16844)

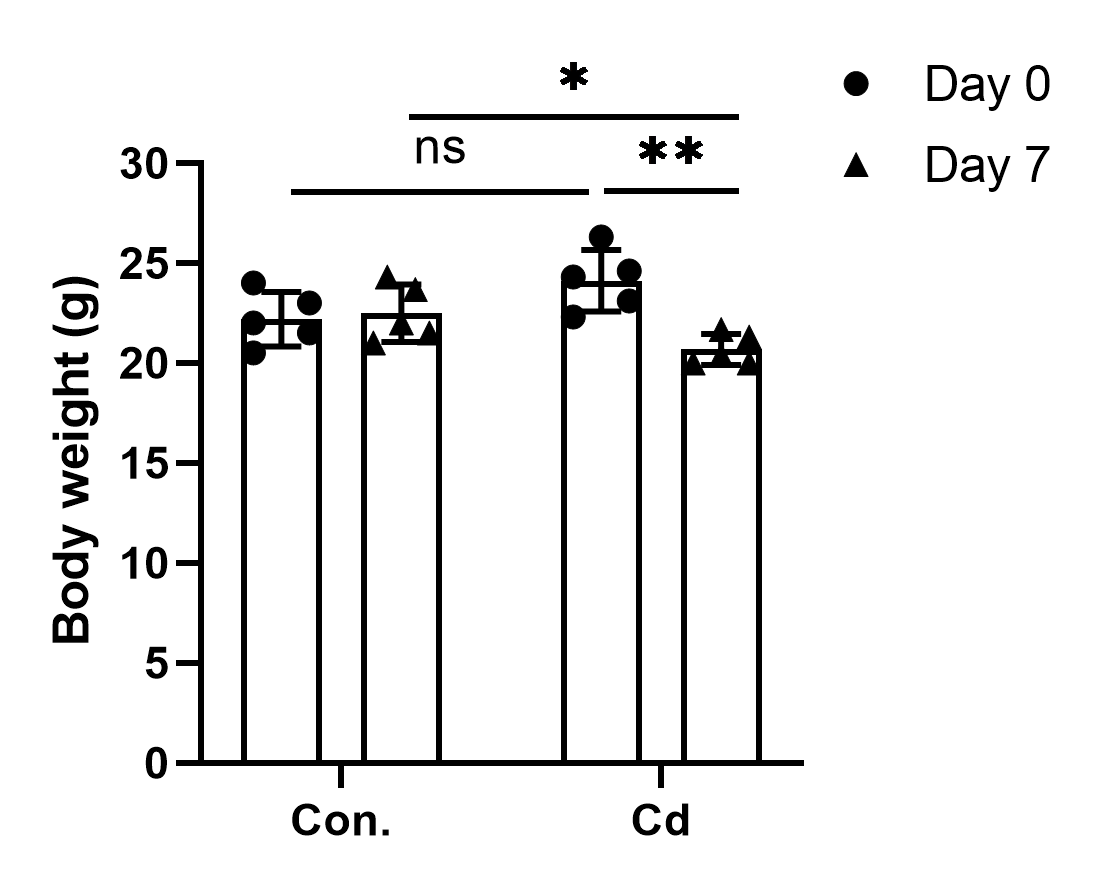

Supplement: Supplemental Information 1 — Files in .PZFX format can be accessed using GraphPad Prism software (version 9.0; San Diego, CA, USA) which can be downloaded from https://www.graphpad.com/. [file peerj-12-16844-s001.zip › Supplemental Files/Fig 1/Fig 1A.tif]

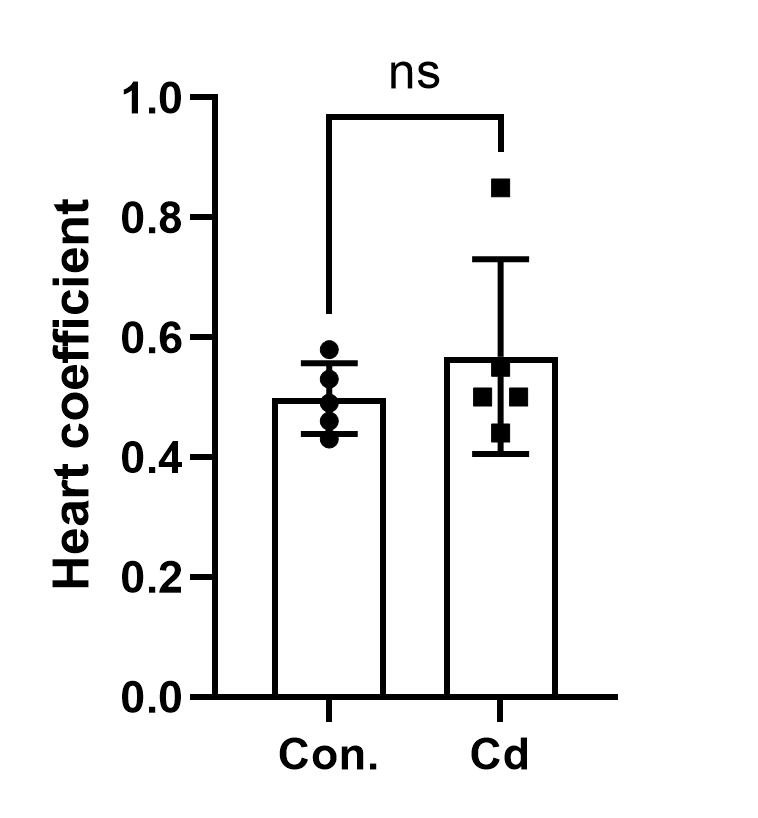

Supplement: Supplemental Information 1 — Files in .PZFX format can be accessed using GraphPad Prism software (version 9.0; San Diego, CA, USA) which can be downloaded from https://www.graphpad.com/. [file peerj-12-16844-s001.zip › Supplemental Files/Fig 1/Fig 1B-new.tif]

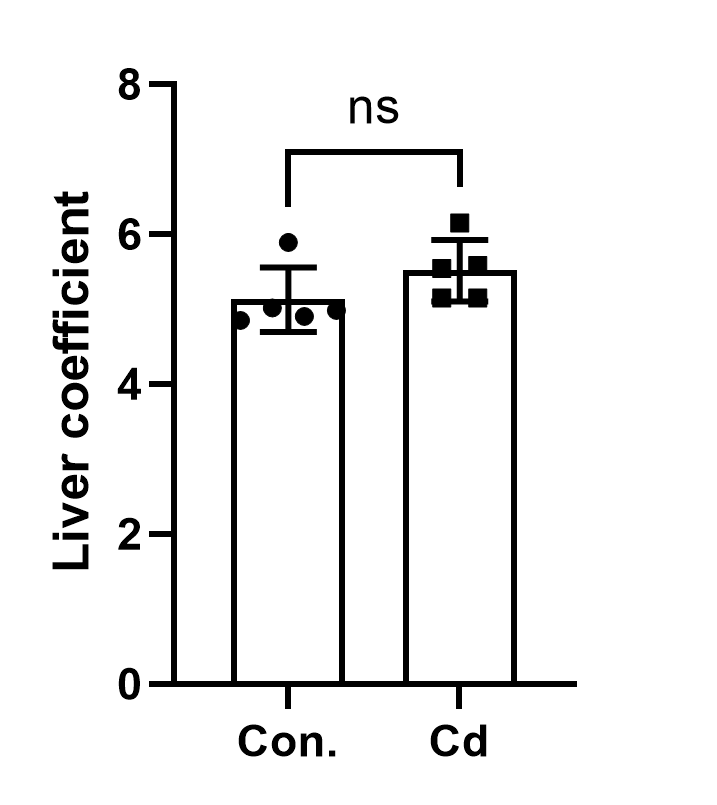

Supplement: Supplemental Information 1 — Files in .PZFX format can be accessed using GraphPad Prism software (version 9.0; San Diego, CA, USA) which can be downloaded from https://www.graphpad.com/. [file peerj-12-16844-s001.zip › Supplemental Files/Fig 1/Fig 1C-new.tif]

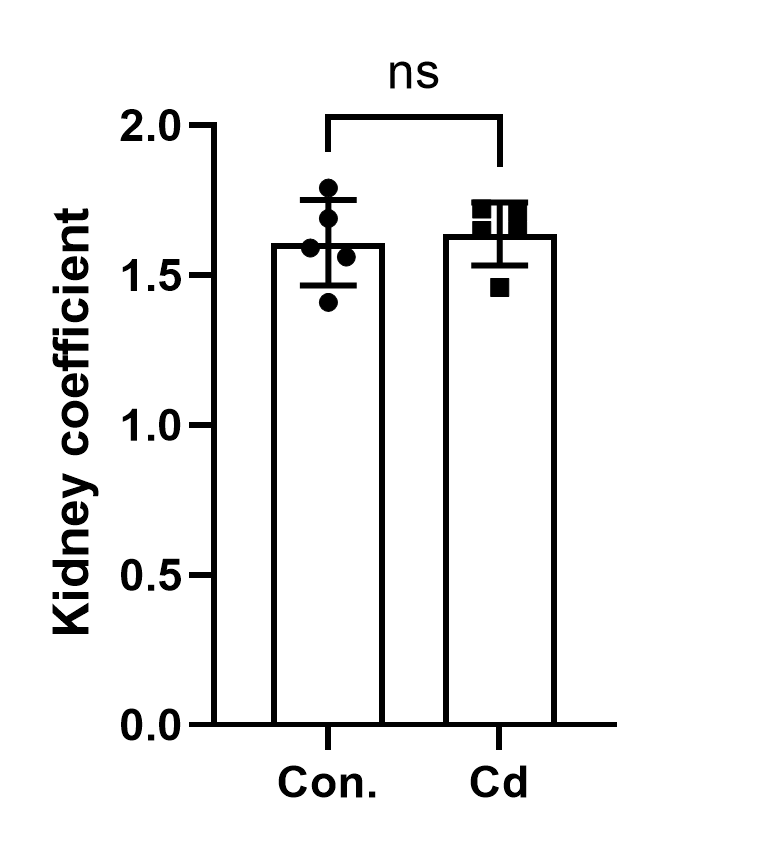

Supplement: Supplemental Information 1 — Files in .PZFX format can be accessed using GraphPad Prism software (version 9.0; San Diego, CA, USA) which can be downloaded from https://www.graphpad.com/. [file peerj-12-16844-s001.zip › Supplemental Files/Fig 1/Fig 1D-new.tif]

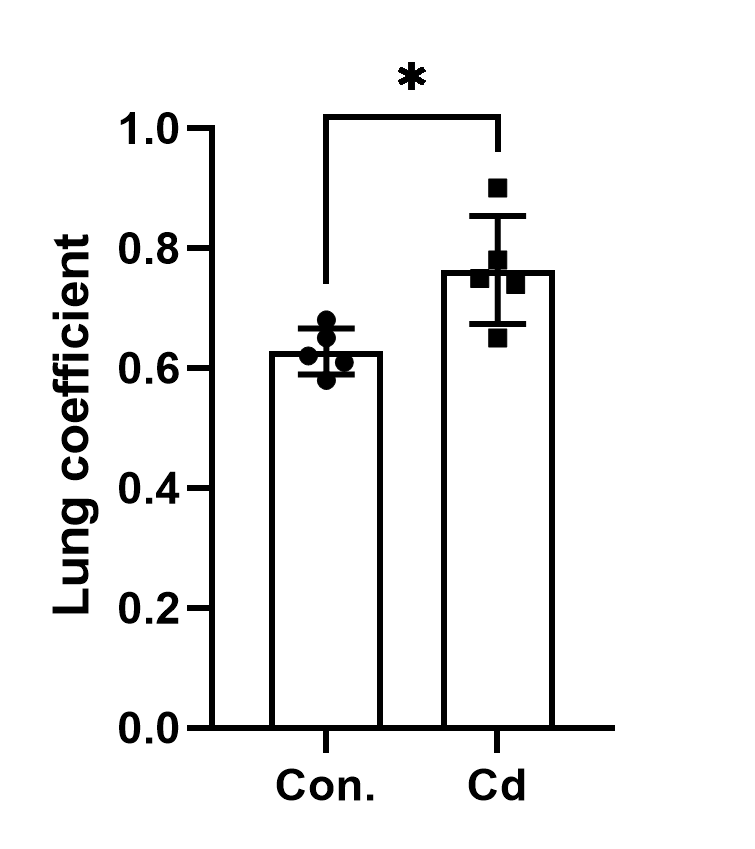

Supplement: Supplemental Information 1 — Files in .PZFX format can be accessed using GraphPad Prism software (version 9.0; San Diego, CA, USA) which can be downloaded from https://www.graphpad.com/. [file peerj-12-16844-s001.zip › Supplemental Files/Fig 1/Fig 1E-new.tif]

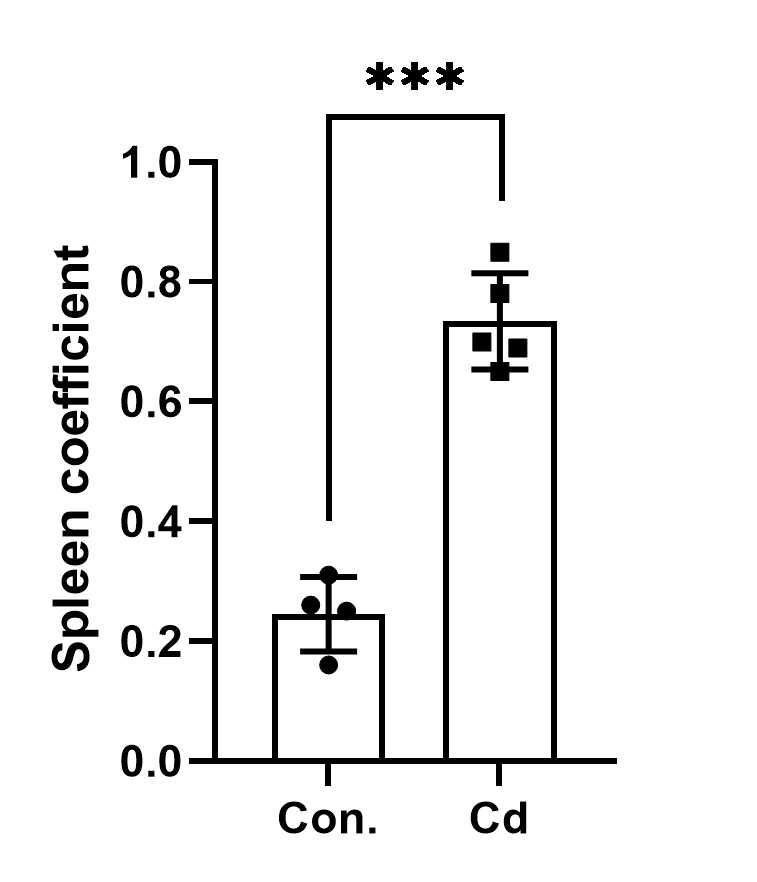

Supplement: Supplemental Information 1 — Files in .PZFX format can be accessed using GraphPad Prism software (version 9.0; San Diego, CA, USA) which can be downloaded from https://www.graphpad.com/. [file peerj-12-16844-s001.zip › Supplemental Files/Fig 1/Fig 1F-new.tif]

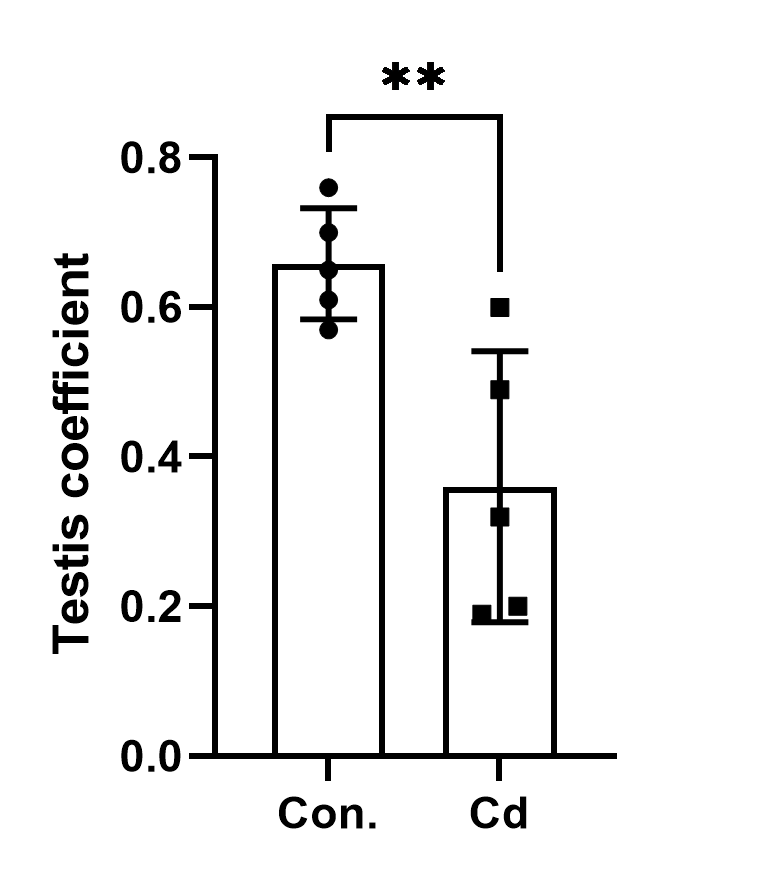

Supplement: Supplemental Information 1 — Files in .PZFX format can be accessed using GraphPad Prism software (version 9.0; San Diego, CA, USA) which can be downloaded from https://www.graphpad.com/. [file peerj-12-16844-s001.zip › Supplemental Files/Fig 1/Fig 1G-new.tif]

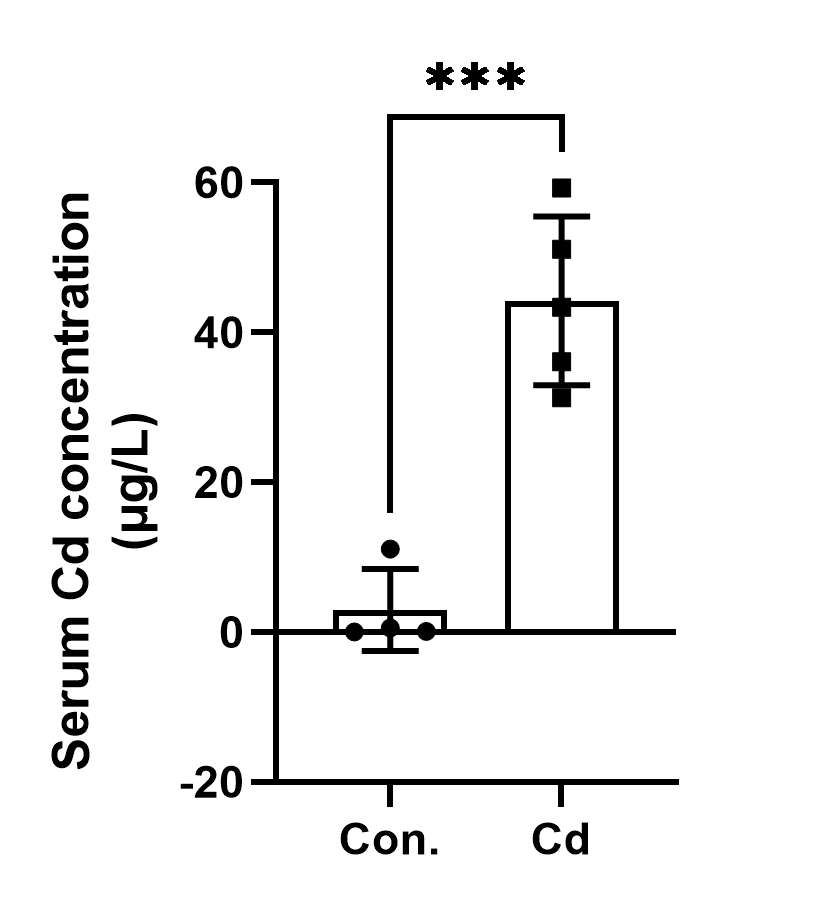

Supplement: Supplemental Information 1 — Files in .PZFX format can be accessed using GraphPad Prism software (version 9.0; San Diego, CA, USA) which can be downloaded from https://www.graphpad.com/. [file peerj-12-16844-s001.zip › Supplemental Files/Fig 1/Fig 1H.tif]

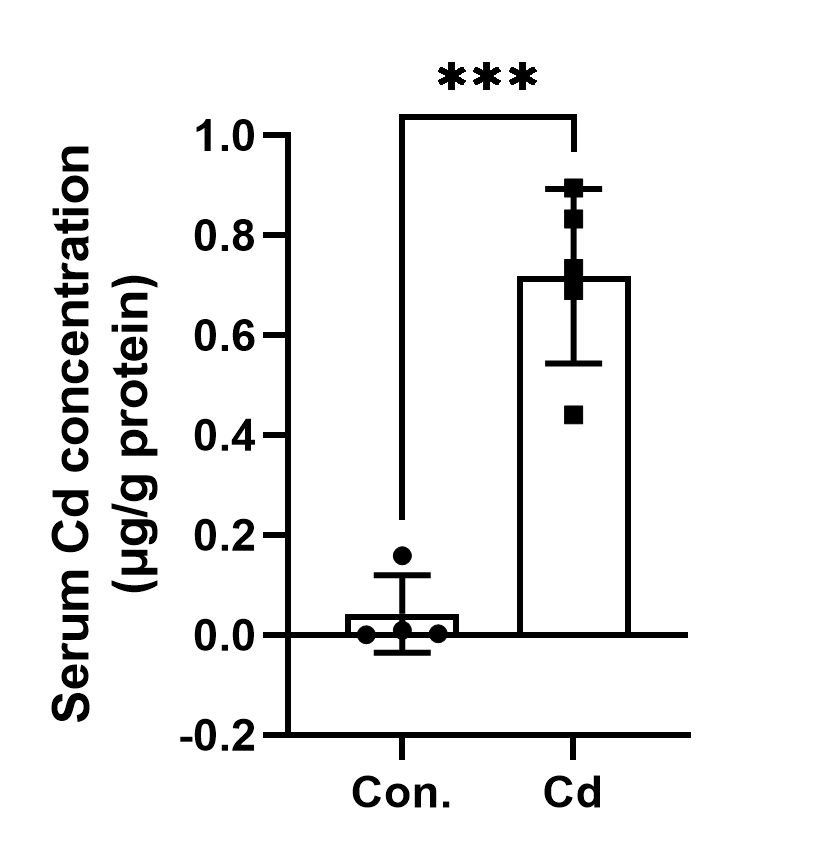

Supplement: Supplemental Information 1 — Files in .PZFX format can be accessed using GraphPad Prism software (version 9.0; San Diego, CA, USA) which can be downloaded from https://www.graphpad.com/. [file peerj-12-16844-s001.zip › Supplemental Files/Fig 1/Fig 1I.tif]

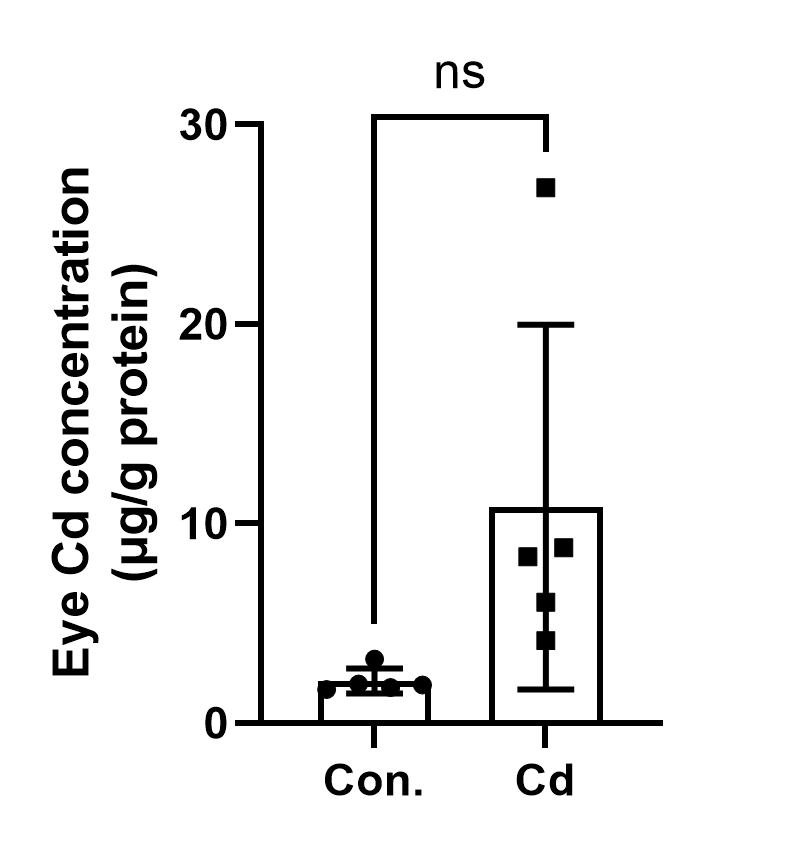

Supplement: Supplemental Information 1 — Files in .PZFX format can be accessed using GraphPad Prism software (version 9.0; San Diego, CA, USA) which can be downloaded from https://www.graphpad.com/. [file peerj-12-16844-s001.zip › Supplemental Files/Fig 1/Fig 1J.tif]

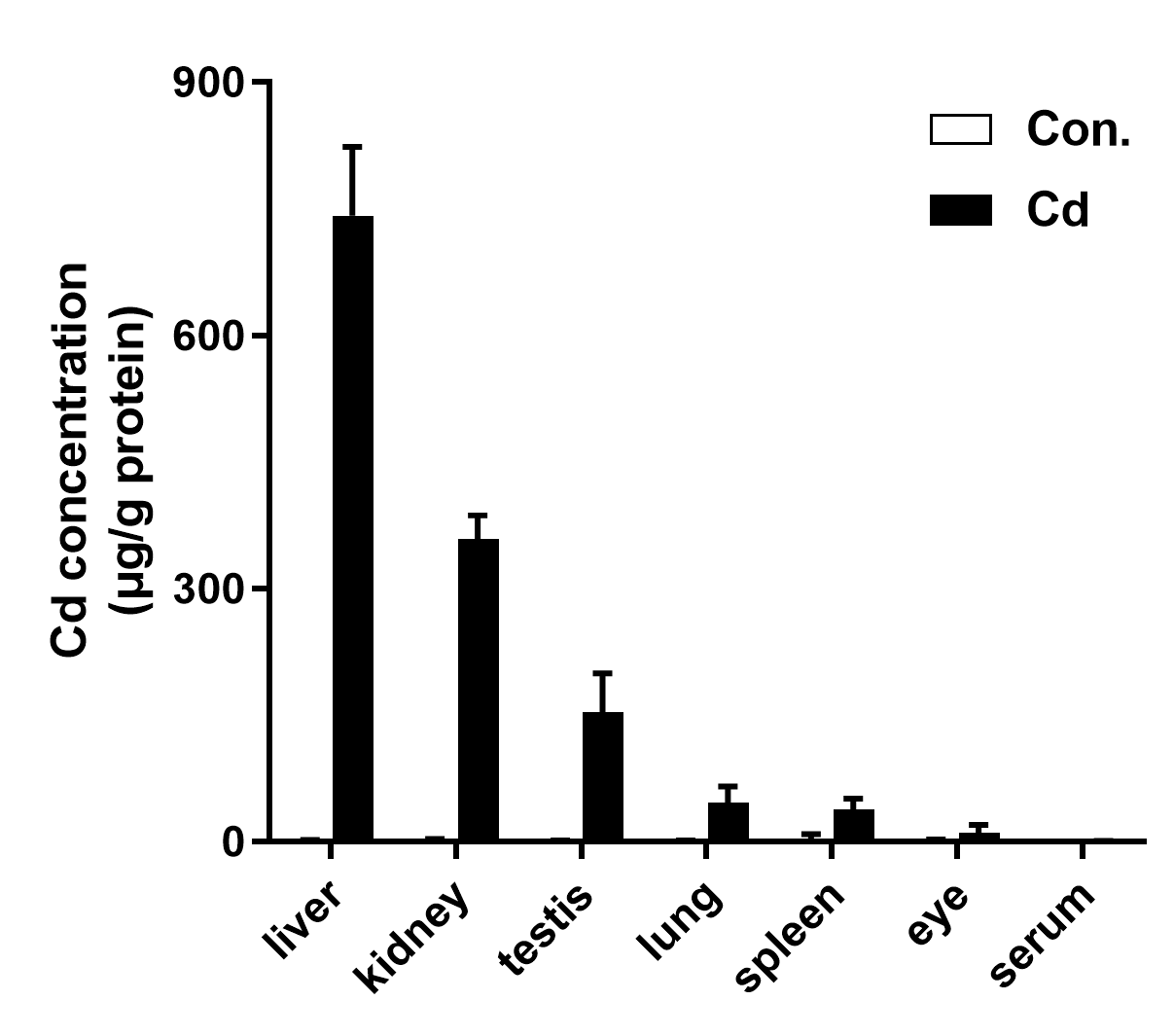

Supplement: Supplemental Information 1 — Files in .PZFX format can be accessed using GraphPad Prism software (version 9.0; San Diego, CA, USA) which can be downloaded from https://www.graphpad.com/. [file peerj-12-16844-s001.zip › Supplemental Files/Fig 1/Fig 1K.tif]

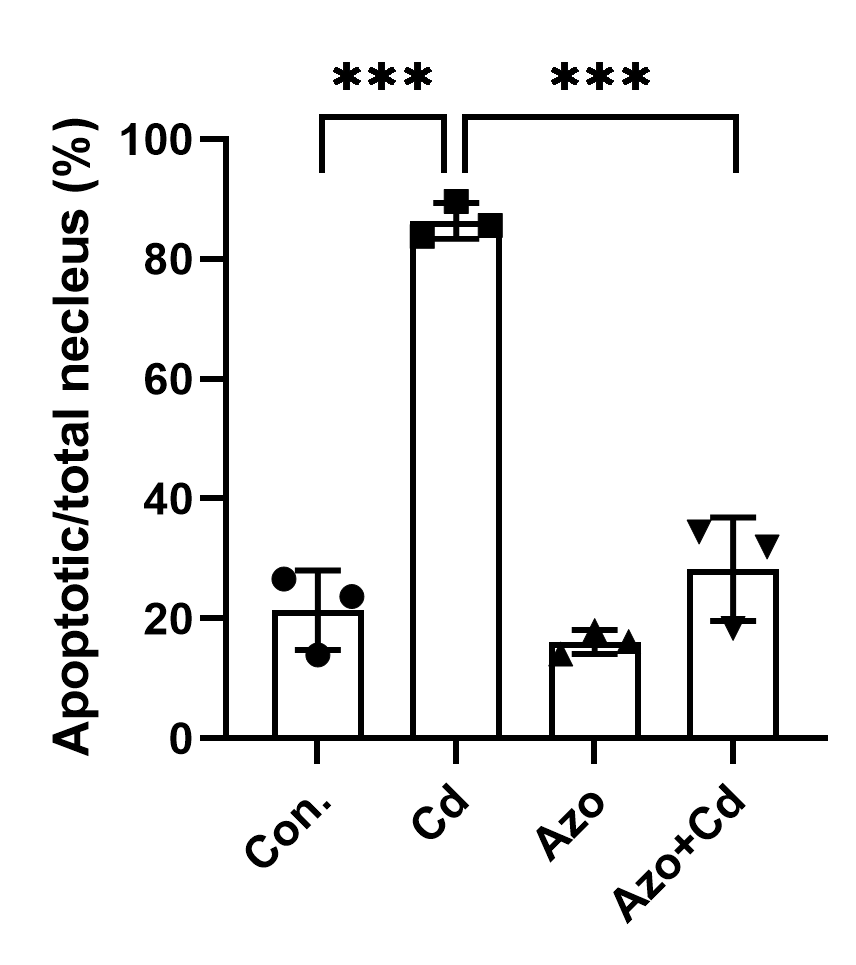

Supplement: Supplemental Information 1 — Files in .PZFX format can be accessed using GraphPad Prism software (version 9.0; San Diego, CA, USA) which can be downloaded from https://www.graphpad.com/. [file peerj-12-16844-s001.zip › Supplemental Files/Fig 2A/Fig 2A HK-2 DAPI staining analysis.tif]

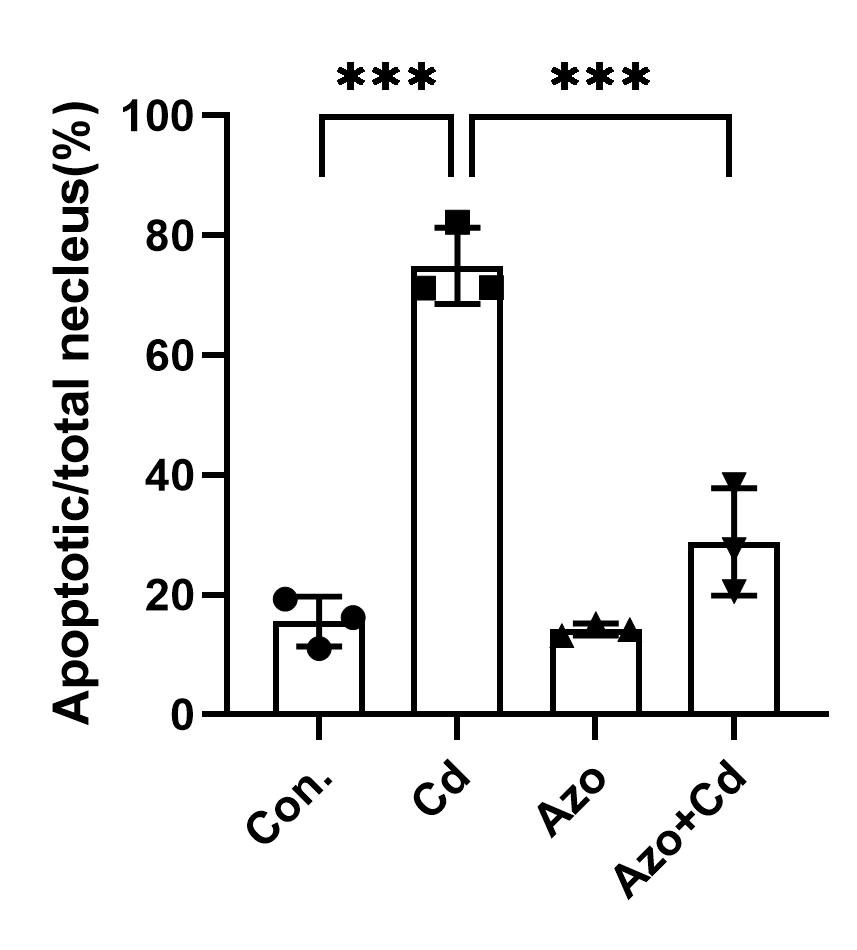

Supplement: Supplemental Information 1 — Files in .PZFX format can be accessed using GraphPad Prism software (version 9.0; San Diego, CA, USA) which can be downloaded from https://www.graphpad.com/. [file peerj-12-16844-s001.zip › Supplemental Files/Fig 2B/Fig 2B RPE DAPI staining analysis.tif]

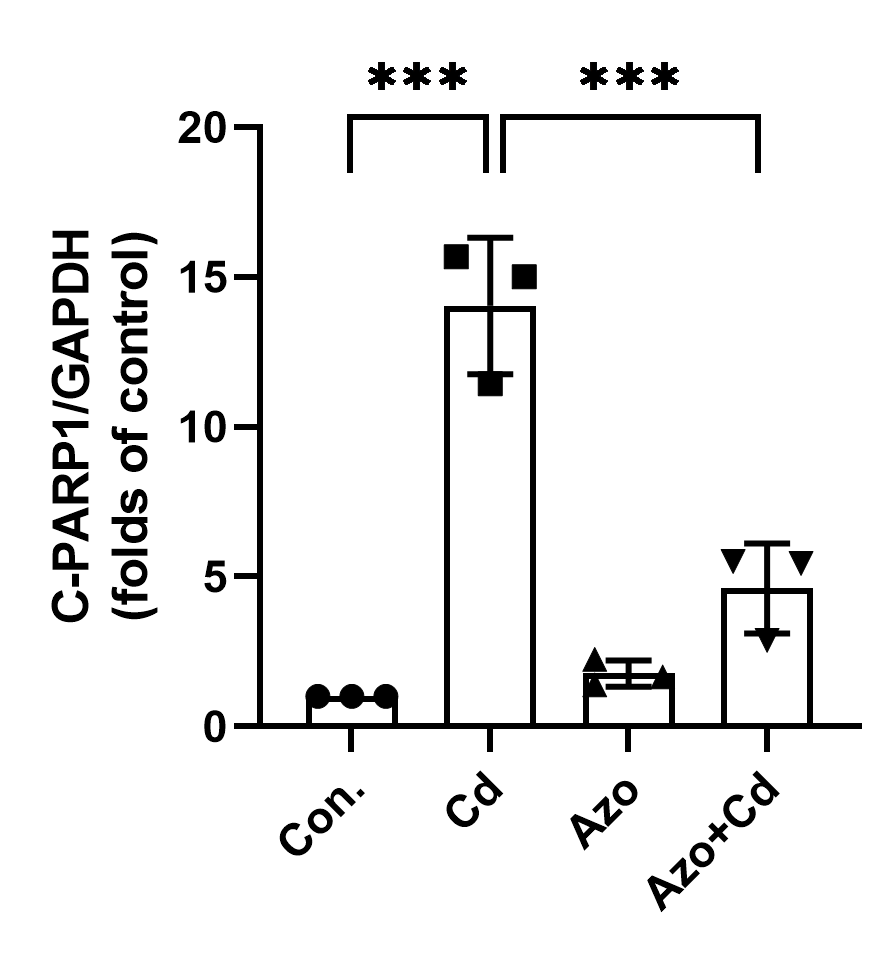

Supplement: Supplemental Information 1 — Files in .PZFX format can be accessed using GraphPad Prism software (version 9.0; San Diego, CA, USA) which can be downloaded from https://www.graphpad.com/. [file peerj-12-16844-s001.zip › Supplemental Files/Fig 2C/Fig 2C CPARP1.tif]

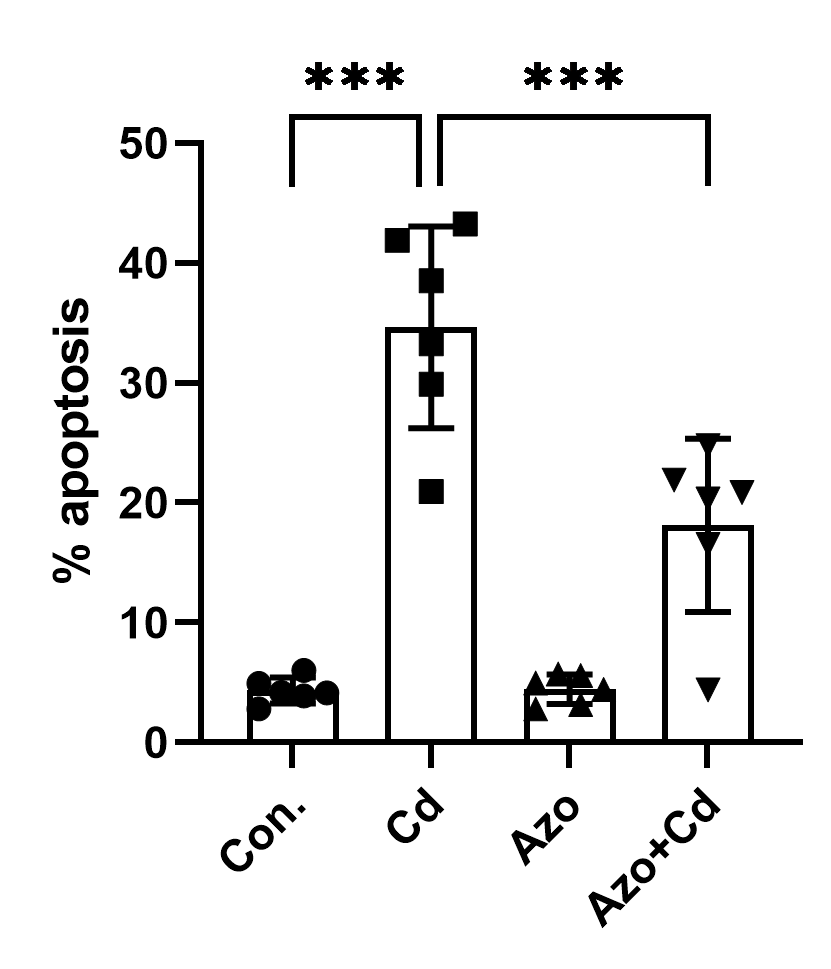

Supplement: Supplemental Information 1 — Files in .PZFX format can be accessed using GraphPad Prism software (version 9.0; San Diego, CA, USA) which can be downloaded from https://www.graphpad.com/. [file peerj-12-16844-s001.zip › Supplemental Files/Fig 2E/Fig 2E HK-2.tif]

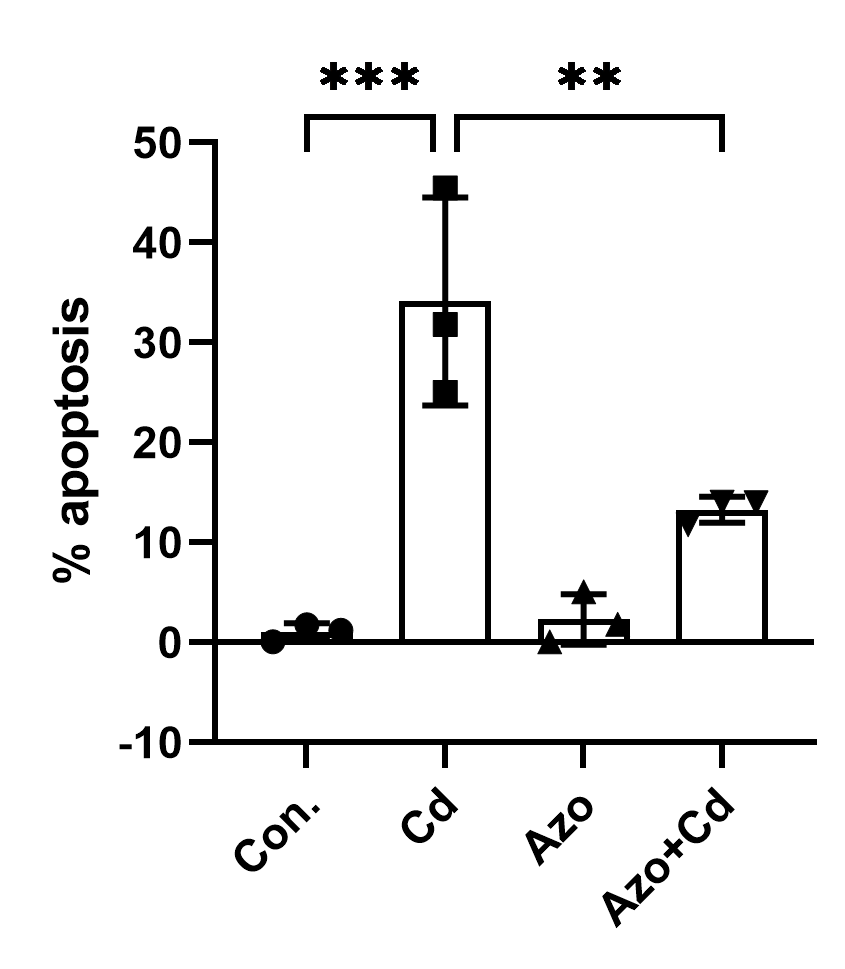

Supplement: Supplemental Information 1 — Files in .PZFX format can be accessed using GraphPad Prism software (version 9.0; San Diego, CA, USA) which can be downloaded from https://www.graphpad.com/. [file peerj-12-16844-s001.zip › Supplemental Files/Fig 2E/Fig 2E RPE.tif]

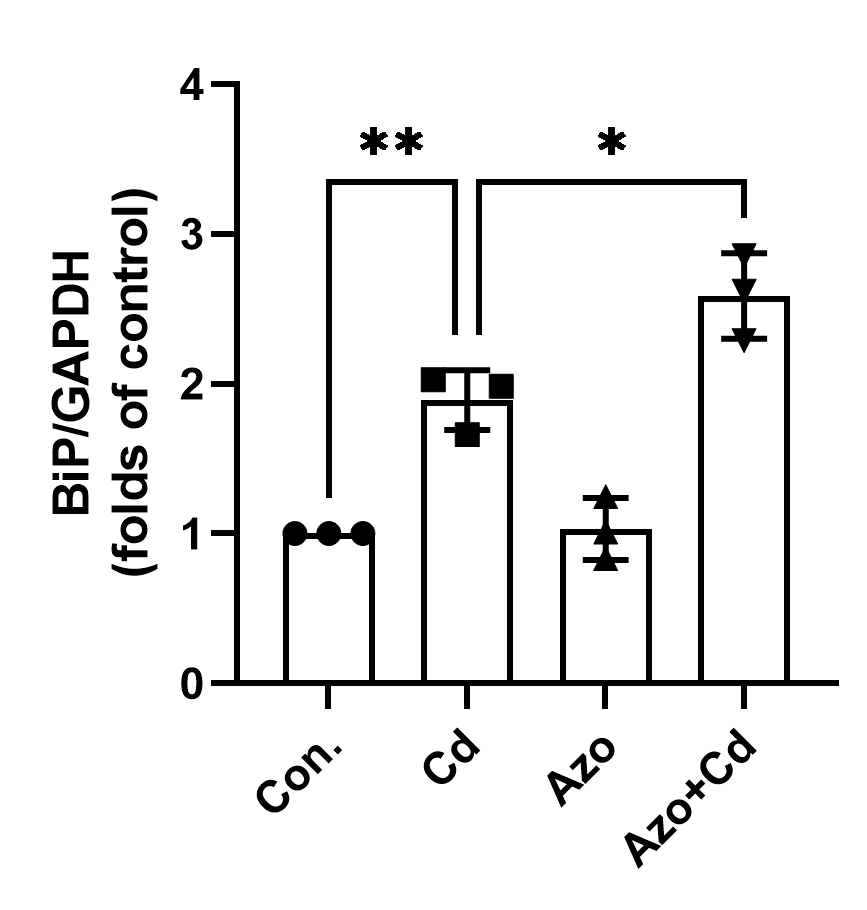

Supplement: Supplemental Information 1 — Files in .PZFX format can be accessed using GraphPad Prism software (version 9.0; San Diego, CA, USA) which can be downloaded from https://www.graphpad.com/. [file peerj-12-16844-s001.zip › Supplemental Files/Fig 3A/Fig 3A HK-2 BiP.tif]

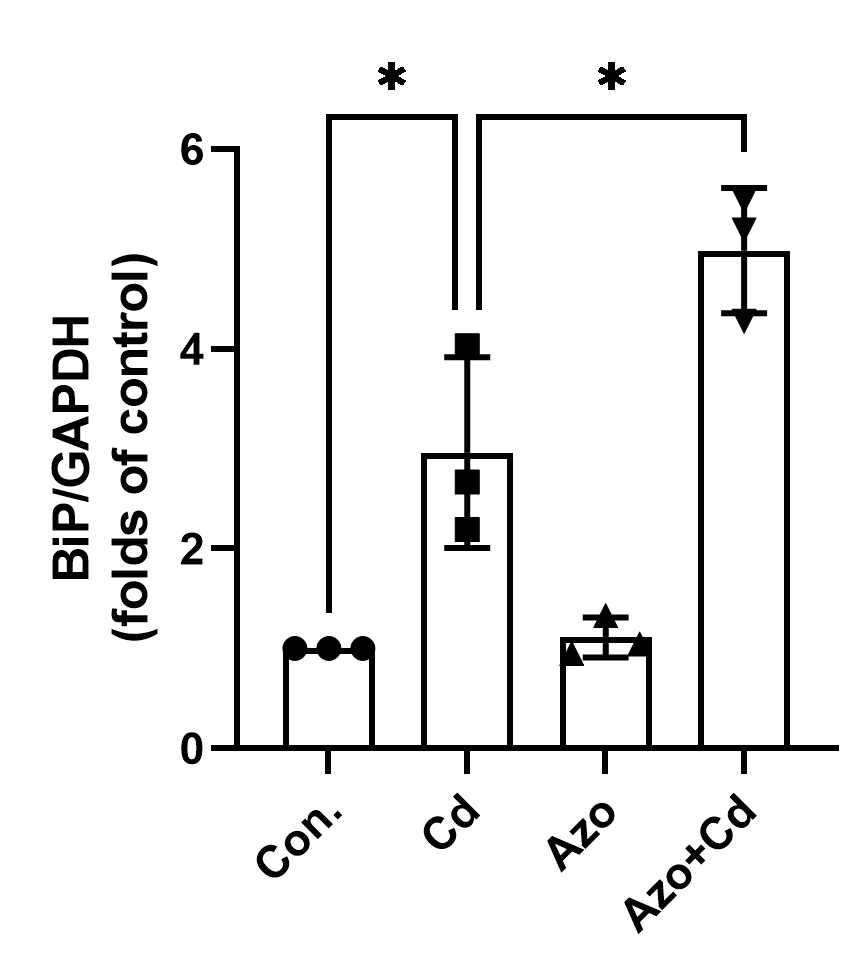

Supplement: Supplemental Information 1 — Files in .PZFX format can be accessed using GraphPad Prism software (version 9.0; San Diego, CA, USA) which can be downloaded from https://www.graphpad.com/. [file peerj-12-16844-s001.zip › Supplemental Files/Fig 3B/Fig 3B RPE BiP.tif]

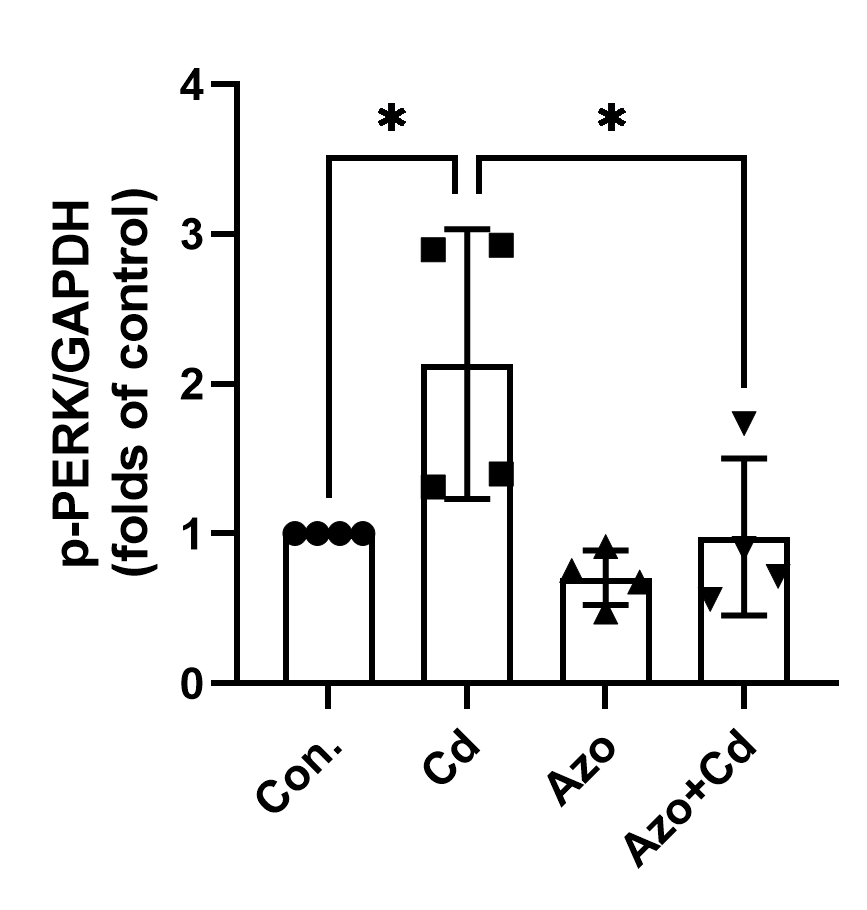

Supplement: Supplemental Information 1 — Files in .PZFX format can be accessed using GraphPad Prism software (version 9.0; San Diego, CA, USA) which can be downloaded from https://www.graphpad.com/. [file peerj-12-16844-s001.zip › Supplemental Files/Fig 3C/Fig 3C HK-2 p-PERK.tif]

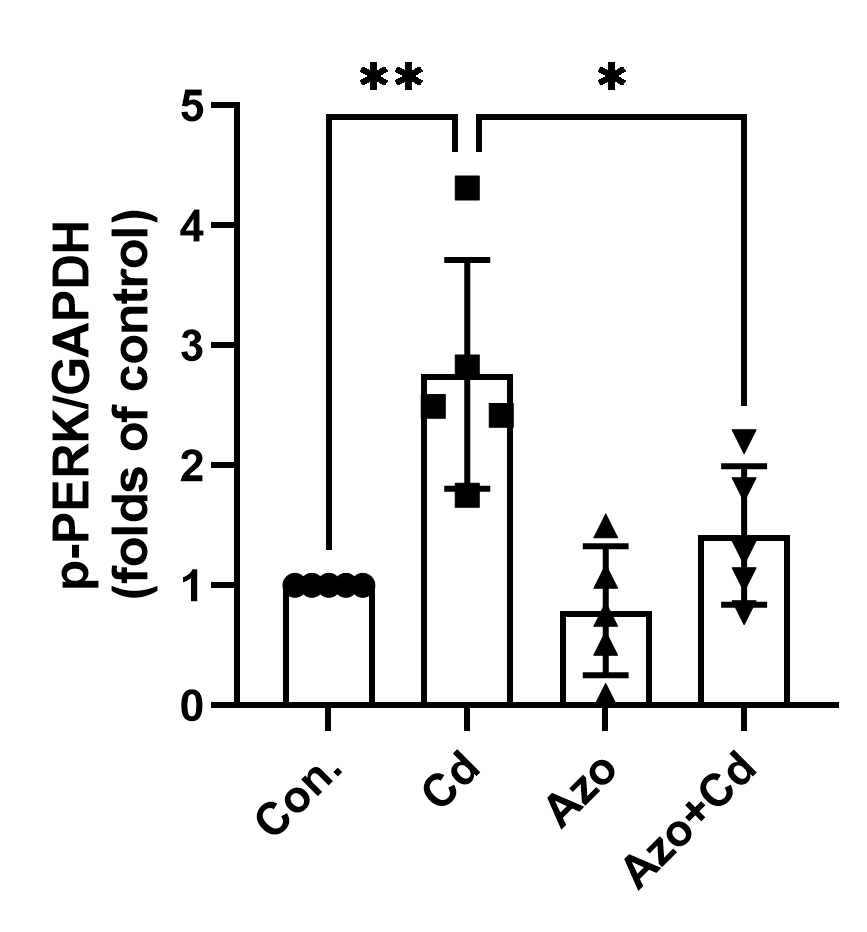

Supplement: Supplemental Information 1 — Files in .PZFX format can be accessed using GraphPad Prism software (version 9.0; San Diego, CA, USA) which can be downloaded from https://www.graphpad.com/. [file peerj-12-16844-s001.zip › Supplemental Files/Fig 3D/Fig 3D RPE p-PERK.tif]

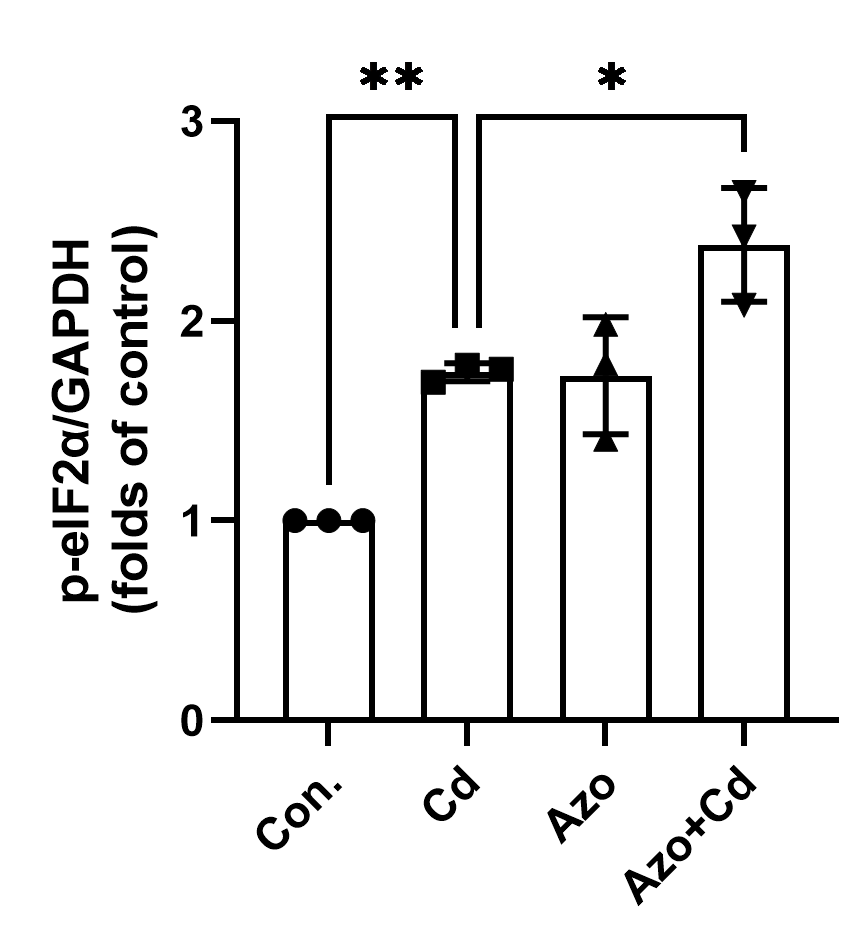

Supplement: Supplemental Information 1 — Files in .PZFX format can be accessed using GraphPad Prism software (version 9.0; San Diego, CA, USA) which can be downloaded from https://www.graphpad.com/. [file peerj-12-16844-s001.zip › Supplemental Files/Fig 3E/Fig 3E HK-2 p-eIF2α.tif]

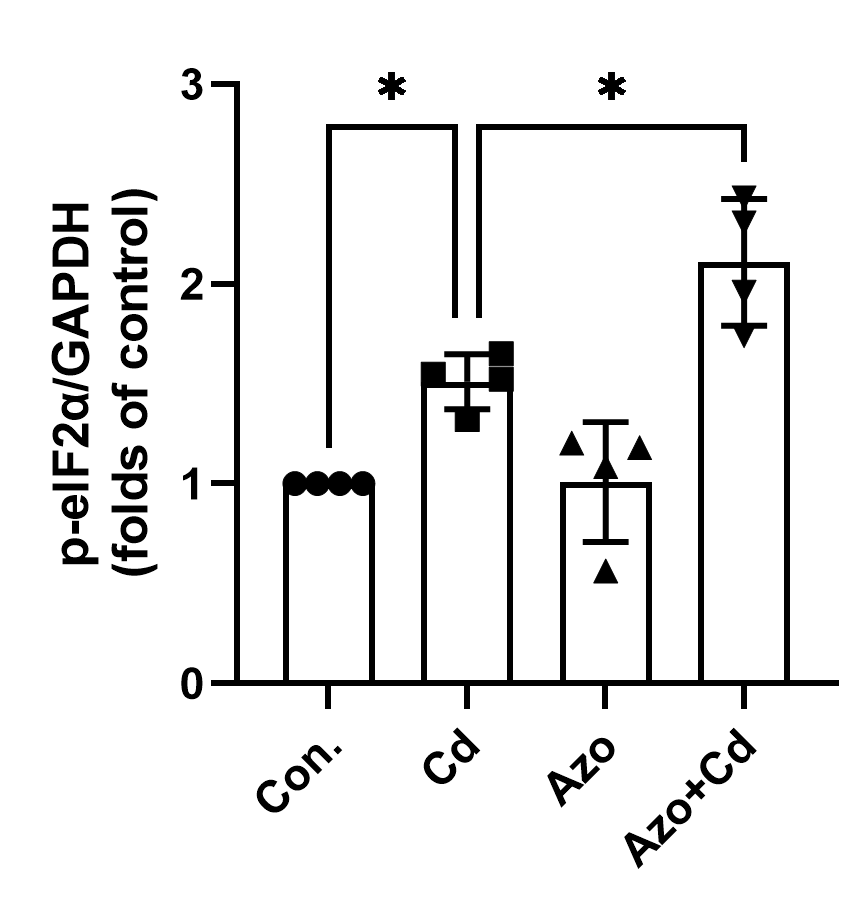

Supplement: Supplemental Information 1 — Files in .PZFX format can be accessed using GraphPad Prism software (version 9.0; San Diego, CA, USA) which can be downloaded from https://www.graphpad.com/. [file peerj-12-16844-s001.zip › Supplemental Files/Fig 3F/Fig 3F RPE p-eIF2α.tif]

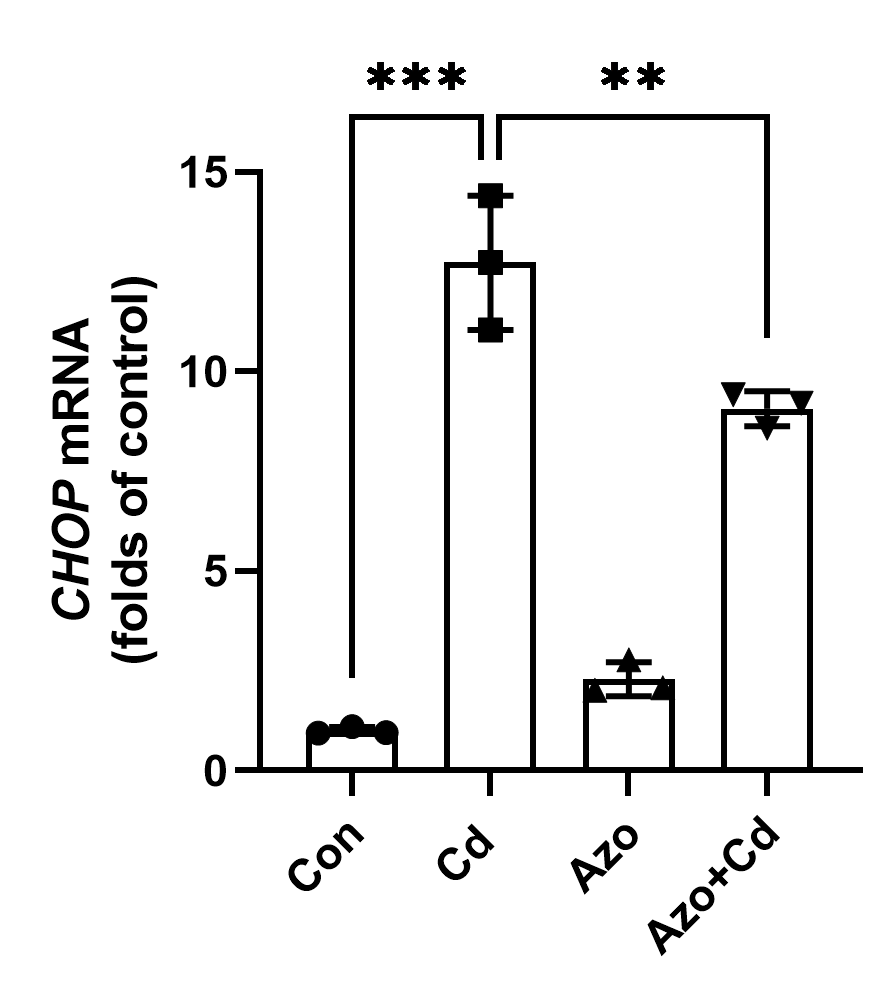

Supplement: Supplemental Information 1 — Files in .PZFX format can be accessed using GraphPad Prism software (version 9.0; San Diego, CA, USA) which can be downloaded from https://www.graphpad.com/. [file peerj-12-16844-s001.zip › Supplemental Files/Fig 3G/Fig 3G HK-2 CHOP.tif]

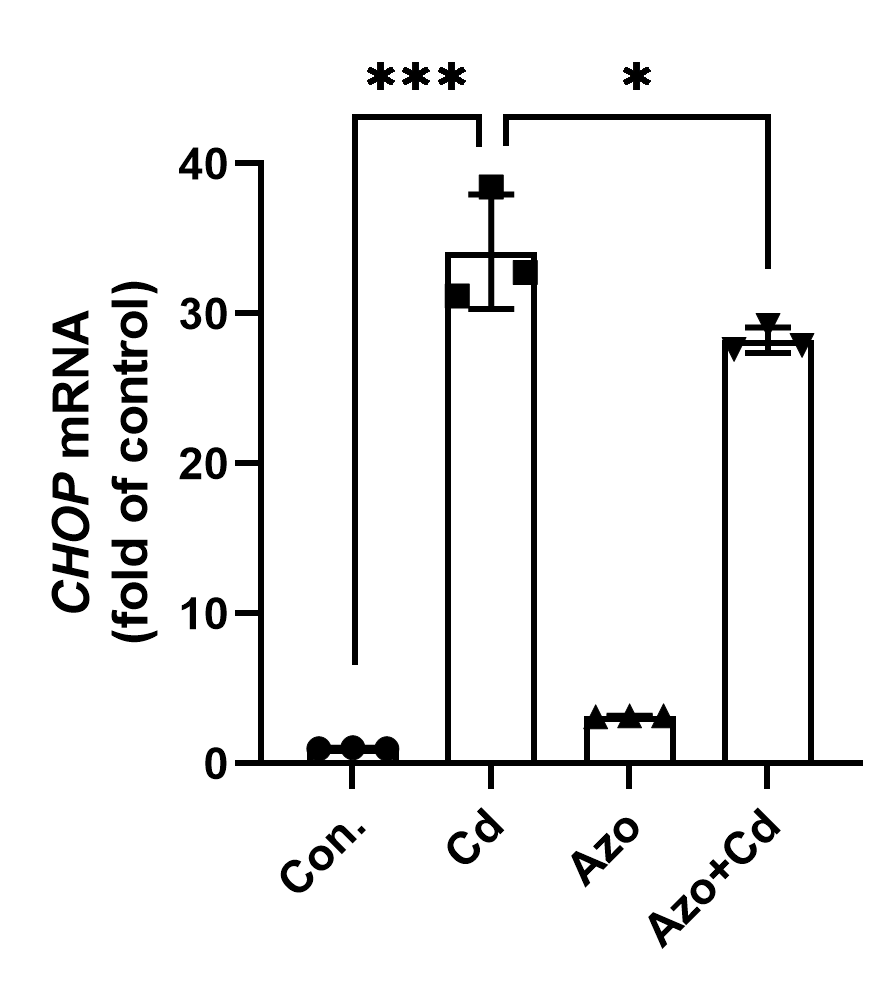

Supplement: Supplemental Information 1 — Files in .PZFX format can be accessed using GraphPad Prism software (version 9.0; San Diego, CA, USA) which can be downloaded from https://www.graphpad.com/. [file peerj-12-16844-s001.zip › Supplemental Files/Fig 3H/Fig 3H RPE CHOP.tif]

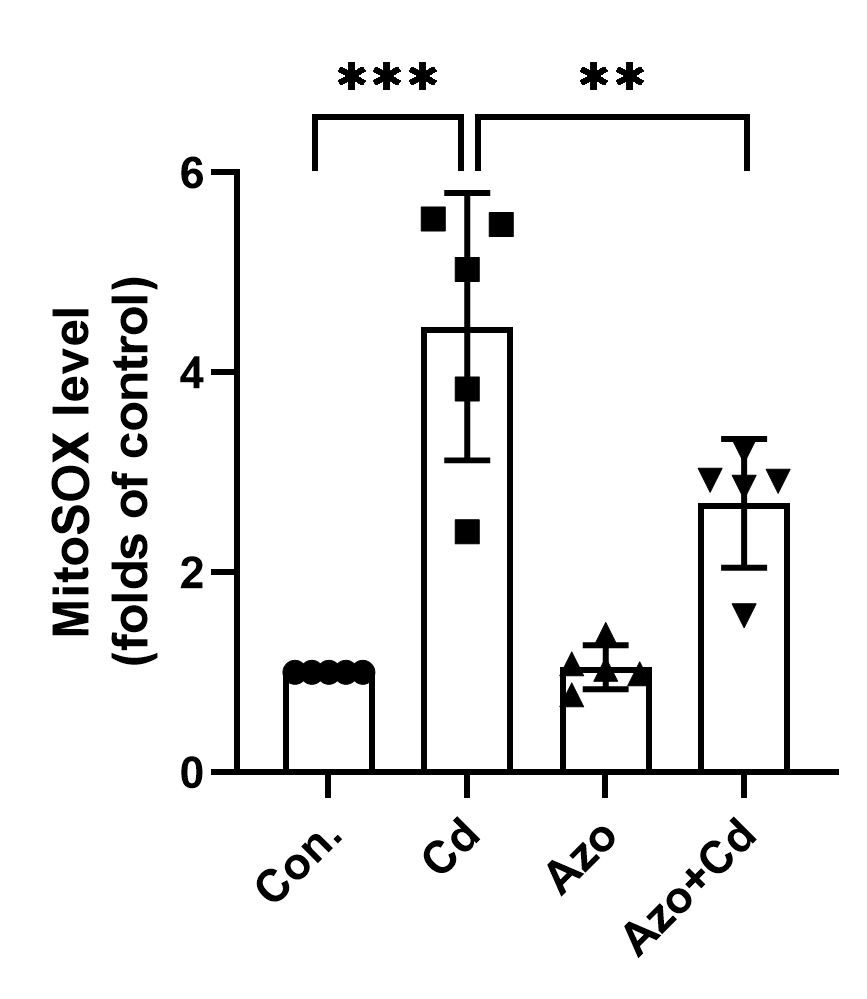

Supplement: Supplemental Information 1 — Files in .PZFX format can be accessed using GraphPad Prism software (version 9.0; San Diego, CA, USA) which can be downloaded from https://www.graphpad.com/. [file peerj-12-16844-s001.zip › Supplemental Files/Fig 4A/Fig 4A HK-2 MitoSOX.tif]

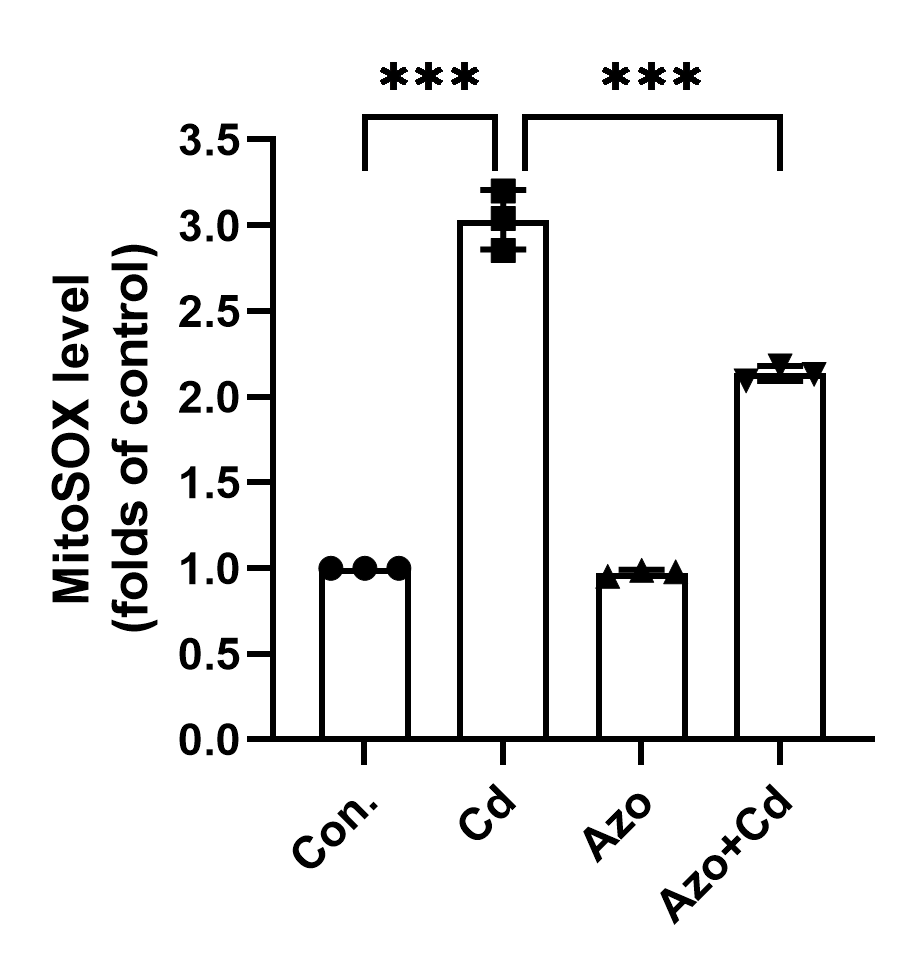

Supplement: Supplemental Information 1 — Files in .PZFX format can be accessed using GraphPad Prism software (version 9.0; San Diego, CA, USA) which can be downloaded from https://www.graphpad.com/. [file peerj-12-16844-s001.zip › Supplemental Files/Fig 4B/Fig 4B RPE MitoSOX.tif]

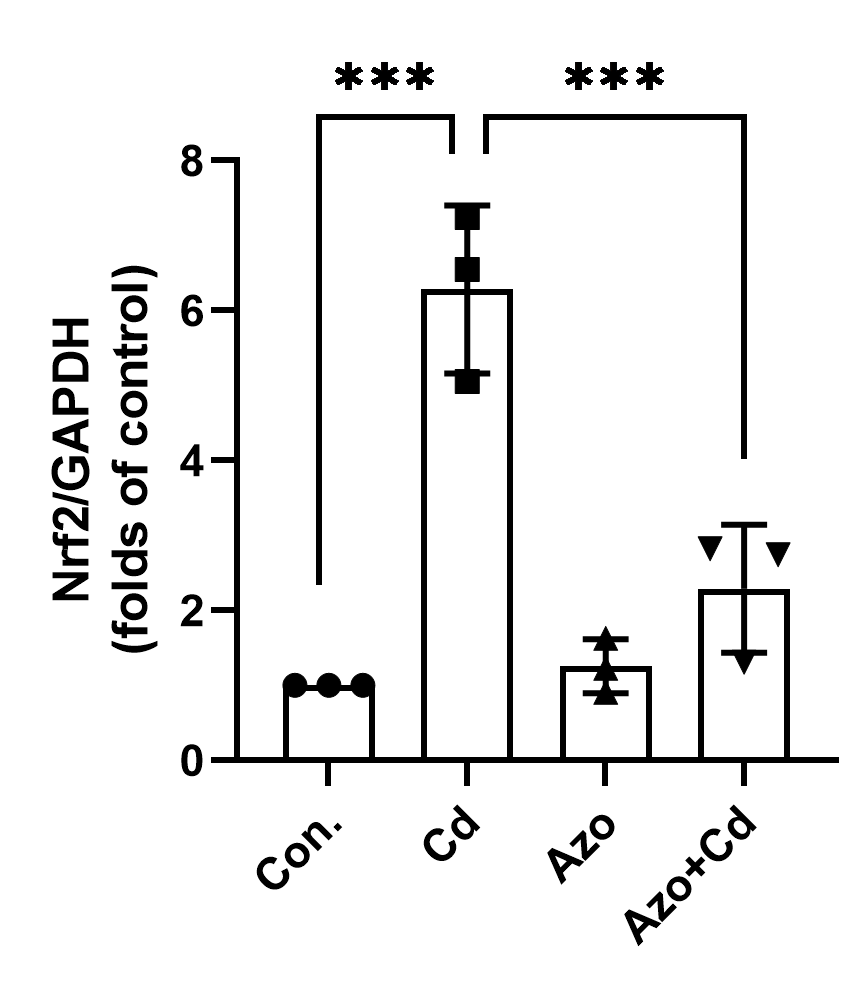

Supplement: Supplemental Information 1 — Files in .PZFX format can be accessed using GraphPad Prism software (version 9.0; San Diego, CA, USA) which can be downloaded from https://www.graphpad.com/. [file peerj-12-16844-s001.zip › Supplemental Files/Fig 4C/Fig 4C HK-2 Nrf2.tif]

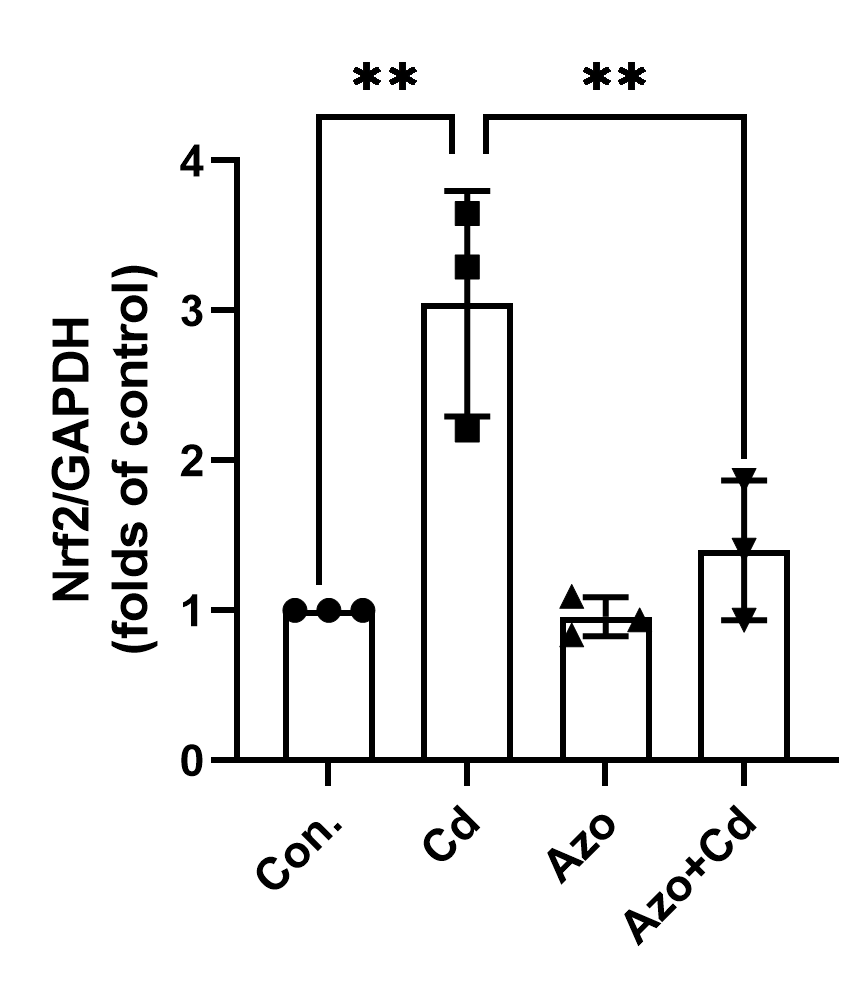

Supplement: Supplemental Information 1 — Files in .PZFX format can be accessed using GraphPad Prism software (version 9.0; San Diego, CA, USA) which can be downloaded from https://www.graphpad.com/. [file peerj-12-16844-s001.zip › Supplemental Files/Fig 4D/Fig 4D RPE Nrf2.tif]

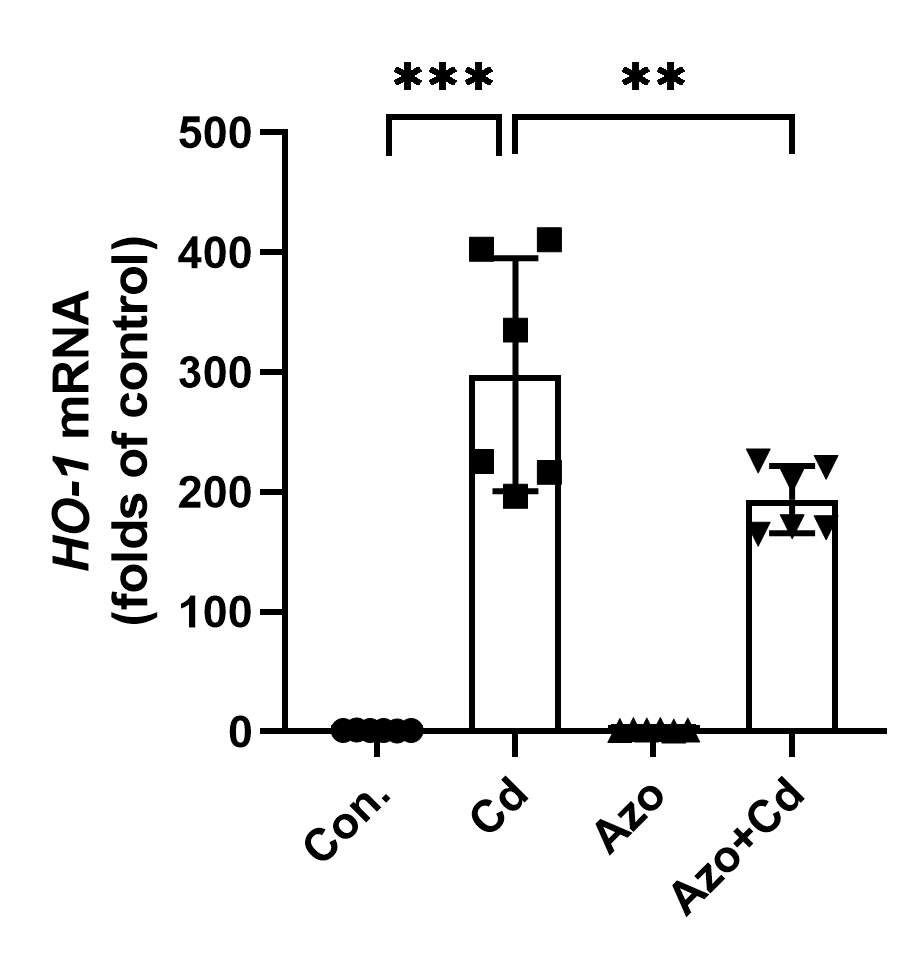

Supplement: Supplemental Information 1 — Files in .PZFX format can be accessed using GraphPad Prism software (version 9.0; San Diego, CA, USA) which can be downloaded from https://www.graphpad.com/. [file peerj-12-16844-s001.zip › Supplemental Files/Fig 4E/Fig 4E HK-2 HO-1 new.tif]

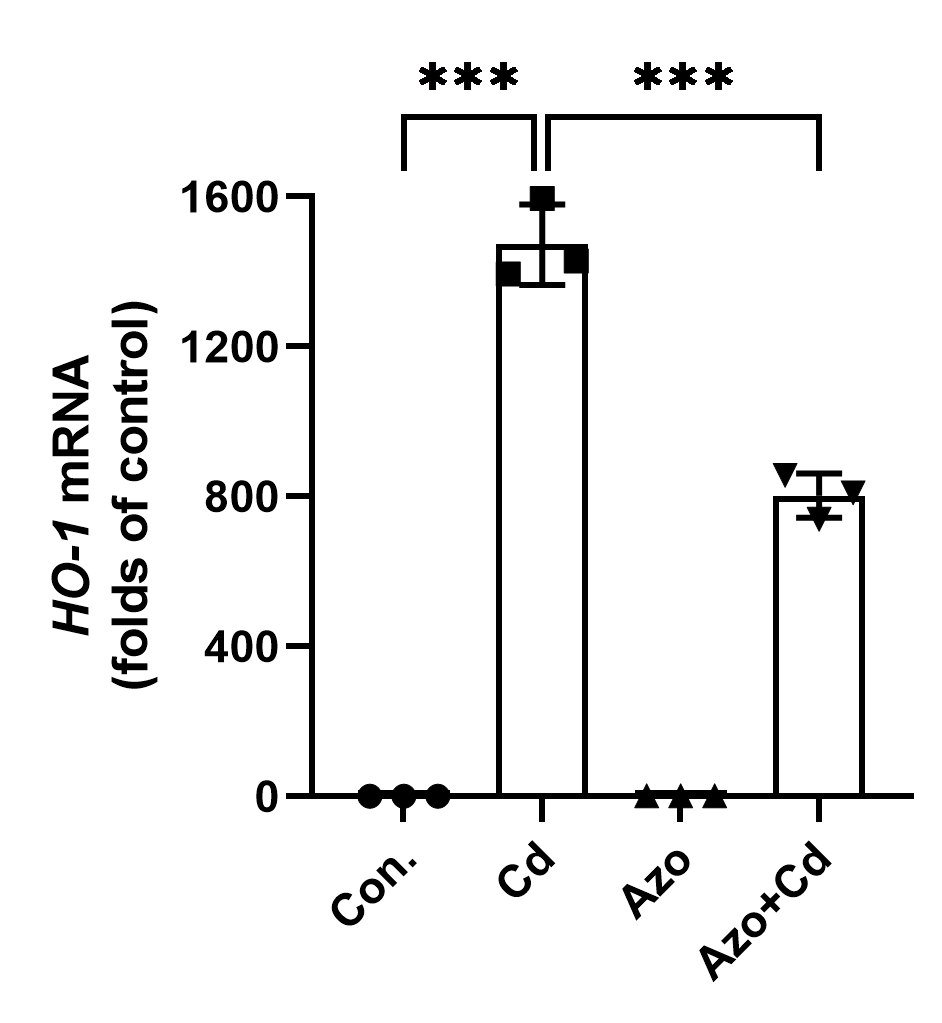

Supplement: Supplemental Information 1 — Files in .PZFX format can be accessed using GraphPad Prism software (version 9.0; San Diego, CA, USA) which can be downloaded from https://www.graphpad.com/. [file peerj-12-16844-s001.zip › Supplemental Files/Fig 4F/Fig 4F RPE HO-1 new.tif]

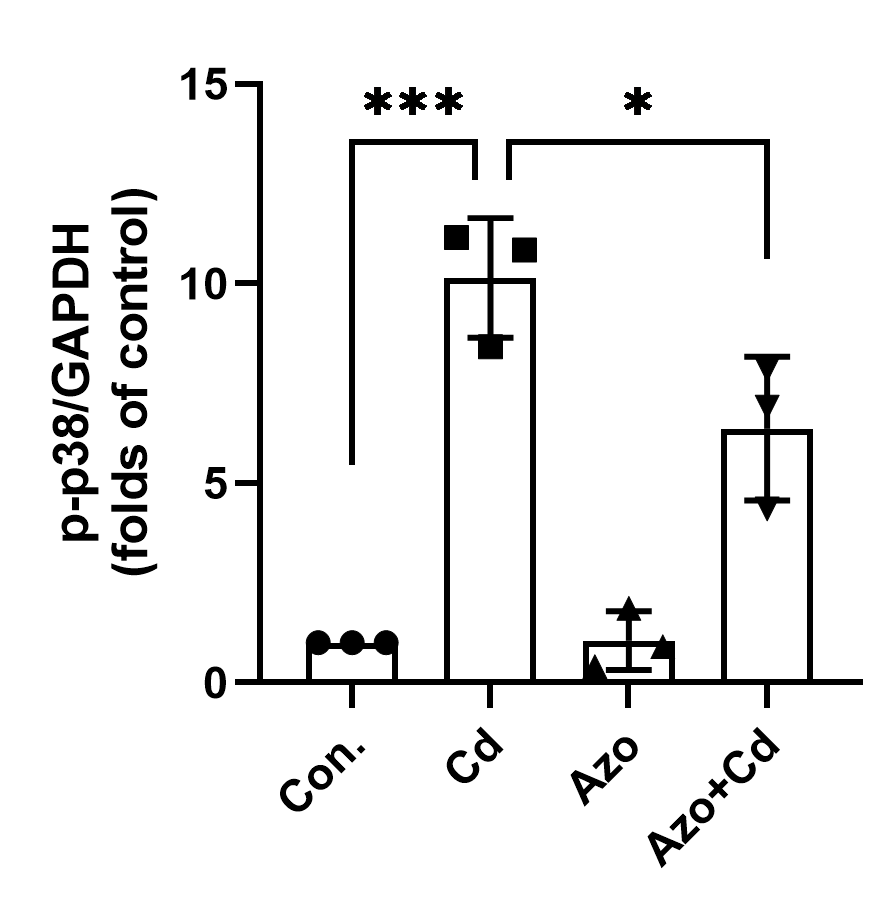

Supplement: Supplemental Information 1 — Files in .PZFX format can be accessed using GraphPad Prism software (version 9.0; San Diego, CA, USA) which can be downloaded from https://www.graphpad.com/. [file peerj-12-16844-s001.zip › Supplemental Files/Fig 5A/Fig 5A HK-2 p-p38.tif]

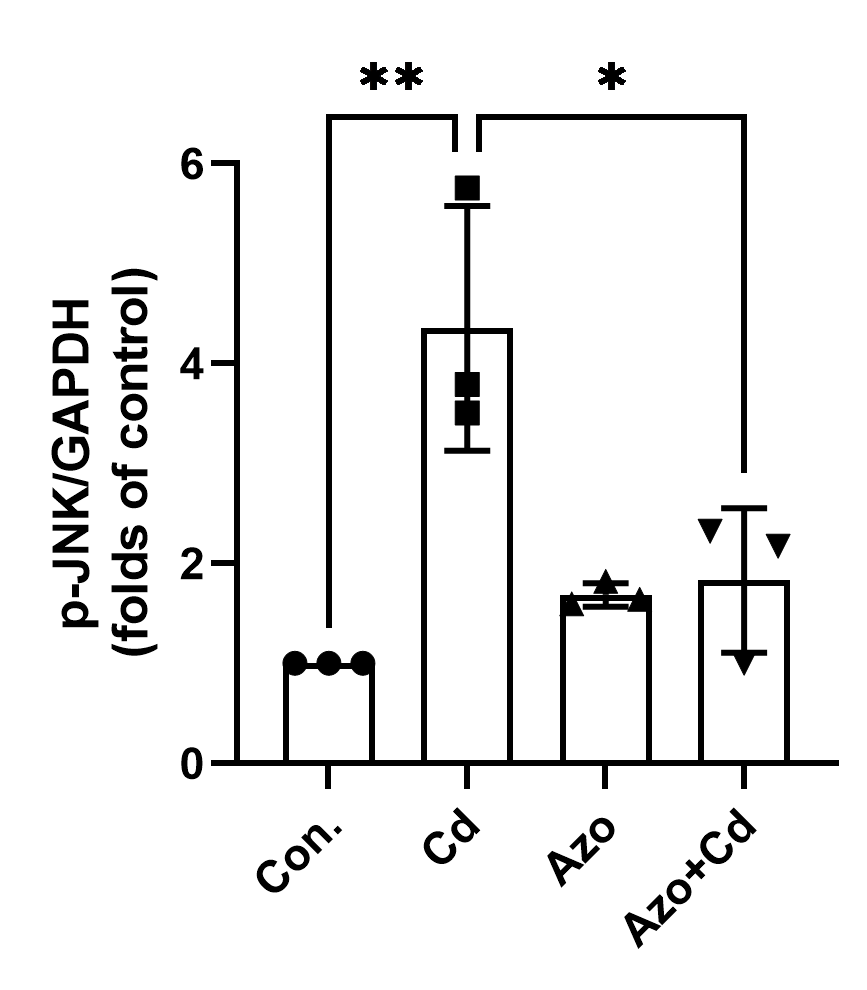

Supplement: Supplemental Information 1 — Files in .PZFX format can be accessed using GraphPad Prism software (version 9.0; San Diego, CA, USA) which can be downloaded from https://www.graphpad.com/. [file peerj-12-16844-s001.zip › Supplemental Files/Fig 5B/Fig 5B p-JNK.tif]

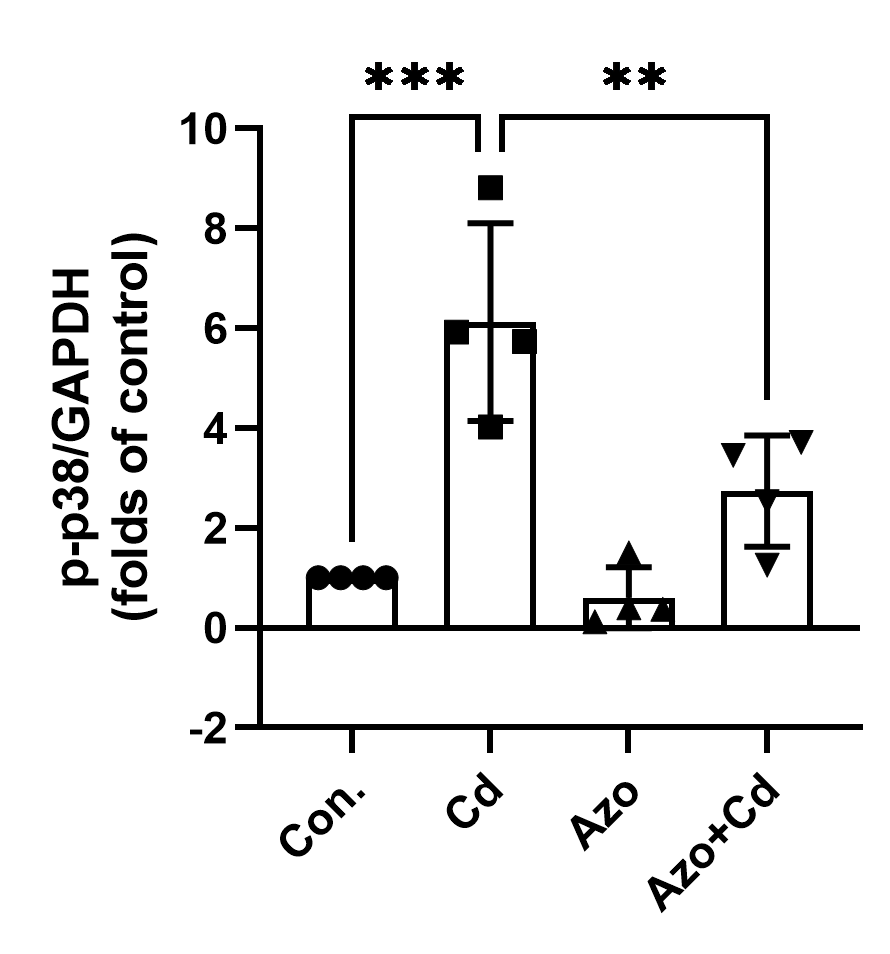

Supplement: Supplemental Information 1 — Files in .PZFX format can be accessed using GraphPad Prism software (version 9.0; San Diego, CA, USA) which can be downloaded from https://www.graphpad.com/. [file peerj-12-16844-s001.zip › Supplemental Files/Fig 5C/Fig 5C RPE p-p38.tif]

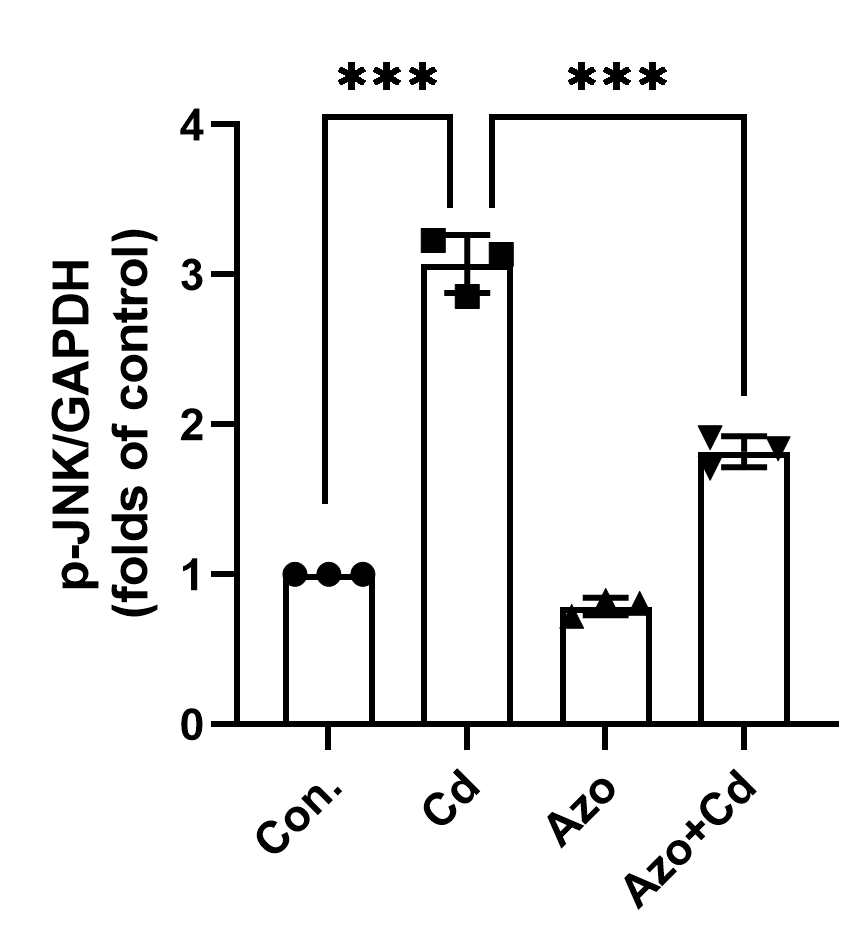

Supplement: Supplemental Information 1 — Files in .PZFX format can be accessed using GraphPad Prism software (version 9.0; San Diego, CA, USA) which can be downloaded from https://www.graphpad.com/. [file peerj-12-16844-s001.zip › Supplemental Files/Fig 5D/Fig 5D RPE p-JNK.tif]

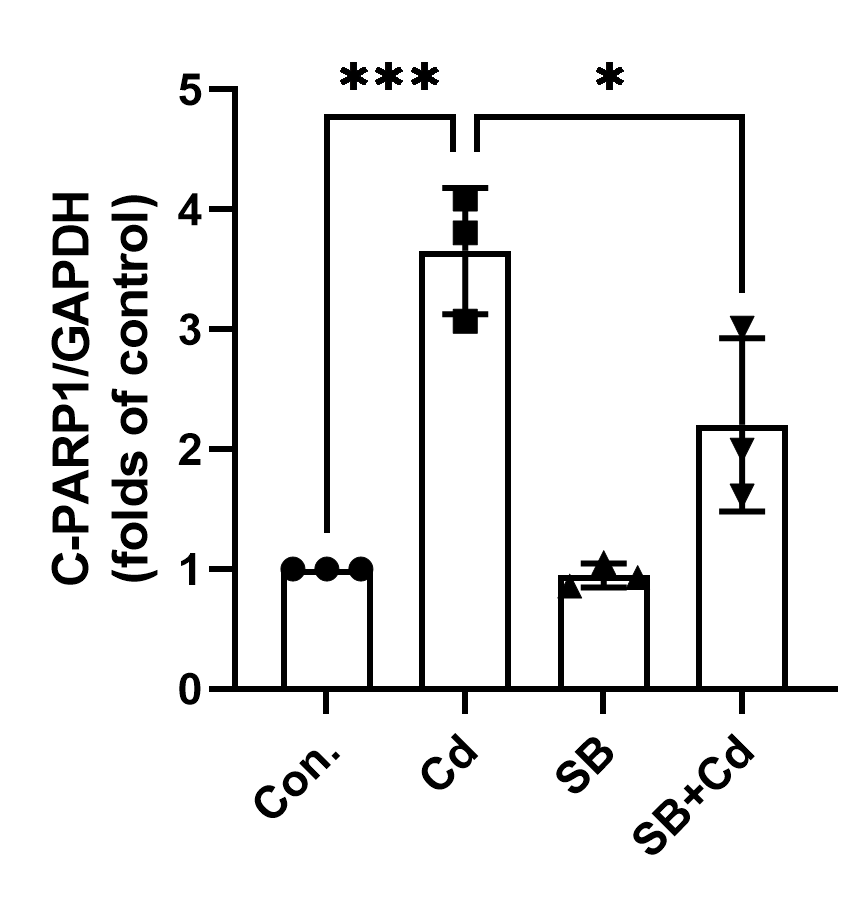

Supplement: Supplemental Information 1 — Files in .PZFX format can be accessed using GraphPad Prism software (version 9.0; San Diego, CA, USA) which can be downloaded from https://www.graphpad.com/. [file peerj-12-16844-s001.zip › Supplemental Files/Fig 5E/Fig 5E HK-2 SB+Cd C-PARP1.tif]

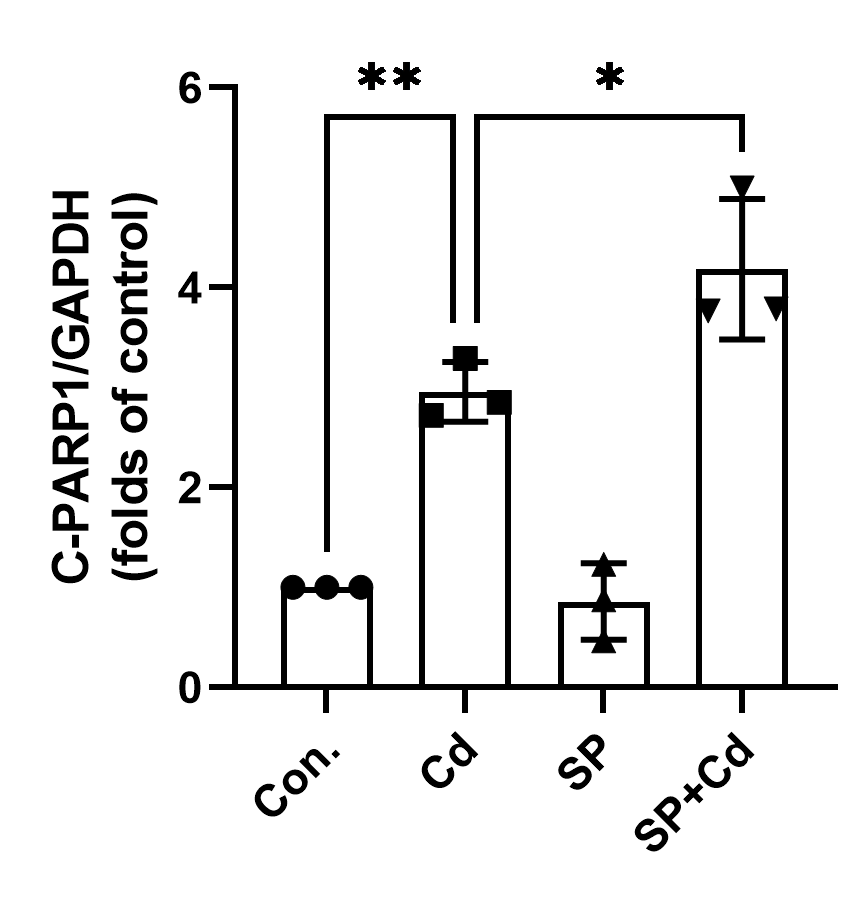

Supplement: Supplemental Information 1 — Files in .PZFX format can be accessed using GraphPad Prism software (version 9.0; San Diego, CA, USA) which can be downloaded from https://www.graphpad.com/. [file peerj-12-16844-s001.zip › Supplemental Files/Fig 5F/Fig 5F HK-2 SP+Cd C-PARP1.tif]

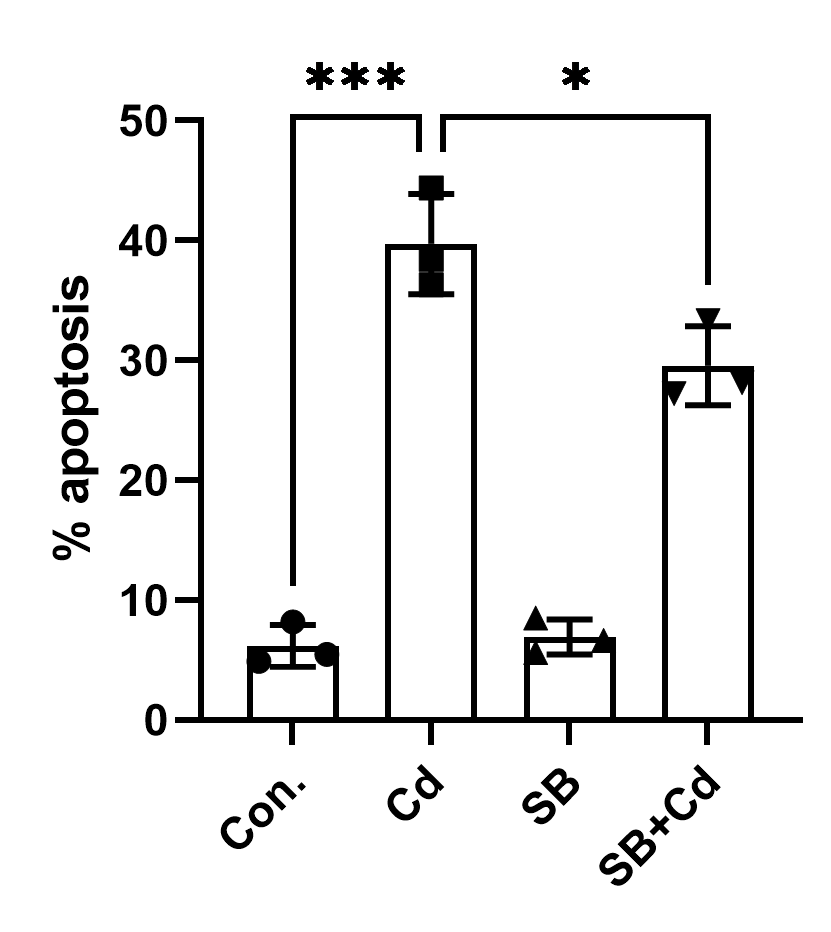

Supplement: Supplemental Information 1 — Files in .PZFX format can be accessed using GraphPad Prism software (version 9.0; San Diego, CA, USA) which can be downloaded from https://www.graphpad.com/. [file peerj-12-16844-s001.zip › Supplemental Files/Fig 5G/Fig 5G HK-2 Cd20+SB10 apoptosis rate.tif]

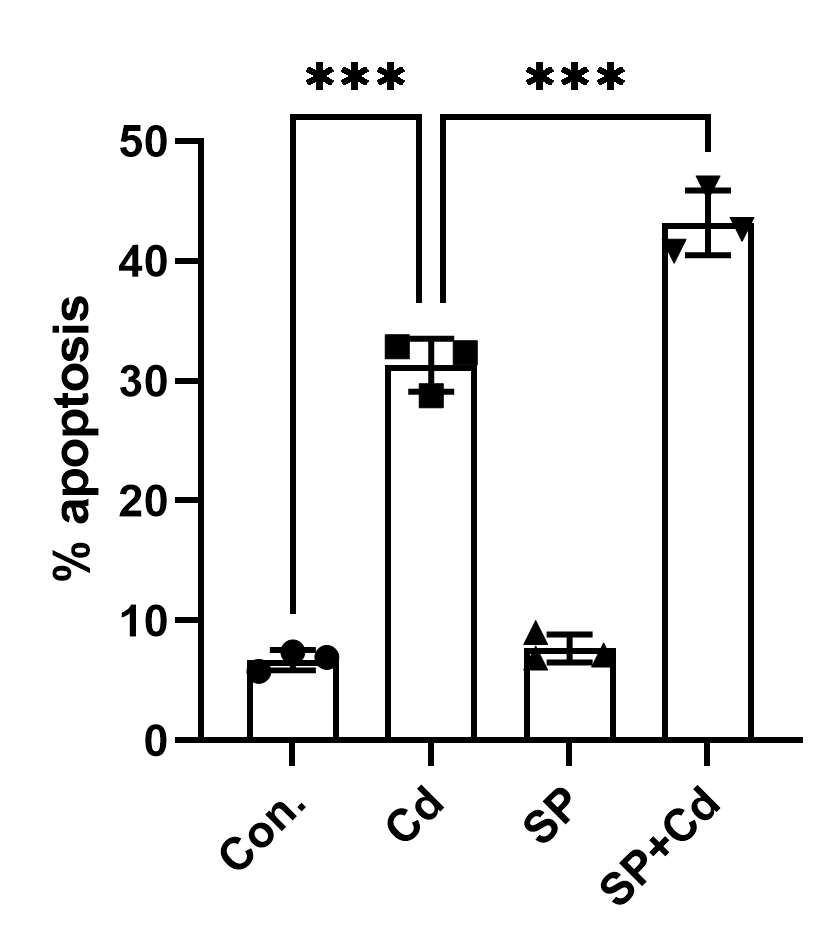

Supplement: Supplemental Information 1 — Files in .PZFX format can be accessed using GraphPad Prism software (version 9.0; San Diego, CA, USA) which can be downloaded from https://www.graphpad.com/. [file peerj-12-16844-s001.zip › Supplemental Files/Fig 5H/Fig 5H HK-2 Cd20+SP10 apoptosis rate.tif]

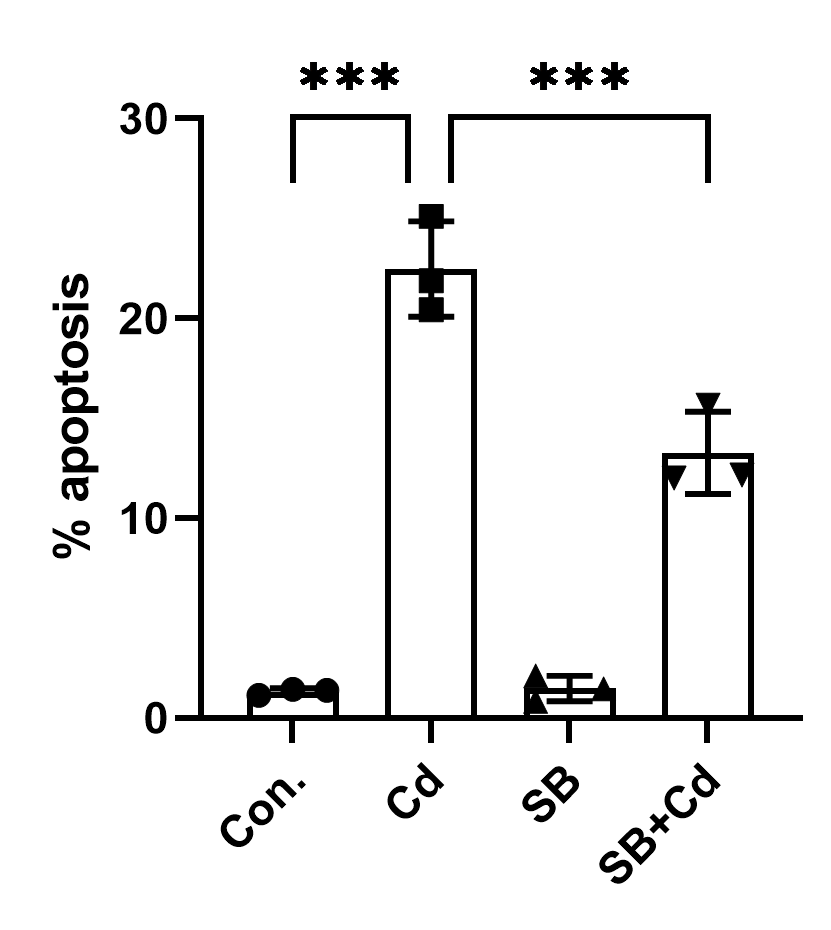

Supplement: Supplemental Information 1 — Files in .PZFX format can be accessed using GraphPad Prism software (version 9.0; San Diego, CA, USA) which can be downloaded from https://www.graphpad.com/. [file peerj-12-16844-s001.zip › Supplemental Files/Fig 5I/Fig 5I RPE SB+Cd-FC-apoptosis.tif]

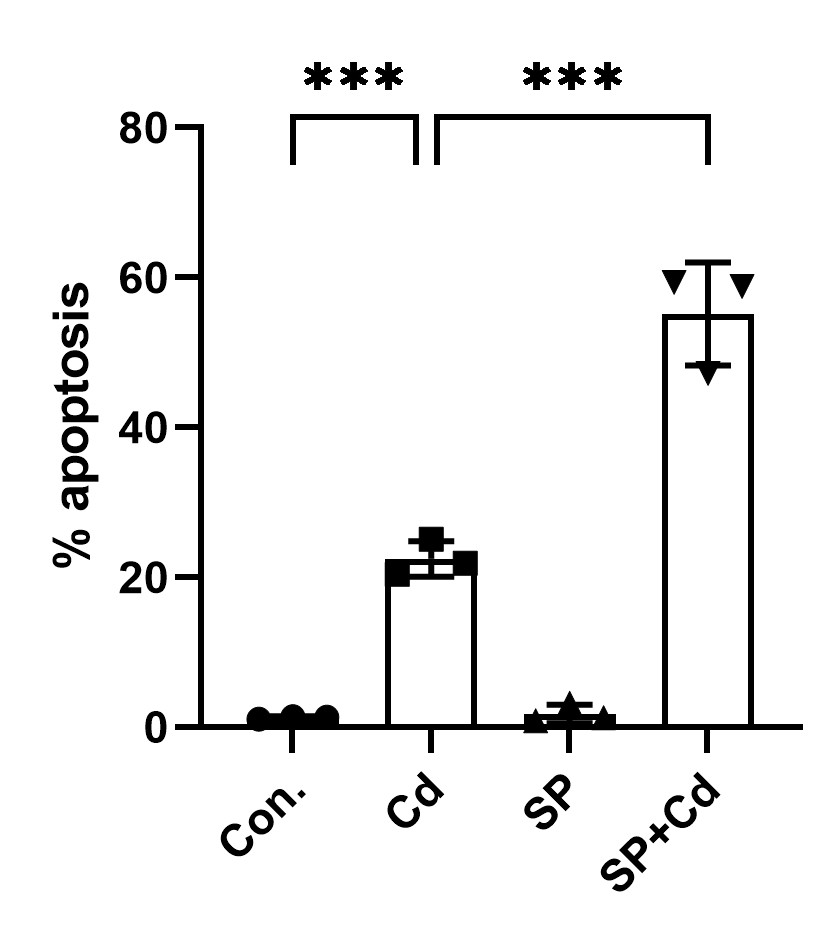

Supplement: Supplemental Information 1 — Files in .PZFX format can be accessed using GraphPad Prism software (version 9.0; San Diego, CA, USA) which can be downloaded from https://www.graphpad.com/. [file peerj-12-16844-s001.zip › Supplemental Files/Fig 5J/Fig 5J RPE SP+Cd-FC-apoptosis.tif]

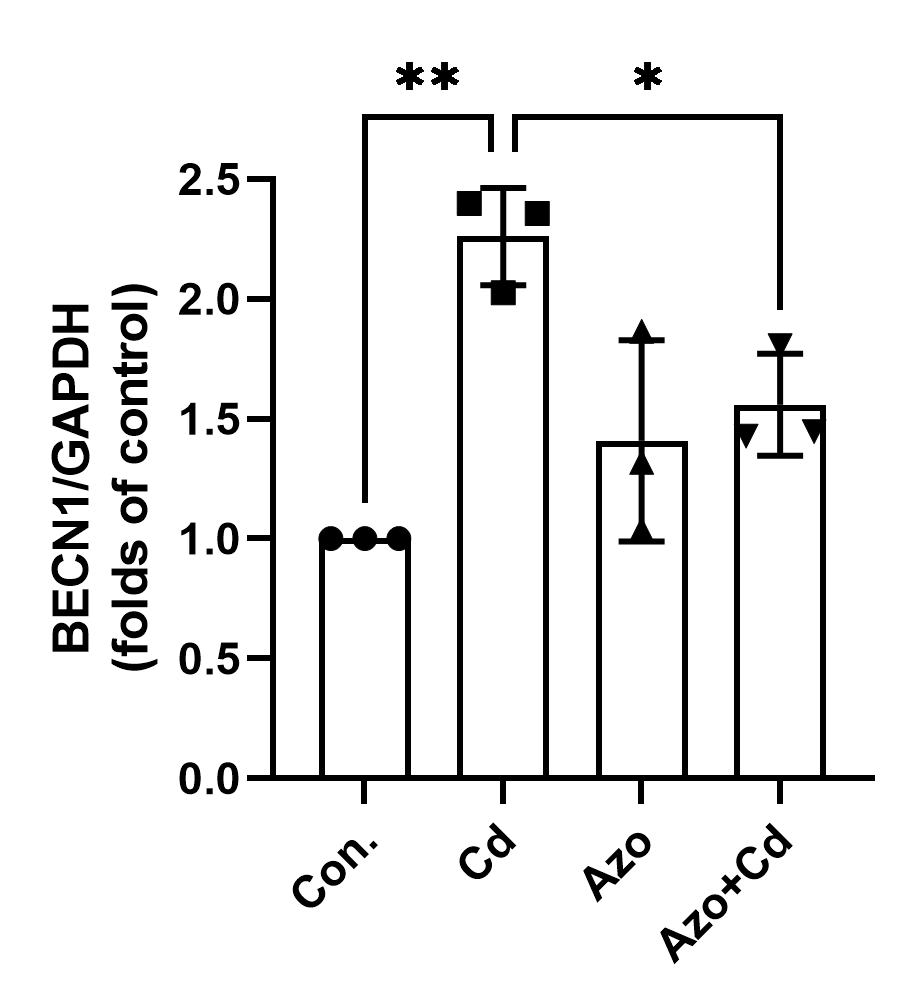

Supplement: Supplemental Information 1 — Files in .PZFX format can be accessed using GraphPad Prism software (version 9.0; San Diego, CA, USA) which can be downloaded from https://www.graphpad.com/. [file peerj-12-16844-s001.zip › Supplemental Files/Fig 6B/Fig 6B HK-2 BECN1.tif]

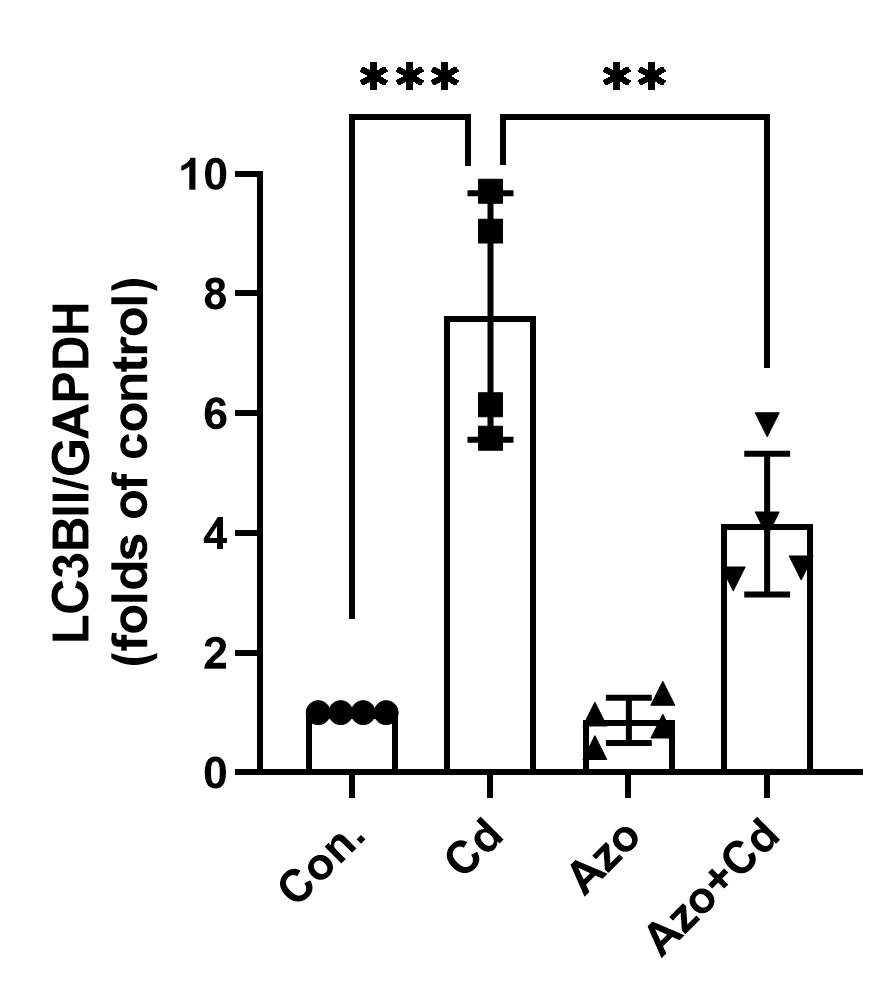

Supplement: Supplemental Information 1 — Files in .PZFX format can be accessed using GraphPad Prism software (version 9.0; San Diego, CA, USA) which can be downloaded from https://www.graphpad.com/. [file peerj-12-16844-s001.zip › Supplemental Files/Fig 6C/Fig 6C HK-2 LC3BII.tif]

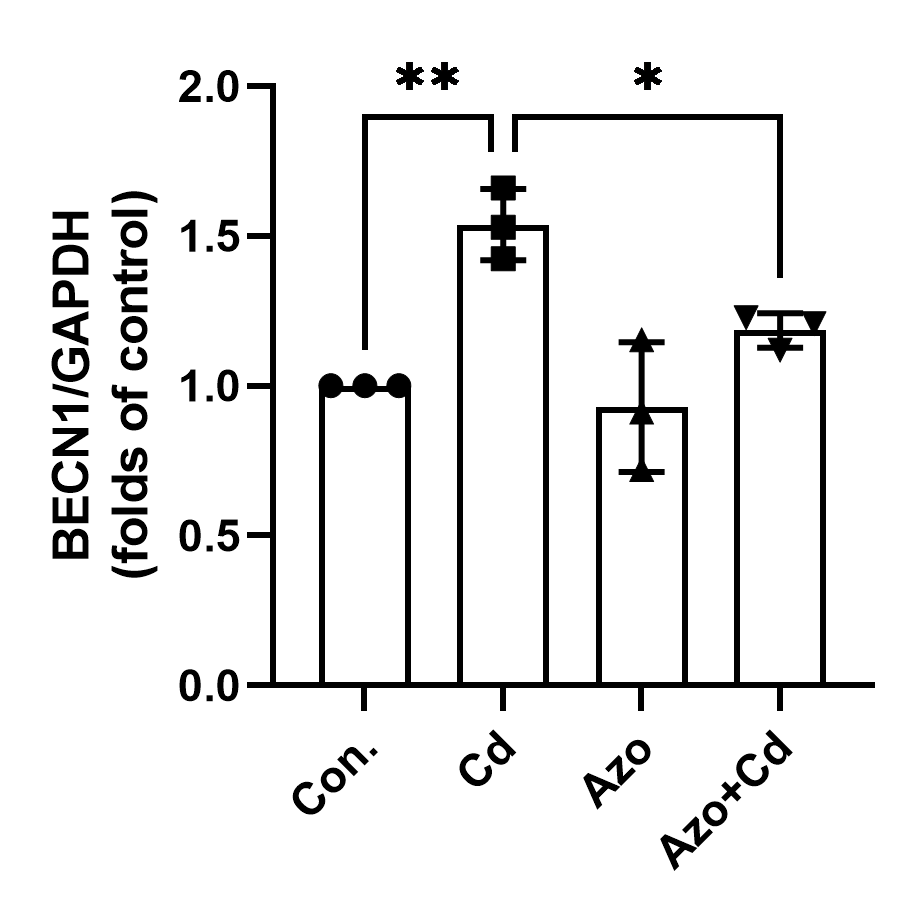

Supplement: Supplemental Information 1 — Files in .PZFX format can be accessed using GraphPad Prism software (version 9.0; San Diego, CA, USA) which can be downloaded from https://www.graphpad.com/. [file peerj-12-16844-s001.zip › Supplemental Files/Fig 6E/Fig 6E RPE BECN1.tif]

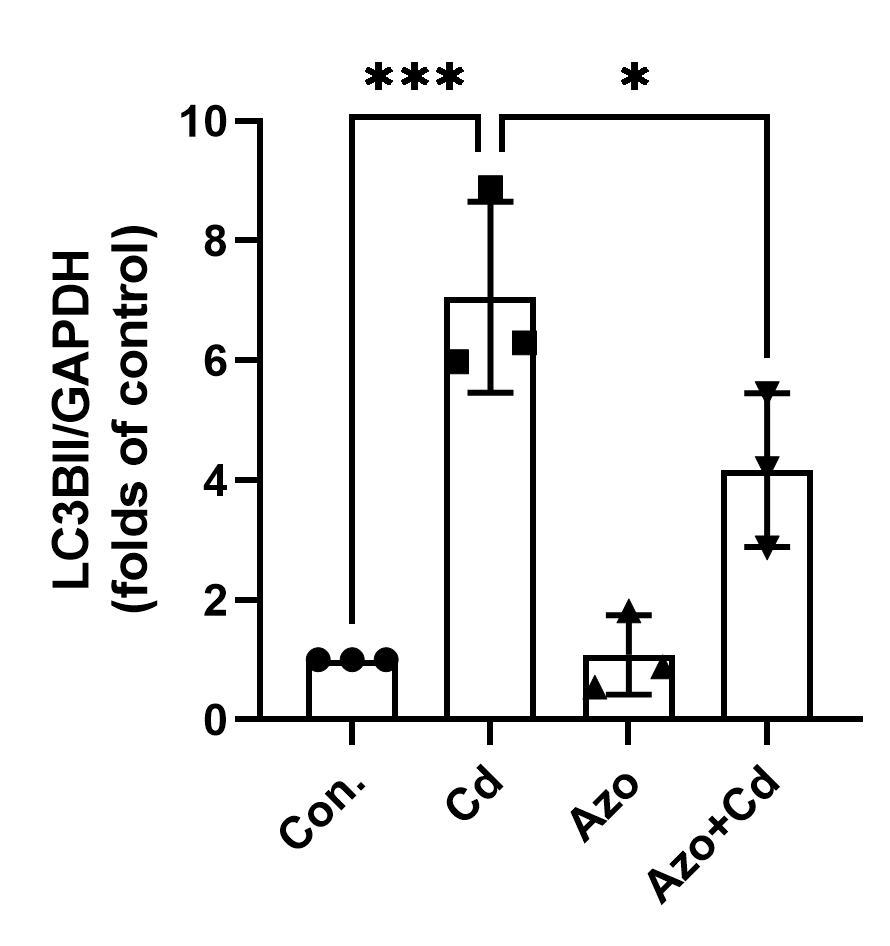

Supplement: Supplemental Information 1 — Files in .PZFX format can be accessed using GraphPad Prism software (version 9.0; San Diego, CA, USA) which can be downloaded from https://www.graphpad.com/. [file peerj-12-16844-s001.zip › Supplemental Files/Fig 6F/Fig 6F RPE LC3BII.tif]
